# Dynamics of rumen gene expression, microbiome colonization, and their interplay in goats

**DOI:** 10.1186/s12864-021-07595-1

**Published:** 2021-04-21

**Authors:** Xiangyu Pan, Zongjun Li, Bibo Li, Chen Zhao, Yu Wang, Yulin Chen, Yu Jiang

**Affiliations:** Key Laboratory of Animal Genetics, Breeding and Reproduction of Shaanxi Province, College of Animal Science and Technology, Northwest A&F University, Yangling, 712100 China

**Keywords:** Rumen development, Rumen transcriptome, Microbiome, Rumen-microbial crosstalk

## Abstract

**Background:**

Preweaned rumen development is vital for animal health and efficient fermentation. In this study, we integrated ruminal transcriptomic and metagenomic data to explore the dynamics of rumen functions, microbial colonization, and their functional interactions during the first 8 weeks of life in goats.

**Results:**

The dynamic rumen transcriptomic and microbial profiles both exhibited two distinct phases during early rumen development. The differentially expressed genes of the rumen transcriptome between the two phases showed that the immune-related response was enriched in the first phase and nutrient-related metabolism was enriched in the second phase, whereas the differentially expressed genes of the rumen microbiome were enriched in bacteriocin biosynthesis and glycolysis/gluconeogenesis activities. The developmental shift in the rumen transcriptome (at d 21) was earlier than the feed stimulus (at d 25) and the shift in the rumen microbiome (at d 42). Additionally, 15 temporal dynamic rumen gene modules and 20 microbial modules were revealed by coexpression network analysis. Functional correlations between the rumen and its microbiome were primarily involved in rumen pH homeostasis, nitrogen metabolism and the immune response. Rumen gene modules associated with the microbial alpha diversity index were also enriched in the immune response process.

**Conclusions:**

The present study touched the critical developmental process of rumen functions, microbial colonization and their functional interactions during preweaned development. Taken together, these results demonstrated that rumen development at the first phase is more likely a programmed process rather than stimulation from feed and the microbiome, while the shift of rumen metagenomes was likely regulated by both the diet and host. The intensive functional correlations between rumen genes and the microbiome demonstrated that synergistic processes occurred between them during early rumen development.

**Supplementary Information:**

The online version contains supplementary material available at 10.1186/s12864-021-07595-1.

## Background

Ruminant livestock has a complex digestive system that allows them to convert human-indigestible plant biomass into high-quality products such as milk and meat, due to complex microbiomes residing in the rumen. Promoting rumen functional development has always been a crucial target of neonatal livestock management [1]. The establishment of rumen function and its microbiota during postnatal development can be influenced by the changes in feeding management, such as weaning and diets [2]. The influences have impact on health and whole life performance of adult ruminants [1, 3, 4]. Previous studies have revealed that the significant physiological changes occurred in young ruminants can be divided into three phases, which are the non-ruminant stage from birth to 3 weeks old, the transition stage during weeks 3–8 and the rumination stage after 8 weeks [5–7]. The process from non-ruminant to ruminant is a key stage in the establishment of the microbiome, and the development of rumen immunity and growth [8, 9].

A detailed and systematic understanding of the molecular genetic mechanisms underlying early rumen development (rumen functions, microbial colonization and their functional interactions) will help in accelerating research towards achieving production efficiency and improvement of ruminants. Previous dynamic molecular studies throughout birth to adult age have characterized the gene expression differences [8], microbial diversity and taxonomic abundance of the rumen [10, 11]. However, most of these studies have focused on either rumen transcription [8] or rumen microbial community composition [10, 12] without information of microbial metabolic functions. To our knowledge, only few studies have been examined on both these two facets during the rumen development, which mainly focused on limited stages (wk 1, 3, or 6) [13].

To fill this gap and to explore the gene expression patterns, microbial community and succession during early development, we collected rumen wall tissues transcriptomic and microbial metagenomic data from rumen of 21 goats at seven different time points (d 1, d 7, d 14, d 21, d 28, d 42, and d 56). We furthermore explored the functional correlations between the rumen and its microbiomes. This study provides a clearer understanding of rumen functional development, microbial colonization and their functional interactions in young ruminants that occur during the establishment from non-ruminant to ruminant, which may provide a mean to manipulate this process in the future to improve efficiency and productivity of ruminants.

## Results

The growth performance phenotypes including rumen anatomical features, the pH, fermentation parameter and morphological development of the 21 goats were determined previously [14, 15]. From day 0 to day 56, rumen pH fluctuated at a range of 5.35 ~ 6.28. The papilla height of the dorsal sac rumen increased rapidly from day 28 [14]. The ratio of rumen net weight to full stomach net weight increased from 29.34% at d 0 to 52.53% at d 56 [15]. Taken together, the 28-day-old was a critical point of gastrointestinal tissue morphological development and its development extent was close to that of 28–56 days [14, 15].

### Dynamic gene expression during early rumen development

**Fig. 1:**
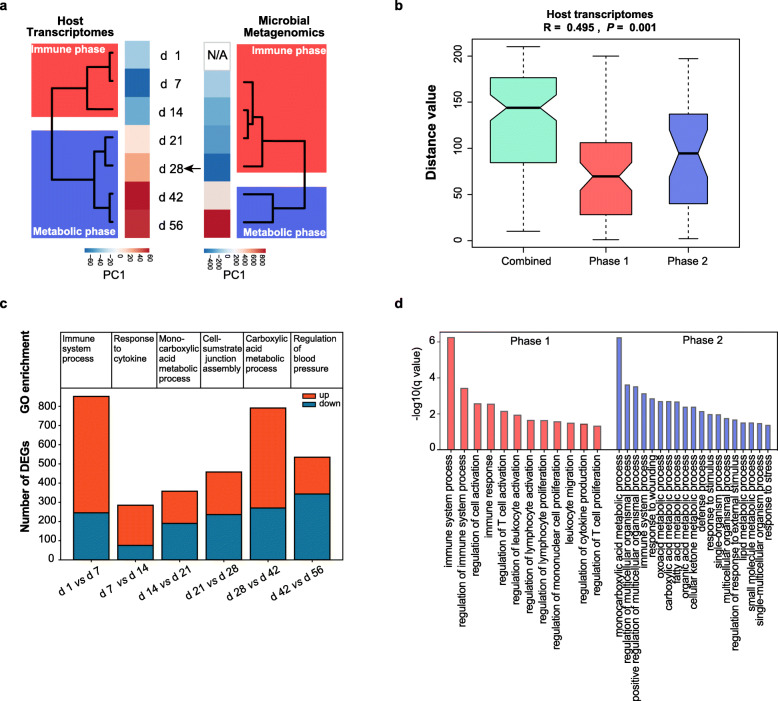
Developmental dynamics revealed by the rumen transcriptome. **a** From the host rumen transcriptome, two stages were identified that transition earlier than the microbial gene abundance of metagenomics by PC1 of PCA analysis and unsupervised hierarchical clustering analysis. The black arrow represented the granulated feed has been added at d 25. **b** ANOSIM test of rumen transcriptomes between the two phases. If the median line of the “combined” group (including data for samples collected during both Phase 1 and Phase 2) was higher than the median lines for the two separate phases, the grouping was considered reasonable, and if the *R* value was greater than 0, there was a significant difference between the groups. In contrast, an *R* value less than 0 indicated that the difference within groups was greater than that between groups. **c** Barplots showing the numbers of upregulated DEGs (colored in orange) and downregulated DEGs (colored in blue) between every two time-points. **d** GO analysis of rumen DEGs during the first phase (colored in orange) and the second phase (colored in blue). Only terms with Bonferroni-adjusted *P* < 0.05 were considered

In the current study, we collected rumen tissues from d 1 to d 56 and sequenced their transcriptomes to reveal the gene expression patterns during early rumen development. From the 21 rumen wall tissue samples, 1,010,353,294 raw reads (~ 151.53 Gbp) were obtained with an average of 48,112,062 ± 5,053,215 raw reads per sample (7.21 ± 0.76 Gbp, Additional file 1: Table S1). After quality control, 954,703,530 clean reads (~ 126.61 Gbp) with an average of 45,462,073 ± 5,090,014 reads per sample (6.03 ± 0.73 Gbp, Additional file 1: Table S1) were retained for further analysis. Both principal component analysis (PCA) and unsupervised hierarchical clustering analysis revealed that the gene transcriptional profiles were divided into two phases: the first phase (d 1–14) and the second phase (d 21–56) (Fig. 1a; Additional file 1: Fig. S1). The analysis of similarities (ANOSIM) also supported the significant differences between the two phases (*P* = 0.001) (Fig. 1b). Furthermore, we identified 2084 differentially expressed genes (DEGs) between each pair of time-points in total (Fig. 1c). Gene ontology (GO) enrichment analysis of DEGs between d 1 vs. d 7 was enriched in the immune system process (adjusted *P* = 4.86 × 10^− 3^, GeneRatio [genes involved in the specific enriched GO term / total differentially expressed genes] = 55/852, Additional file 1: Table S2). DEGs between d 7 vs. d 14 were enriched in response to cytokine (adjusted *P* = 2.93 × 10^− 5^, GeneRatio = 20/285, Additional file 1: Table S3). However, DEGs between d 14 vs. d 21 were enriched in the monocarboxylic acid metabolic process (adjusted *P* = 2.04 × 10^− 3^, GeneRatio = 15/358, Additional file 1: Table S4) and lipid metabolic process (adjusted *P* = 4.31 × 10^− 4^, GeneRatio = 28/358,) as well as in the immune system process (adjusted *P* = 2.11 × 10^− 5^, GeneRatio = 33/358). And DEGs between d 21 vs. d 28 were involved in cell-substrate junction (adjusted *P* = 2.34 × 10^− 5^, GeneRatio = 24/458, Additional file 1: Table S5). DEGs between d 28 vs. d 42 were involved in the carboxylic acid metabolic process (adjusted *P* = 9.48 × 10^− 3^, GeneRatio = 32/791, Additional file 1: Table S6) and for d 42 vs. d 56 we found some enriched categories related to the regulation of blood pressure (adjusted *P* = 2.67 × 10^− 2^, GeneRatio = 11/535, Additional file 1: Table S7). The functional enrichment analyses also indicated two phases during early rumen development. GO and Kyoto Encyclopedia of Genes and Genomes (KEGG) pathway analysis of the rumen DEGs between two mentioned phases also showed that functional enrichment was transformed from involving intense immune responses to enhanced nutrient metabolism-related biological process (Fig. 1d; Additional file 1: Table S8–11). Meanwhile, KEGG enrichment analysis also showed that the rumen DEGs were involved in vascular smooth muscle contraction pathway in the second phase (Additional file 1: Table S11). We thus refer to the first and second stages as the immune and metabolic phases, respectively.

**Fig. 2:**
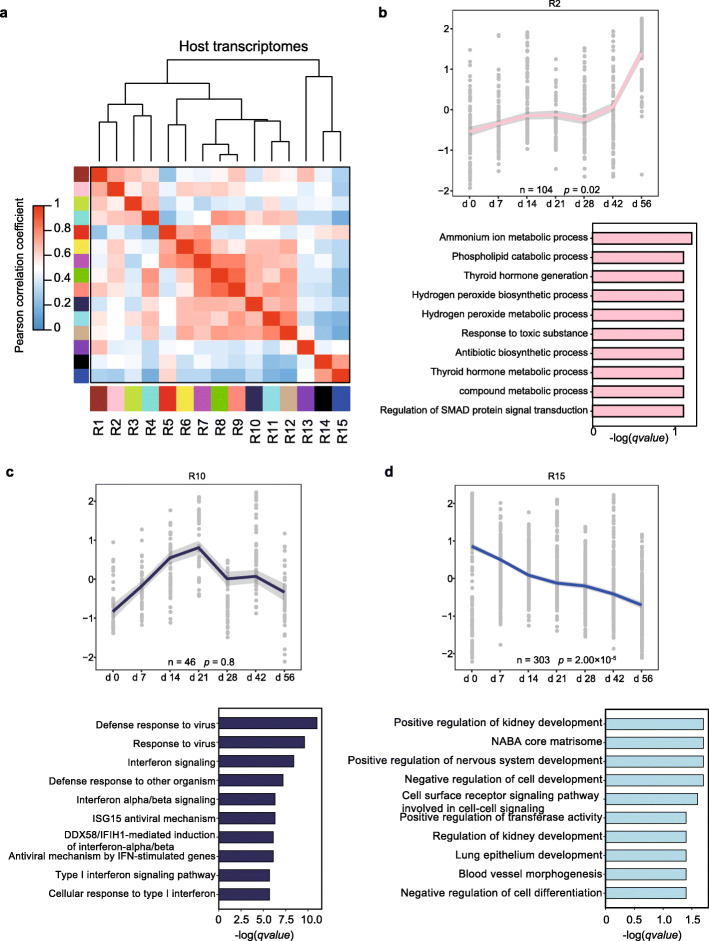
WGCNA of postnatal rumen development transcriptomes. **a** Relationship of gene modules among gene expression of DEGs between each pair of time-points (modules are named by colors). **b** Gene expression modules (R2) showed up-regulation during the early development of the rumen. The barplots indicate the enriched gene ontology terms. **c** Gene expression modules (R10) showed increasing during the immune phase and decreasing during the metabolic phase. **d** Gene expression modules (R15) showed declining during the early rumen development. In each module, n indicates the number of genes in the module. The expression regression line was generated using the Loess curve-fitting method, and *P* values indicate the significance of Spearman’s rank correlation coefficient between the eigengene and age. The eigengene is a central gene whose expression pattern can represent the whole module

To provide a clearer picture of gene expression dynamic patterns and gene interaction relationships in the rumen, a weighted gene co-expression network analysis (WGCNA) was performed on the identified DEGs above. We identified 15 gene expression modules (defined as R1-R15 host modules) that exhibited distinct temporal expression patterns with aging and biological process (Fig. 2a; Additional file 1: Table S12). The age-related up-regulation of these pathways included monocarboxylic acid metabolic, cornification, and ammonium ion metabolic (R2, R4, and R8, respectively; Fig. 2b), which were intercorrelated. We also identified two modules (R10 and R12) that were involved in defense response to virus and cell division, respectively, which exhibited increase with time in the immune phase and then showed declining trend in the metabolic phase (Fig. 2c; Additional file 1: Figs. S2 and 3). And genes in module R15 that were involved in positive regulation of nervous system development and response to peptide, showed declining trends following the early rumen development (Fig. 2d; Additional file 1: Figs. S2 and 3).

### Microbial communities and function successions during early rumen development

In this study, to further reveal the microbial (prokaryotic) colonization patterns during early rumen development, metagenomic samples from the corresponding rumen contents were collected and sequenced from d 1 to d 56 (average of 5.62 Gbp per sample) (Additional file 1: Table S13). However, the metagenomic data at d 1 was contaminated by the massive host DNA (99.80%), then samples of d 1 were excluded in the further analysis. We then obtained 923,801,897 raw reads (~ 129.05 Gbp) from the 17 rumen metagenome samples, with an average of 54,341,288 ± 4,951,973 raw reads per sample (7.59 ± 0.69 Gbp, Additional file 1: Table S13). After quality control, 776,364,309 clean reads (~ 89.04 Gbp) with an average of 45,668,489 ± 5,258,962 reads per sample (5.24 ± 0.82 Gbp) were retained for further analysis. After de novo assembly, we generated 639,641 contigs (> 200 bp in length), with an average of 37,626 ± 11,799 contigs (N50 length, 2775 ± 1444 bp, Additional file 1: Table S14) and 30,692 ± 10,095 scaffolds per sample (N50 length, 6735 ± 4108 bp, Additional file 1: Table S15). Subsequently, our gene prediction results showed a total of 1,320,084 non-redundant genes with an average length of 723 bp for open reading frame (ORF) per each sample (Additional file 1: Table S16). Out of these genes that were derived from the rumen microbiota, 62.9, 59.4 and 38.3% were classified into the NR, EggNOG and KEGG Orthology (KO) database, respectively.

Based on these classifications, a total of 57 bacterial phyla and 1 archaeal phylum were identified, accounting for an average of 77.00% of the reads in the current study. At the phylum level, *Bacteroidetes* (60.24 ± 11.51%) were detected as predominant bacteria, followed by *Firmicutes* (29.09 ± 10.07%), *Proteobacteria* (4.07 ± 3.36%), *Spirochaetes* (1.28 ± 1.20%), *Fusobacteria* (0.75 ± 1.51%) and *Actinobacteria* (0.56 ± 0.24%) (Additional file 1: Fig. S4). Moreover, the archaeal phylum *Euryarchaeota* also accounted for 0.11 ± 0.07% of the reads. Besides, the relative decrease was gradual in *Proteobacteria*, and the decrease of *Fusobacteria* was significant from d 7 to d 14.

At the genus level, a total of 635 genera were identified. And the *Prevotella* (24.36 ± 20.48%), *Bacteroides* (18.2 ± 10.63%), *Porphyromonas* (3.78 ± 5.11%), *Clostridium* (3.63 ± 1.99%), *Ruminococcus* (2.69 ± 1.77%) and *Oscillibacter* (2.05 ± 1.71%) were the most highly presented genera among the early rumen development (Additional file 1: Fig. S5). Also, it should be noted that the high proportion of standard errors should be due to the developmental dynamics of time points and heterogeneity among samples induced by the limited sample size within each time point.

According to the KEGG functional categories, a total of 269 KEGG pathways were observed in our study, which belonged to six level-1 KEGG functional categories, including “metabolism” (59.40 ± 0.02%), “organismal systems” (1.63 ± 0.15%), “human diseases” (0.70 ± 0.05%), “environmental information processing” (10.28 ± 1.22%), “genetic information processing” (25.27 ± 1.12%) and “cellular processes” (2.72 ± 0.38%). At the third level of KEGG functions, “ko02000: transporters (5.41 ± 0.97%)”, “ko03400: DNA repair and recombination proteins” (5.11 ± 0.34%) and “ko00051: Fructose and mannose metabolism” (3.84 ± 0.71%) were abundant during the early rumen development. Moreover, the pathway “ko00500: Starch and sucrose metabolism” (2.37 ± 0.52%) was the most abundant at d 42 (Additional file 1: Table. S17).

**Fig. 3:**
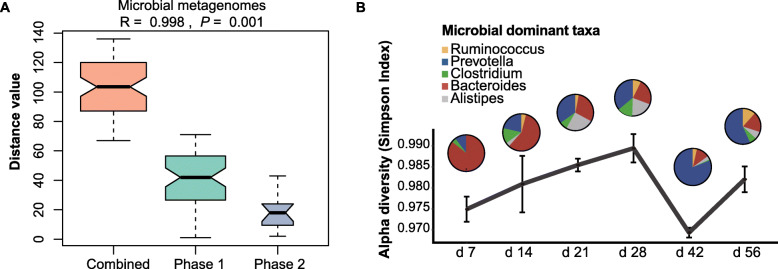
Developmental dynamics revealed by the rumen metagenomes. **a** ANOSIM test of microbial metagenomes between the two phases. If the median line of the “combined” group (including data for samples collected during both Phase 1 and Phase 2) was higher than the median lines for the two separate phases, the grouping was considered reasonable, and if the *R* value was greater than 0, there was a significant difference between the groups. In contrast, an *R* value less than 0 indicated that the difference within groups was greater than that between groups. **b** Pie charts showing the proportions and abundances of the five most abundant microbial genera during the dynamic development of the rumen. The line presents the detailed mean alpha-diversity by Simpson index [16] at the species level, and error bars represent SEM

In this study, differences in microbial community composition were estimated using PCA and unsupervised hierarchical clustering analysis. Similarly, two distinct microbial establishment phases were discerned by PCA and unsupervised hierarchical clustering analysis of the gene abundance profile: the first phase prevailing was before d 28 and the second one was between d 28 and 56 (Fig. 1a and Additional file 1: Fig. S6). And the significant differences between the two phases of microbiomes were also supported by the ANOSIM analysis (*P* = 0.001) (Fig. 3a). The microbial diversity index showed a peak at the transition from one phase to the other (Fig. 3b). The rumen microbial composition and relative abundances at genus level, were also shifted dramatically from being dominated by *Bacteroides* spp. and *Oscillibacter* spp. during d 7 ~ d 28 to *Selenomonas* spp., *Prevotella* spp. and *Ruminococcus* spp. during d 42 ~ d 56 (Fig. 3b). KEGG and KO abundance analyses showed that the bacteriocin biosynthesis was decreased following the transition to the second phase (adjusted *P* = 3 × 10^− 2^), while glycolysis/gluconeogenesis activities and oxidative phosphorylation were increased (adjusted *P* = 3.50 × 10^− 6^ and *P* = 5.93 × 10^− 6^, respectively) (Additional file 1: Table S18). Importantly, fatty acid metabolism and oxidative phosphorylation for supplying energy in the rumen were also accordingly upregulated during the second phase (adjusted *P* = 2.73 × 10^− 5^, for both) (Additional file 1: Table S18).

**Fig. 4:**
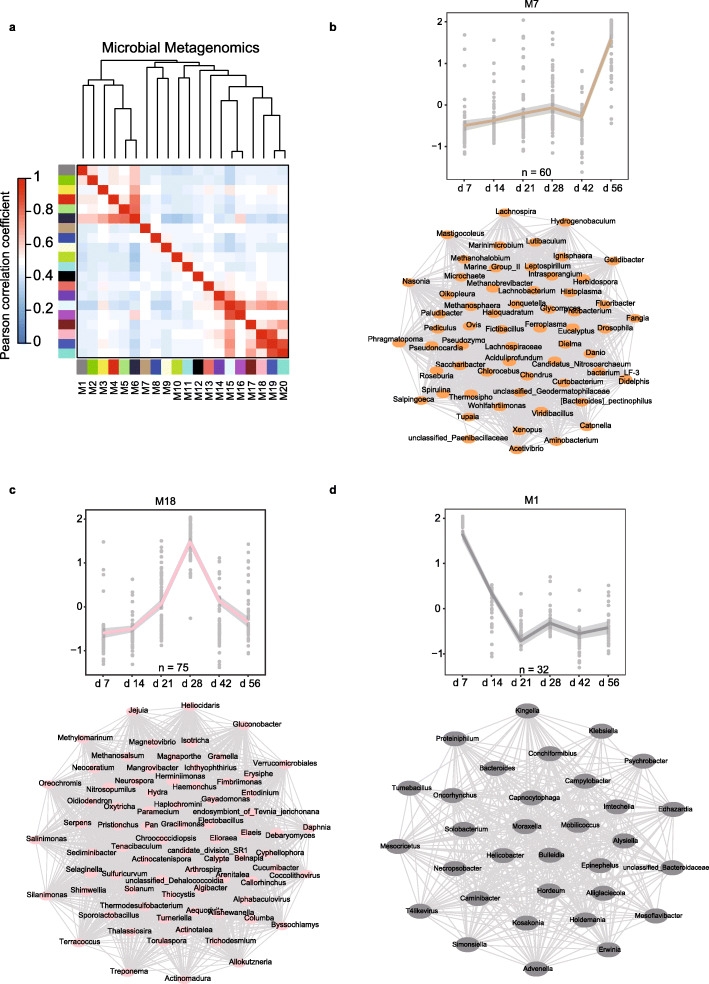
WGCNA of postnatal rumen development metagenomes. **a** Relationship of modules among microbial abundance at genus level (modules are named by colors). **b** Rumen microbial modules at the genus level (M7) showed increasing during early rumen development. **c** Rumen microbial modules at the genus level (M18) showed increasing in immune phase and decreasing in metabolic phase during early rumen development. **d** Rumen microbial modules at the genus level (M1) showed declining in immune phase during early rumen development. In each module, the expression regression line was generated using the Loess curve-fitting method. The eigengene is a central rumen microbial genus whose dynamic abundance pattern can represent the whole module. The networks indicate the correlation between genera by calculating the Pearson correlation coefficient

Furthermore, we performed WGCNA analysis to explore the dynamic patterns of the rumen microbial taxonomic abundance at genus level. We identified 20 modules (defined as M1-M20 microbial modules) that exhibited distinct temporal dynamic patterns (Fig. 4a-d; Additional file 1: Fig. S7; Additional file 2: Table S19). The abundances of *Ruminococcus*, *Bifidobacterium*, *Streptococcus*, *Bacillus*, *Aminobacterium*, and *Methyloceanibacter* showed an increasing tendency in microbial modules M7, M8, and M12 (Fig. 4b; Additional file 1: Fig. S8). In modules M18-M20, the abundances of *Coprobacillus* and *Spirochaeta* showed an increase in the immune phase and then a decrease in metabolic phase (Fig. 4c; Additional file 2: Table S19). Interestingly, rumen fluid contains relatively high numbers of spirochetes of fermenting polymers such as xylan, pectin, and arabinogalactan, which serves as fermentable substrates for the spirochetes, whereas cellulose did not [17]. Therefore, this result confirmed the increasing cellulose in rumen is accompanied by the decreased abundance of non-cellulosic bacteria in the second phase.

### Correlation between the host transcriptome and its microbiomes

To explore the potential host-microbiota interactions, we then calculated the Spearman’s correlation coefficient between the first eigenvector from the PCA of each rumen gene expression module and each microbial genus module, which were identified above. The result of unsupervised hierarchical clustering analysis found three clusters depend on their association patterns (Additional file 1: Fig. S9). Among of these three clusters, the first one consisting of *Lacticigenium*, *Bacteroides*, *Actinobacillus*, *Ruminiclostridium*, *Klebsiella*, and *Propionibacterium* was positively correlated with the expression of the host modules (R5 (*n* = 136), R13 (*n* = 68) and R15 (*n* = 303)) involved in the regulation of cell adhesion, epidermis development, and PPAR signaling pathway (Additional file 1: Figs. S2 and 3; Additional file 2: Table S19). The second cluster contains genera mainly from *Methanoculleus*, *Fibrobacter*, *Actinospica*, and *Prevotella*, that was negatively correlated with the expression of the genes in host modules R6 (*n* = 247), R7 (*n* = 73) and R14 (*n* = 113), which involved in T-cell activation, cellular response to lipid and leukocyte degranulation, respectively (Additional file 1: Figs. S2 and 3; Additional file 2: Table S19).

**Fig. 5:**
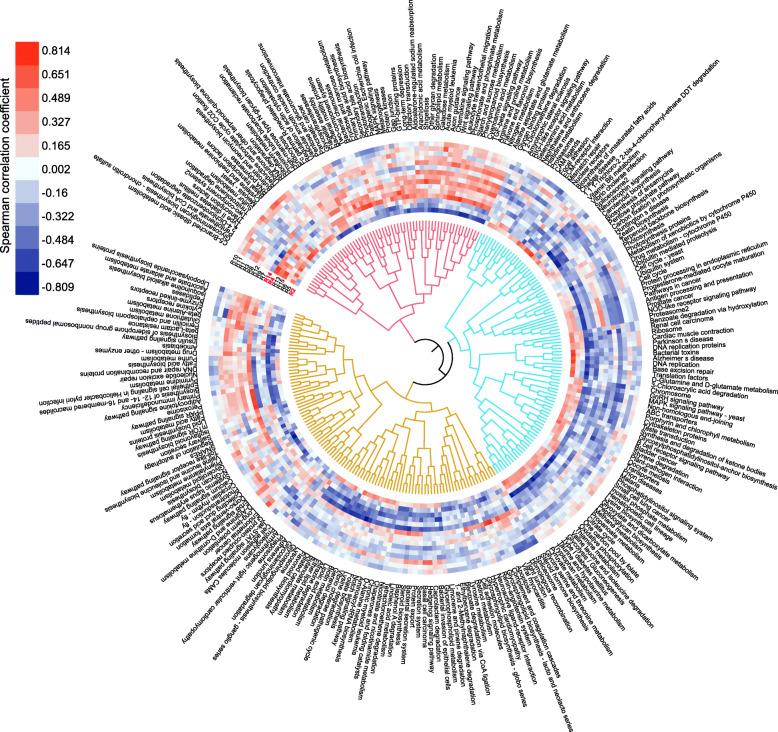
Correlations of host modules with microbial KEGG pathways. Heatmap shows the Spearman correlation coefficient between each rumen transcriptome module and each microbial KEGG pathway (*P* < 0.05). Rumen gene modules obtained using WGCNA and eigengene/PC1 value of each gene module is correlated with the microbial KEGG pathway abundance. The host modules of R2, R4, and R14 are colored in red

To dissect the functional correlations between the rumen and its microbiota, we then evaluated the Spearman’s correlation coefficient between the first eigenvector from the PCA of each host module and the abundance of the microbiota KEGG pathway at the same time point during rumen development from d 7 to d 56 (Fig. 5). We found that genes in the R4 module (*n* = 417) enriched in urogenital system development were positively correlated with microbial glycosaminoglycan biosynthesis and bacterial chemotaxis, and negatively correlated with microbial lysine degradation and folate biosynthesis pathway (Fig. 4). Also, genes in R2 module (*n* = 104) enriched in ammonium ion metabolic process were positively correlated with microbial glycolysis/gluconeogenesis, microbial pentose phosphate pathway and vitamin B6 metabolism (Fig. 5), and negatively correlated with pentose and glucuronate interconversions, microbial nitrogen metabolism and cyanoamino acid metabolism. The results from enrichment analysis of module R14 revealed 113 genes were enriched in IL17 signaling and regulation of innate immune response pathways (Additional file 1: Fig. S2), and showed a persistently high expression pattern during rumen development. The expression of genes in this module was positively correlated with the KEGG pathway abundance of microbes enriched in the penicillin and cephalosporin biosynthesis pathways, which act as the antibiotic competition among microbiota within the rumen ecosystem. Meanwhile, the Spearman’s correlation coefficient between the microbial diversity index and the first eigenvector from the PCA of each host module was calculated (Additional file 1: Table S20). The microbial diversity index was significantly positively correlated with genes in module R13 (*P* value < 0.05), which was related to “PPAR signaling pathway” (adjusted *P* value = 1.26 × 10^− 7^), and negatively correlated with modules R7-R9 that involved in “immunoglobulin production in mucosal tissue” (adjusted *P* value = 5.41 × 10^− 4^), “response to interferon-gamma” (adjusted *P* value = 3.88 × 10^− 7^) and “pyruvate transport” (adjusted *P* value = 8.33 × 10^− 4^) pathways (Additional file 1: Fig. S3).

## Discussion

This study collectively revealed insights into the functional development and interactions that occurred between the rumen and its microorganisms during pre-weaned rumen development. Both gene expression and microbial abundance profiles of the rumen were divided into two stages. Most interestingly, the shift of transcriptional profiles between d 14 and d 21 in rumen was earlier than that of the microbiome (d 28 - d 42) and granule diet introduction (d 25), suggesting that the first stage of rumen development most likely underwent programmed process rather than stimulating effects by feed and microbiome. Meanwhile, we found that the step-by-step establishment of a strong immune capacity in the rumen occurs prior to a shift in functional focus toward nutrient metabolism. The shift is supported by previous findings that lambs first went through a non-ruminant stage (0–3 wk) before entering to the ruminant transition stage (3–8 wk) [5–7]. Furthermore, we found the molecular evidence for an increase in the ruminating capacity during d 21 - d 56, in this time the gene expression levels of the rumen vascular smooth muscle contraction were enhanced.

It is well known that the diet [18, 19] and the timing for feed introducing [20, 21] both have dominant impacts on the rumen microbial colonization process including microbial species and types of metabolism. The rumen microbial composition and relative abundances shifted from being dominated by proteolytic bacteria to amylolytic bacteria and cellulolytic bacteria during the phase transition, which was mostly responsible for the gradual intake of granule and alfalfa after d 25. Moreover, we noticed that the alpha diversity index is generally showed an increasing trend overall, but a transient decrease at d 42, which is a transition time point in the microbial colonization process. Subsequently, the alpha diversity index recovered at d 56. In the first phase (d 1 - d 28), multiple microorganisms introduced into the rumen may result in the increased microbial community diversity. The rumen microbiota of the initial stage was heterogeneous with diverse functions which could meet the requirements in subsequent phase. In the second phase (d 42 - d 56), the diversity and function of rumen microbiota were altered under the enhanced regulation of rumen immune function and the introduction of granulated feed. The results from temporal dynamics of microbial diversity index confirmed previous studies that claimed there were more heterogeneous microbiota in gut microbiome at birth time and then incline to develop into a mature community [10, 11]. The correlation between microbial diversity and host immune response, at least partially, indicated that the host regulation on microbiota was under relatively loose regulation during the immune stage and then became a more tightly regulated pattern. In addition, it has been proposed that the microbiota at birth plays an important role in the development and education of the microbial colonization and host immune system [22]. Previous study had shown that *Bacteroidetes*, *Proteobacteria* and *Actinobacteria* were predominant bacterial community at birth [13]. These bacterial community were similar with the predominant microbial community of d 7 and d 14 (Additional file 1: Fig. S5). The microbiota at birth in rumen may act as a foundation in the subsequently development process of rumen microbiota. Thus, well-designed future studies with larger sample size and post-sucked sampling of d 1 are necessary for in-depth understanding of the microbial colonization during pre-weaned rumen development. Taken together, the first development phase of the rumen was more likely a programmed process rather than effects from inducing by diet and microbiome, while the shift of rumen microbiota was regulated by both the diet and host.

A functional synergetic development was observed between rumen and microbiome following by the colonization of the rumen microorganisms. During the early rumen development stage, the temporal dynamic gene expression patterns were clustered into 15 gene co-expressed modules, which mainly involved in the biological process such as pH homeostasis, nutrients transportation, and antimicrobial. In the R2 module, the high-level expression of *CA1* gene was important for maintaining pH homeostasis in the rumen due to its capacity in hydrating CO2 to bicarbonate [23]. Among all of the 15 gene modules, genes in the modules R2 and R4 which are involved in “Ammonium ion metabolic process”, “Monocarboxylic acid metabolic process”, “Butanoate metabolism” and “Urogenital system development” categories, showed an increasing expression level trend, that was positively correlated with the rumen microbial glycolysis/gluconeogenesis metabolism. Meanwhile, among those genes in the R4 module, *SLC14A1* is responsible for the transportation of endogenous urea from blood to the ruminal lumen in the urea recycling, which provide an additional nitrogen source in the rumen for the synthesis of bacterial protein [24]. As the rumen microorganisms need nitrogen sources for building up their cell mass and reproduction, the increasing ability of nitrogen metabolism in the rumen may be accounted for the co-development between the rumen and its microbiomes to meet the growth requirements of microbiomes. In addition, the expression of genes involved in immune response and antimicrobial was highly increased throughout the early rumen development, that was positively correlated with microbial functions in bacteriocin biosynthesis. These results suggest that bacteriocin production by the rumen microbiota may plays a key role in the rumen immune response and subsequent colonization of microbiota. Noteworthy, in module R2, three genes *DUOX1*, *DUOX2*, and *DUOXA2* had increased expression trend during the early rumen development, which negatively correlated with microbial starch and sucrose metabolism. These findings supported the importance of these genes in the rumen immune system and also in controlling microbial colonization [25]. Also, some genes in the rumen module R14 (*CEBPB*, *S100A9*, *CCL20*, *CXCL8*, *TNF*, *NFKBIA*, *LBP*, *PGLYRP2*, *MUC1*, *SOCS1*, *SOCS3*, and *CCDC3*) were enriched in both IL17 signaling and regulation of innate immune response pathways. Among of these genes, *CEBPB* acts as an important transcription factor regulating genes involved in innate immunity [26], which was most highly expressed at d 1. Although this study lacked the rumen metagenomic data at d 1, it has been reported that rumen microbial colonization begins as early as the first day of life [10]. These findings suggest the host immune regulation of microorganisms has already initiated when the rumen was colonized by microbiomes. In addition, *SOCS1*, *SOCS3*, and *CCDC3* genes are involved in negative regulation of cytokine signaling [27, 28] and TNF-alpha-induced pro-inflammatory response [29] that showed highly expressed at d 1 and then a declining trend during early rumen development (Additional file 1: Fig. S6). These findings show that the host innate immune system has been activated after the colonization of microbiome at d 1. Moreover, the *S100A9* gene, which is related to the antifungal and antibacterial activities [30], was highly expressed throughout the postnatal rumen development stages. This suggests that the host has a continuous regulation on its microbiota to regulate both the access and colonization of microbiomes.

## Conclusions

Taken together, the results of this study revealed the molecular mechanism underlying the temporal dynamics of rumen gene expression patterns and microbiome metagenomes and their relationships during early rumen development in goats. The findings from two different developmental phases and through the programmed process of phase shift in the rumen may help to identify a potential strategy for the selective manipulation of ruminal functions via changes in feeding management to facilitate microbiota changes. The temporal dynamic patterns and coexpressed gene modules and their correlation with the rumen microbiome observed in this study may help to establish a foundation for further research investigating the interactions between the host and microbiota. In future studies, it may also be interesting to more fully elucidate the relationships between the host and microbiome using metatranscriptomics and metabolomics in the rumen.

## Methods

### Animals and sample collection

All animals handling protocols used in this study have been described previously [11, 31]. We described the experimental procedures and approaches here briefly. We used 21 healthy Shaanbei Cashmere goat in this experiment, which was conducted at the Shaanbei Cashmere Goat Original Breeding Farm (Hengshan, Shaanxi, China). Estrus synchronization technology was used to ensure all kids were birthed at the same or adjacent day. All the kids are female, singleton and half siblings. After birth, all selected kids were separated from their mothers and randomly divided into seven age groups (1, 7, 14, 21, 28, 42, and 56 days, *n* = 3 for each group), according to the sampling time point. For the 1 day-age group, newborn kids were euthanized immediately before suckling. In other groups, all kids were housed together with their mothers in the same pen and solely allowed to access to their own dams’ colostrum (0–3 days) or raw milk until d 25. The kids were allowed access to granule and high-quality alfalfa from d 25 (Additional file 1: Table S21) in addition to breast milk. All the kids were fed two equal portions of the diets at 8:00 and 18:00 and were provided with fresh water for ad libitum consumption throughout the entire experiment. All kids were weaned at d 56. By the respective deadline, the kids of each age group were sampled before weaning and without prior feeding. When feeding ewes, the kids and ewes were separated and the kids were fed the solid diets, but they did not touch the ewes’ food. After ewe feeding, the feeders were removed, then the kids were released from the feeding fence. By the respective deadline, the kids of each age group were sacrificed without prior feeding. All goats were anaesthetized via intravenous injection of thiopental (0.125 mg/kg of body weight), which was purchased from Kangjiano Biological Co., Ltd., Suzhou, China) and then were euthanized via intravenous injection of potassium chloride (5-10 mL), which was purchased from Sinopharm Chemical Reagent Co., Ltd., Shanghai, China, following the procedures of previous studies [11, 32, 33]. The whole rumen of each of these kids were collected as a closed section to prevent environmental contamination. After rinsed with sterile PBS, samples of the rumen wall tissue (~ 2 cm^2^ in size) from each of kids were collected at the bottom of the ventral sac quickly, and the site of sampling was the same for all animals. Tissue samples were snap-frozen in liquid nitrogen and stored at − 80 °C for subsequent total RNA analysis. The ruminal contents were placed and homogenized into 20 ml cryopreservation tubes and snap-frozen in liquid nitrogen and stored at − 80 °C for further DNA analysis.

### RNA isolation, library construction, and sequencing

Total RNA was isolated from all tissue samples, according to the Trizol protocol (Invitrogen, Carlsbad, CA, USA). A total amount of 1.5 μg high-quality RNA per sample was used as input material for the RNA sample preparations. Sequencing libraries were generated using a NEBNext® Ultra RNA Library Prep Kit for Illumina® (NEB, Beverly, MA, USA), according to the manufacturer’s recommendations. And index codes were added to attribute sequences to each sample. The library preparations were sequenced on an Illumina Hiseq X Ten platform, and 150 bp paired-end reads were generated.

### RNA-seq data quality control and quantification processing

High-quality reads were obtained by removing adaptor sequences and filtering low-quality reads from raw reads using Trimmomatic (version 0.36) [34] with the following parameters: LEADING:3 TRAILING:3 SLIDINGWINDOW:4:15 MINLEN:40. High-quality reads from 21 samples of goats were aligned to the NCBI assembly goat reference genome (GCF_001704415.1). For this, we used STAR (Version 2.5.1) [35] with the following parameters: outFilterMultimapNmax 1 and outFilterMismatchNmax 10. Unmapped reads were extracted by SAMtools (Version 1.3) [36] for further mapping by HISAT2 (Version 2.0.3-beta) [37] for more efficient use of data. We normalized the read depth by computing Fragments Per Kilobase per Million mapped reads (FPKM) values for the transcripts and genes in each sample using StringTie (Version1.3.4) [38] and Ballgown (Version 2.2.0) [39].

### Identification of temporal differentially expressed genes

We used Cuffdiff (Version 2.2.1) [40] with “time-series” parameters to identify genes that were temporally expressed. A standard threshold (false-discovery-rate q value of < 0.05 and log2-transformed FPKM difference ≥ 2-fold) was used to identify the DEGs.

### Gene ontology (GO) and Kyoto Encyclopedia of Genes and Genomes (KEGG) enrichment analysis

GO functional enrichments analysis were performed by using Fisher’s exact test to determine the significance of enrichment of components based on their hypergeometric distribution using an in-house script. *P* values were corrected for multiple testing using the Bonferroni method implemented in the R *p.adjust* package, and a final corrected *P* < 0.05 was considered significant. KEGG functional enrichment was assessed using KOBAS 3.0 [41, 42] (http://kobas.cbi.pku.edu.cn/) by Fisher’s exact test following with Bonferroni method for multiple testing.

### DNA extraction, library construction, and metagenomic sequencing

For metagenomics analysis, total genomic DNA was extracted from rumen content samples by a bead-beating method (RBB + C). This method is based on a mini-bead beater (Biospec Products, Bartlesville, USA) using an E.Z.N.A.® Universal DNA Kit (Omega Bio-tek, Norcross, GA, USA) according to the manufacturer’s protocols. The integrity and quantity of genomic DNA were examined by electrophoresis in 1% agarose gels, a TBS-380 mini-fluorometer (Turner BioSystems, California, USA), and a NanoDrop2000 spectrophotometer (Thermo Scientific, Grand Island, NY, USA). One milligram of genomic DNA from each sample was then fragmented to obtain sequences of approximately 300 bp, using Covaris M220 (Gene Company Limited, China) for paired-end library construction, and with a TruSeqTM DNA Sample Prep Kit (Illumina, San Diego, CA, USA). Adapters containing the full complement of sequencing primer hybridization sites were ligated to the blunt-end fragments. All samples were then sequenced using an Illumina HiSeq X Ten platform (Illumina Inc., San Diego, CA, USA) and a 150-bp PE strategy using a HiSeq 3000/4000 PE Cluster Kit and HiSeq 3000/4000 SBS Kits according to the manufacturer’s instructions (www.illumina.com). Note that due to the DNA quality of one sample at d 7 cannot meet the requirements for sequencing, there are two biological replicates at d 7. Thus, the final metagenomic sample size was 17.

### Metagenomics quality control

The obtained raw sequence reads were first trimmed with Trimmomatic (version 0.36) [34] to remove adapter sequences and filtering low-quality reads. Leading or trailing stretches of Ns and bases with quality < 25 were also trimmed, and reads were scanned with a 4-base wide sliding window and cut when the average quality per base dropped below 25. Only reads of at least 40 nucleotides in length were kept. Additionally, according to one previous analysis by BWA-MEM [36] (version 0.7.15, with default parameters except “-M” enabled), all reads that were aligned to the NCBI assembly goat reference genome (GCF_001704415.1), were also removed. Finally, on average, 11% of the clean reads corresponding to the host (goat) genome DNA were removed. The remaining set of high-quality reads was used for further analysis.

### De novo assembly and gene calling

In order to construct a comprehensive catalog of genes from rumen development microbial, high-quality reads were used for de novo assembly with SOAPdenovo software (v2.20) [43], with the following parameters: -d 1, −R, −K 39 for tuning to maximize the contig N50 value. The contigs were assembled into scaffolds with the parameters -L 200. The assembly was broken at N connections to obtain scafftigs for gap filling, which was performed using the intrinsic gap-filling function of SOAPdenovo and Gapcloser v1.12 (a companion program released with SOAPdenovo) softwares. The high-quality reads from each sample were assembled separately and mapped again to the scaffolds by SoapAligner software (v2.20) [44] to acquire the PE reads which were not used for assembly with the following parameters: identity ≥90%, −m 200, −u, − 2. Unassembled reads from all samples were co-assembled in a final global assembly to identify rare genes with the same parameters that were used for the single-sample assemblies. Scafftigs shorter than 500 bp generated from single samples or co-assemblies were removed before further analysis.

ORFs in the scafftigs (≥ 500 bp) were predicted with MetaGeneMark (version 3.26) [45] from the predicted results with default parameters for prokaryote. A non-redundant gene catalogue was constructed with CD-HIT (version 4.6) [46], using a sequence identity cut-off of 0.95, with a minimum coverage of 0.9 for shorter sequences and grouping shorter genes. We then using the NCBI Genetic Codes, translated the ORFs of the predicted non-redundant gene set into protein sequences (https://www.ncbi.nlm.nih.gov/Taxonomy/taxonomyhome.html/index.cgi?chapter=tgencodes#SG1) with an in-house script.

To assess the abundance of gene transcripts, reads were aligned to the gene catalogue with SoapAligner [44] using the following parameters: -m 200, −× 400, identity ≥95%. Genes with ≤2 reads in each sample were then filtered [47, 48], and the remaining genes (Unigenes) were used for subsequent analysis.

### Quantification of metagenome content

The abundance of a gene in a sample was normalized by dividing the number of reads that were uniquely mapped to that gene by the gene length. After that, normalized gene abundances were transformed into relative abundance by dividing them by the total number of uniquely mapped reads for a given sample. We calculated the abundances following the method [49–53] using an in-house script. The resulting set of gene relative abundance was called as individual’s microbial gene profile, which were used for further analyses.

### Taxonomic annotation and functional annotation

The taxonomic assignment of predicted genes was carried out by DIAMOND software (Version 0.8.24.86) [54]. Multiple sequence alignment against sequences from Bacteria, Fungi, Archaea, and Viruses were extracted from the integrated NR database (Version 20,161,014, https://www.ncbi.nlm.nih.gov/). For the deficient of protozoa genomes in NR database, we only focused on the prokaryotes in further analysis. BLASTP alignment was used to filter hits with e-values >1e-5, and for each gene, the significant matches (defined by e-values at least 10-fold higher than that of the top hit) were retained to distinguish taxonomic groups. Then, we determined the taxonomical level of each gene by the lowest common ancestor (LCA)-based algorithm implemented in MEGAN5 [55], which assigns genes to taxa at taxonomic levels reflecting their conservation levels. The numbers of genes and their abundances in each sample at seven taxonomic levels (kingdom, phylum, class, order, family, genus, and species) were obtained from the LCA annotation results and gene abundance tables. The abundance of each taxon in each sample was estimated from the sum of the gene abundances assigned to the taxon, and the number of a taxon’s genes in a sample was estimated from the number of genes with non-zero abundance.

We conducted a KO abundance analysis to construct the KO profiles of the microbiomes to reflect the microbes’ functions. Gene functional annotations were made by DIAMOND (Version 0.8.24.86) [54] with a cutoff of e-value <1e-5 to search against the KEGG databases and map the KO IDs of the best hits for our predicted ORFs (genes); this allowed us to identify KO-annotated genes and determine their matched KO numbers. For the second-level KO profiles, we utilized the annotations for the non-redundant genes and summed the relative abundances of genes assigned to the same KO. For the third-level Ko pathway profiles, we summed the relative abundances of KOs assigned to the same Ko pathway. The resulting gross relative abundances were treated as the KO and Ko contents of each sample and used to generate KO and Ko profiles of the samples, respectively.

### Statistical analysis and alpha diversity analysis

We applied PCA (R prcomp in the gmodels package, Version 2.16.2) to decrease the dimensionality of the gene expression data for rumen transcriptome and abundance data for each taxonomic level. Analysis of similarity (ANOSIM) is a non-parametric test method based on permutation test, which is used to test whether the difference between groups is significantly greater than the difference within groups [56]. A suitable distance dissimilarity matrix is produced by function “vegdist.bray”. The distances between pairs of groups were sorted in descending and converted to ranks. According to the classification of distances, the difference between the mean value of distance rank between groups and the mean value of distance rank within groups was calculated as a statistical magnitude. ANOSIM tests were then used to test for significant differences between groups based on the rumen gene expression profile (d 1 - d 56) and ruminal microorganism gene abundance profile (d 7 - d 56) for rumen transcriptomes and metagenomes, respectively. Pairwise ANOSIMs between all pairs of groups are provided as a post-hoc test. Significant comparisons (at *P* < 0.05) are decided based on step-down sequential Bonferroni. Permutation tests between groups were applied using STAMP (Version 2.1.3) [57] to obtain the probabilities of differences at each taxonomic level, and the Benjamini and Hochberg False Discovery Rate were then applied to correct the *P* values and acquire *q* values. For each analysis, the *P* values were determined based on 1000 permutations. The Simpson index of alpha diversity values was calculated with rumen microbial abundance at the genus level (R package vegan, Version2.4.4). Microbial taxa and functional features with a relative abundance greater than 0.01% in the sum of all samples were retained for analysis. Values in the current study were presented as the mean ± standard error of the mean (SEM) unless otherwise indicated.

### Weighted gene co-expression network analysis

The interactions among the rumen genes and microbial metagenomes were explored through network analysis and correlation analysis. We used 2035 DEGs between each pair of time-points from rumen wall transcriptomes as input for WGCNA to understand the link between the rumen transcriptome and the rumen microbial metagenome. This allowed us to identify the gene co-expression modules with the similar gene expression trend in both rumen gene expression and microbial metagenomes and their functional correlations. Signed weighted gene co-expression network analysis was performed using the WGCNA R package [58]. A power test was performed to determine the soft-threshold using pickSoftThreshold. The optimal soft-threshold power was set to 8 (Additional file 1: Fig. S10). This threshold was further used to compute gene expression distance for module detection. Based on the distance matrix, genes were subsequently clustered using the average linkage hierarchical clustering method using hclust, and the expression modules were detected using dynamicTreeCut. The modules with similar patterns were further clustered and merged into the consensus modules (Additional file 1: Fig. S11). The correlation between the consensus modules and age was calculated using *corPvalueStudent*. Pairwise Pearson correlation coefficients were calculated for all selected genes. The resulting Pearson correlation matrix was transformed into a matrix of connection strengths (an adjacency matrix) using a power function, which was then converted to a topological overlap matrix. WGCNA seeks to identify modules of densely interconnected genes by hierarchical clustering based on topological overlap [59]. Genes not assigned to any modules were excluded from further analysis. The Spearman correlation coefficient between each host module and age (Additional file 1: Table S20) was calculated by an in-house script. Modules with *P* < 0.05 were considered to have a significant correlation with age. For each module, a functional enrichment analysis was carried out using Metascape [60]. Similarly, we also conducted a WGCNA analysis on microbial genus abundance profile. The optimal soft-threshold power was set to 10 (Additional file 1: Fig. S12).

### Interaction between the rumen gene expression with microbial

The Spearman correlation coefficients analysis between the rumen gene module with microbial genus module and microorganism KEGG pathway abundance was calculated by an in-house script. The expression level of a module eigengene is defined as the first principal component of a given module and used to represent the overall expression level of a module. A correlation test *P* value was used to assess the statistical significance between pairs of variables. Modules with *P* < 0.05 were considered to have a significant correlation as described in a previous study [13].

## Supplementary Information

**Additional file 1: Figure S1.** Unsupervised hierarchical clustering analysis showing the relationships among 21 transcriptomic samples in goats and a heatmap showing the pairwise Pearson correlations. **Figure S2.** WGCNA of postnatal rumen development transcriptomes. **Figure S3.** Barplots showing the top 10 GO enrichment terms in each host module. **Figure S4.** Top 20 microbial abundance at the phylum level at each time point. **Figure S5.** Top 20 microbial abundance at the genus level at each time point. **Figure S6.** Unsupervised hierarchical clustering analysis showing the relationships among 17 metagenomic samples in goats and a heatmap showing the pairwise Pearson correlations. **Figure S7.** The clustering tree of 1782 genus on the determination of the optimal soft threshold power for rumen microbiome modules. **Figure S8.** WGCNA of postnatal rumen development metagenomes. **Figure S9.** Correlations of host modules with microbial modules at genus level. **Figure S10.** The tests on the determination of the optimal soft threshold power for rumen host modules. **Figure S11.** The clustering tree of 2035 DEGs on the determination of the optimal soft threshold power for rumen modules. **Figure S12.** The tests on the determination of the optimal soft threshold power for rumen microbiome modules in genus level. **Table S1.** Statistical data for 21 rumen transcriptome samples in goats. **Table S2.** GO enrichment analysis of 852 DEGs between d 7 vs. d 1 during rumen development. **Table S3.** GO enrichment analysis of 285 DEGs between d 14 vs. d 7 during rumen development. **Table S4.** GO enrichment analysis of 358 DEGs between d 21 vs. d 14 during rumen development. **Table S5.** GO enrichment analysis of 458 DEGs between d 28 vs. d 21 during rumen development. **Table S6.** GO enrichment analysis of 791 DEGs between d 42 vs. d 28 during rumen development. **Table S7.** GO enrichment analysis of 535 DEGs between d 56 vs. d 42 during rumen development. **Table S8.** GO enrichment analysis of 1032 DEGs in the first phase during rumen development. **Table S9.** GO enrichment analysis of 1509 DEGs in the second phase during rumen development. **Table S10.** KEGG enrichment results for the DEGs during early rumen development in the first phase. **Table S11.** KEGG enrichment results for the DEGs during early rumen development in the second phase. **Table S12.** Spearman’s correlation coefficient and *P* value of each host module with age. **Table S13.** Statistical data for the metagenomics raw data. **Table S14.** Statistical data for metagenomics assembly (contig-level). **Table S15.** Statistical data for metagenomics assembly (scaffold-level). **Table S16.** Statistical data for non-redundant gene catalog annotation. **Table S17.** The relative abundance of KEGG pathway abundance (Mean ± SEM) at the third level during the early rumen development. **Table S18.** KEGG enrichment results for the rumen metagenome in two phases. **Table S20.** Spearman’s correlation coefficient and *P* value of the first eigenvector from the PCA of each host module with microbial alpha diversity index. **Table S21.** Ingredients and nutrients of the experimental diets.

**Additional file 2: Table S19.** Genera in each microbial module.

## Data Availability

The datasets analyzed in this study are all available in NCBI (BioProject number PRJNA485657). Raw reads for all transcriptomics data from the rumen wall and metagenomics data from the corresponding rumen fluid samples are available in the short-read archive, with accession numbers given in Table S1 and Table S13. The custom scripts and codes have deposited in GitHub (https://github.com/xiangyupan/metagenomics_basic).

## Abbreviations

PCA: Principal component analysis
ANOSIM: Analysis of similarities
DEGs: Differentially expressed genes
GO: Gene ontology
KEGG: Kyoto Encyclopedia of Genes and Genomes
WGCNA: Weighted gene co-expression network analysis
ORF: Open reading frame
KO: KEGG Orthology
FPKM: Fragments Per Kilobase per Million mapped reads
LCA: Lowest common ancestor
SEM: Standard error of the mean
