# Supplementary material for: Dynamics of rumen gene expression, microbiome colonization, and their interplay in goats
Source: BMC Genomics. 2021 Apr 21;22:288. doi: 10.1186/s12864-021-07595-1 (PMC8059226; doi:10.1186/s12864-021-07595-1)
Supplement: Supplementary file 1 — Additional file 1: Figure S1. Unsupervised hierarchical clustering analysis showing the relationships among 21 transcriptomic samples in goats and a heatmap showing the pairwise Pearson correlations. Figure S2. WGCNA of postnatal rumen development transcriptomes. Figure S3. Barplots showing the top 10 GO enrichment terms in each host module. Figure S4. Top 20 microbial abundance at the phylum level at each time point. Figure S5. Top 20 microbial abundance at the genus level at each time point. Figure S6. Unsupervised hierarchical clustering analysis showing the relationships among 17 metagenomic samples in goats and a heatmap showing the pairwise Pearson correlations. Figure S7. The clustering tree of 1782 genus on the determination of the optimal soft threshold power for rumen microbiome modules. Figure S8. WGCNA of postnatal rumen development metagenomes. Figure S9. Correlations of host modules with microbial modules at genus level. Figure S10. The tests on the determination of the optimal soft threshold power for rumen host modules. Figure S11. The clustering tree of 2035 DEGs on the determination of the optimal soft threshold power for rumen modules. Figure S12. The tests on the determination of the optimal soft threshold power for rumen microbiome modules in genus level. Table S1. Statistical data for 21 rumen transcriptome samples in goats. Table S2. GO enrichment analysis of 852 DEGs between d 7 vs. d 1 during rumen development. Table S3. GO enrichment analysis of 285 DEGs between d 14 vs. d 7 during rumen development. Table S4. GO enrichment analysis of 358 DEGs between d 21 vs. d 14 during rumen development. Table S5. GO enrichment analysis of 458 DEGs between d 28 vs. d 21 during rumen development. Table S6. GO enrichment analysis of 791 DEGs between d 42 vs. d 28 during rumen development. Table S7. GO enrichment analysis of 535 DEGs between d 56 vs. d 42 during rumen development. Table S8. GO enrichment analysis of 1032 DEGs in the first phase during rume [file 12864_2021_7595_MOESM1_ESM.docx]

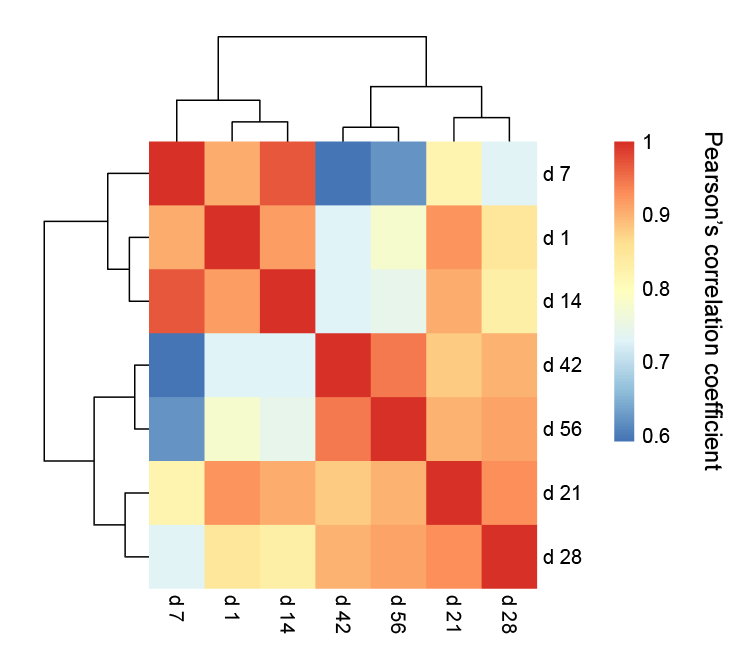


## Fig. S1 Unsupervised hierarchical clustering analysis showing the relationships among 21 transcriptomic samples in goats and a heatmap showing the pairwise Pearson correlations.


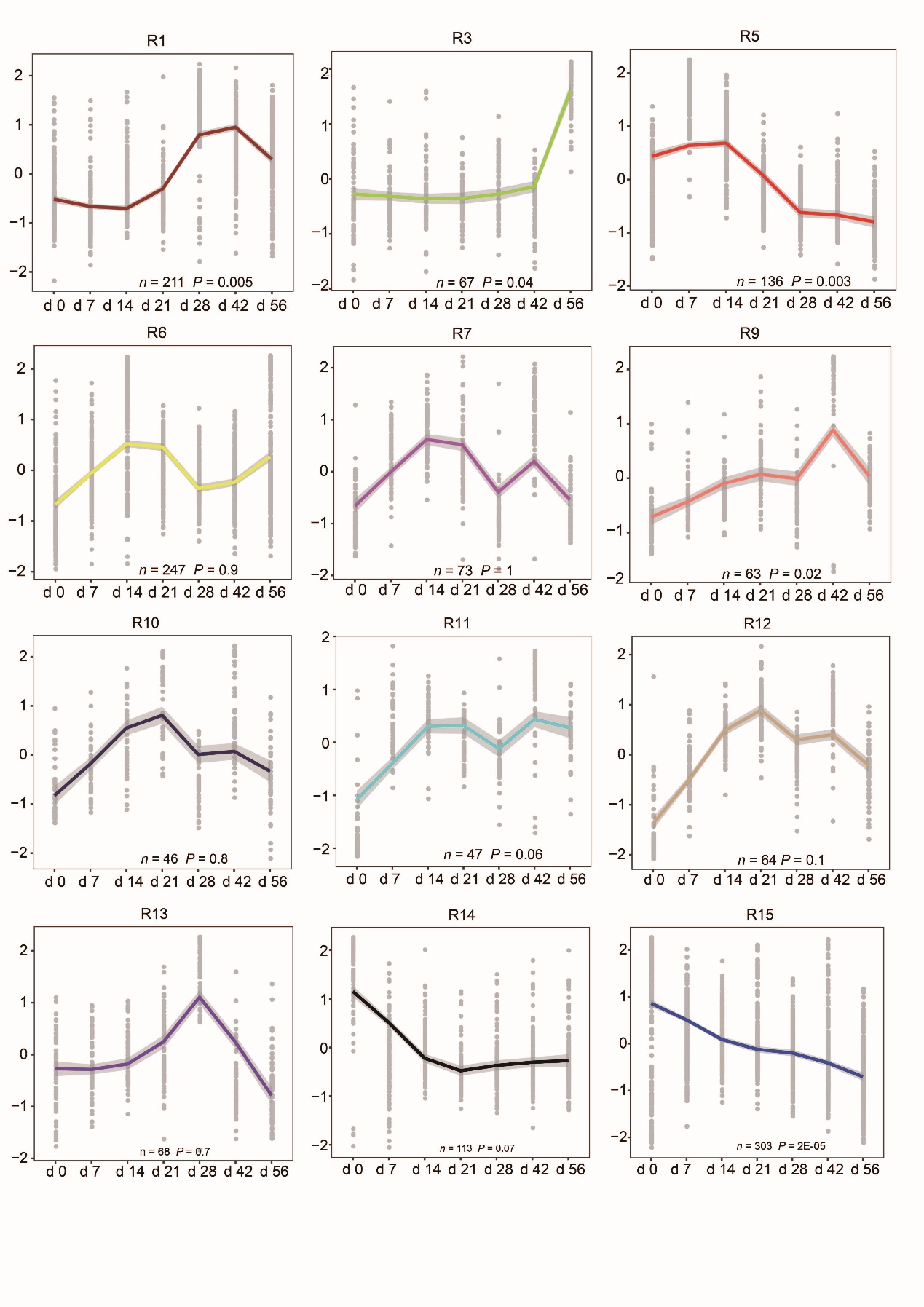


## Fig. S2. Weighted gene co-expression network analysis (WGCNA) of postnatal rumen development transcriptomes. Gene expression modules showed temporal dynamic expression during the development of postnatal rumen. The networks indicate the enriched gene ontology terms. In each module, n indicates the number of genes in the module. The expression regression line was generated using the Loess curve-fitting method, and *P* values indicate the significance of Spearman’s rank correlation coefficient between the eigengene and age. The eigengene is a central gene whose expression pattern can represent the whole module.


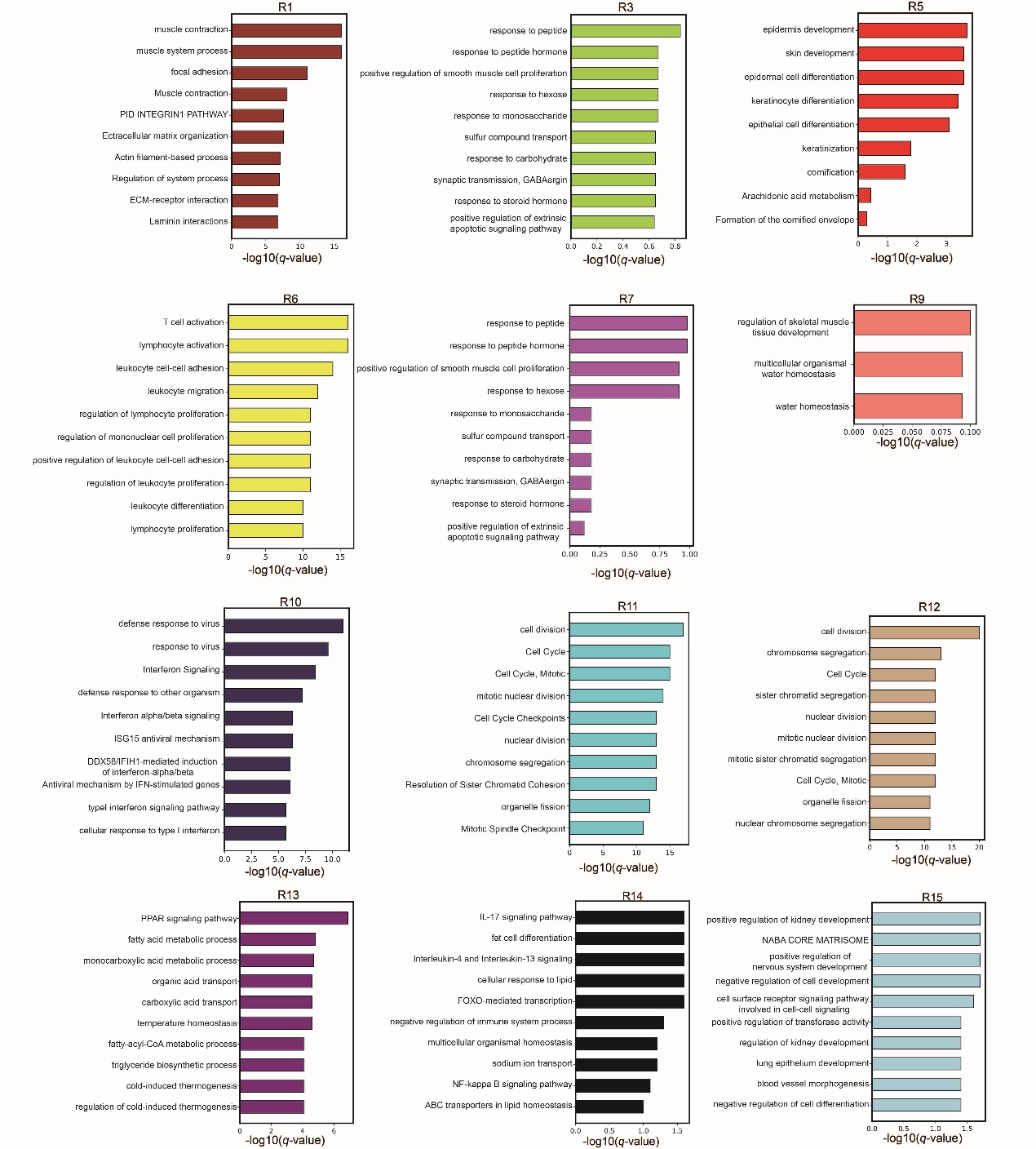


## Fig. S3 Barplots showing the top 10 GO enrichment terms in each host module.


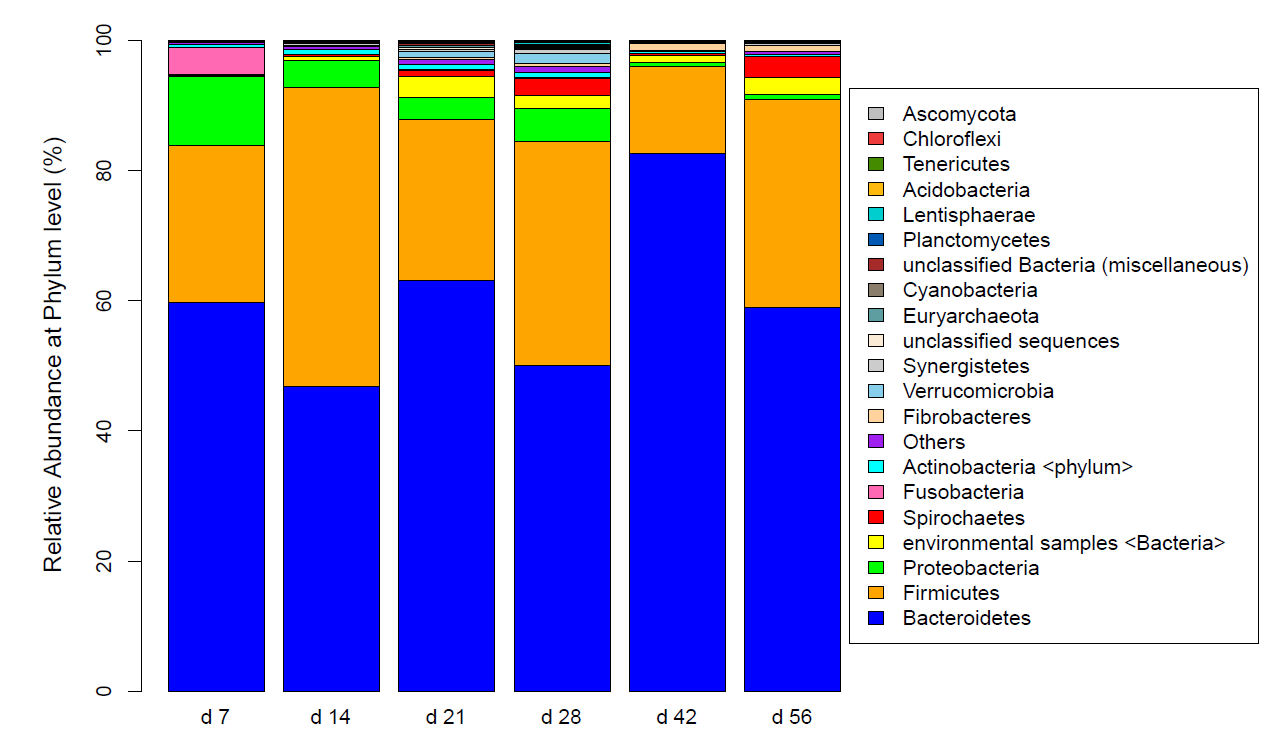


## Fig. S4. Top 20 microbial abundance at the phylum level at each time point.


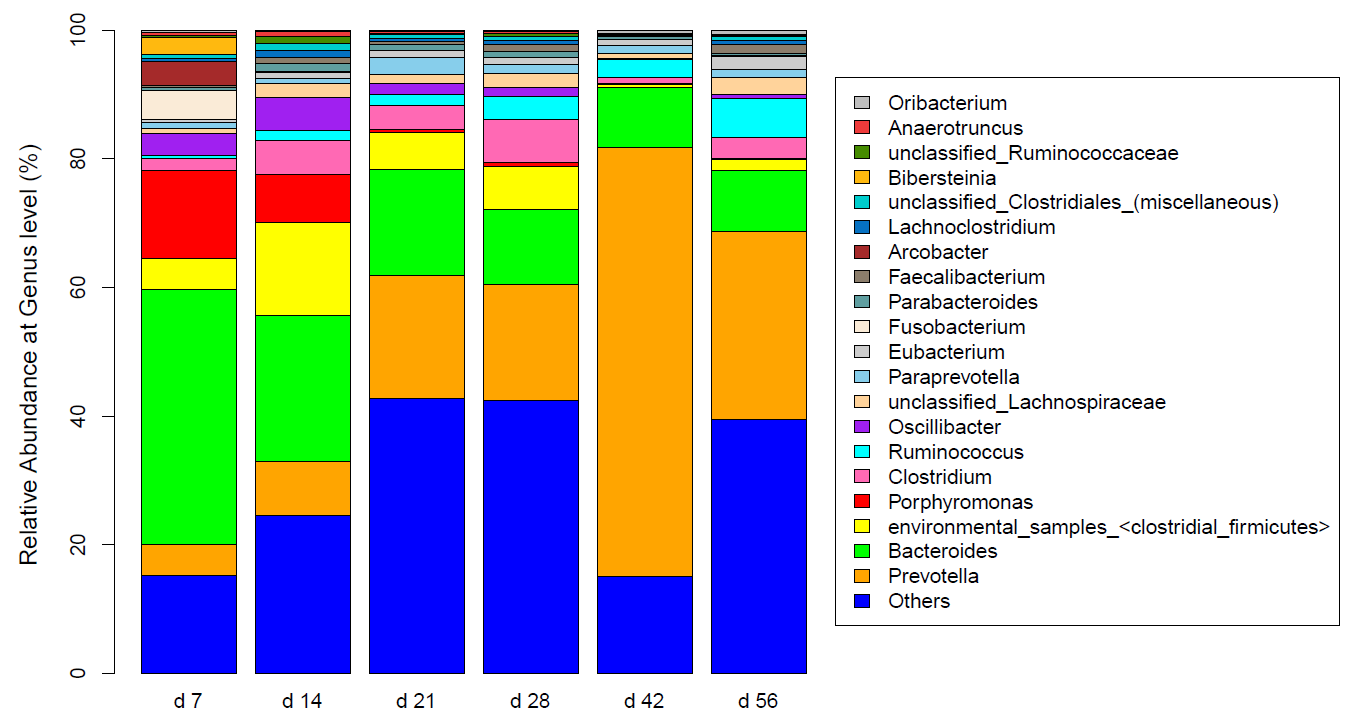


## Fig. S5. Top 20 microbial abundance at the genus level at each time point.


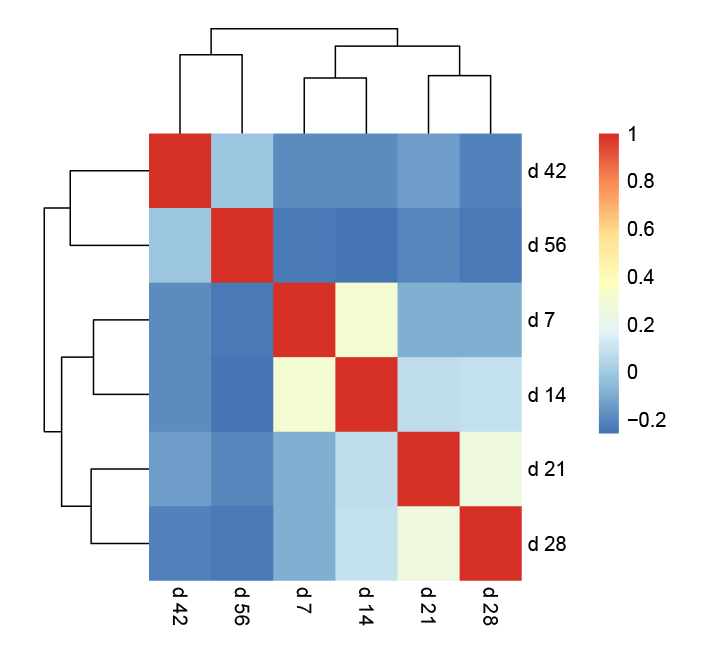


## Fig. S6 Unsupervised hierarchical clustering analysis showing the relationships among 17 metagenomic samples in goats and a heatmap showing the pairwise Pearson correlations.


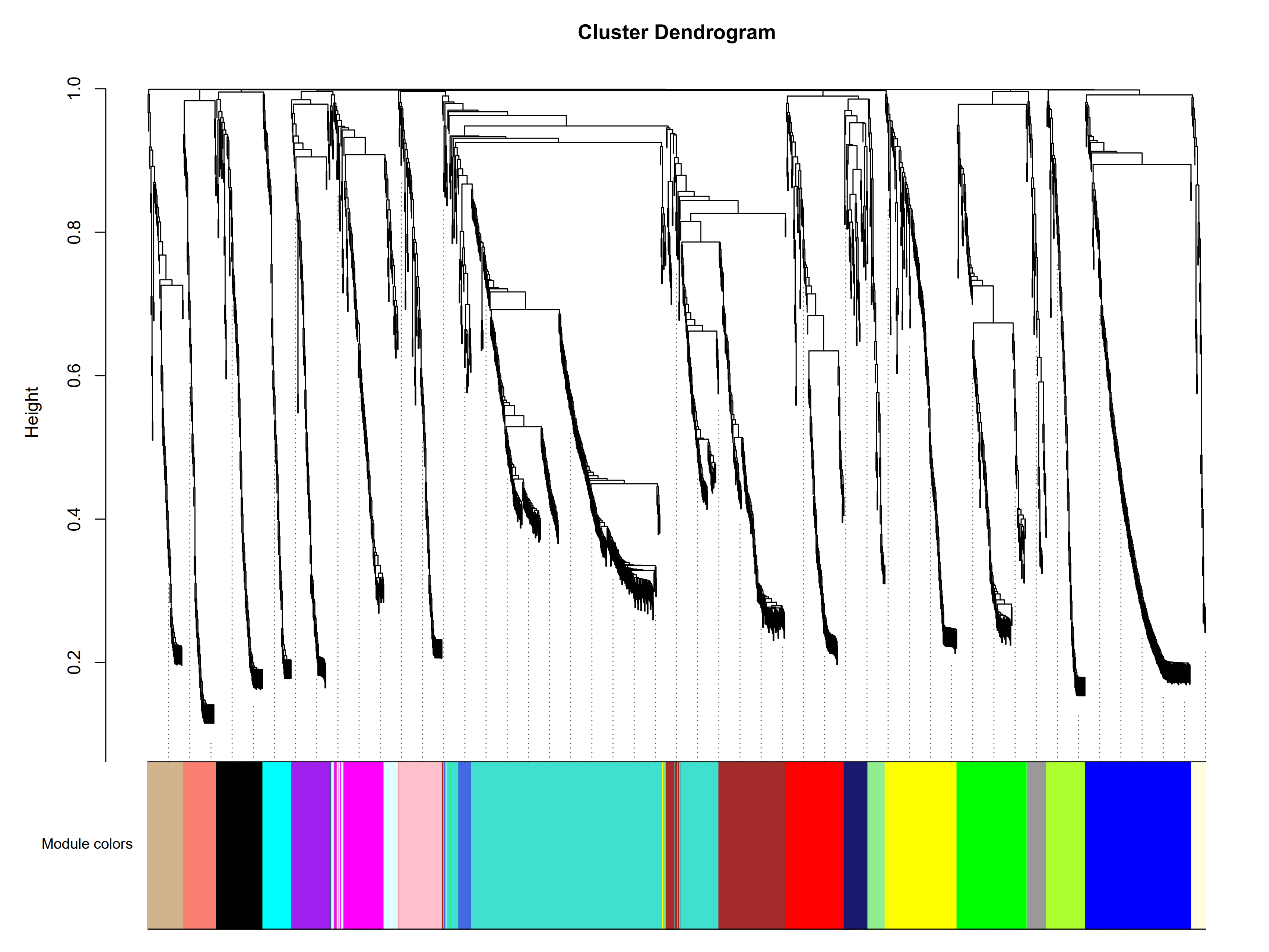


## Fig. S7. The clustering tree of 1,782 genus on the determination of the optimal soft threshold power for rumen microbiome modules. The colors indicate the genus in different expression modules.


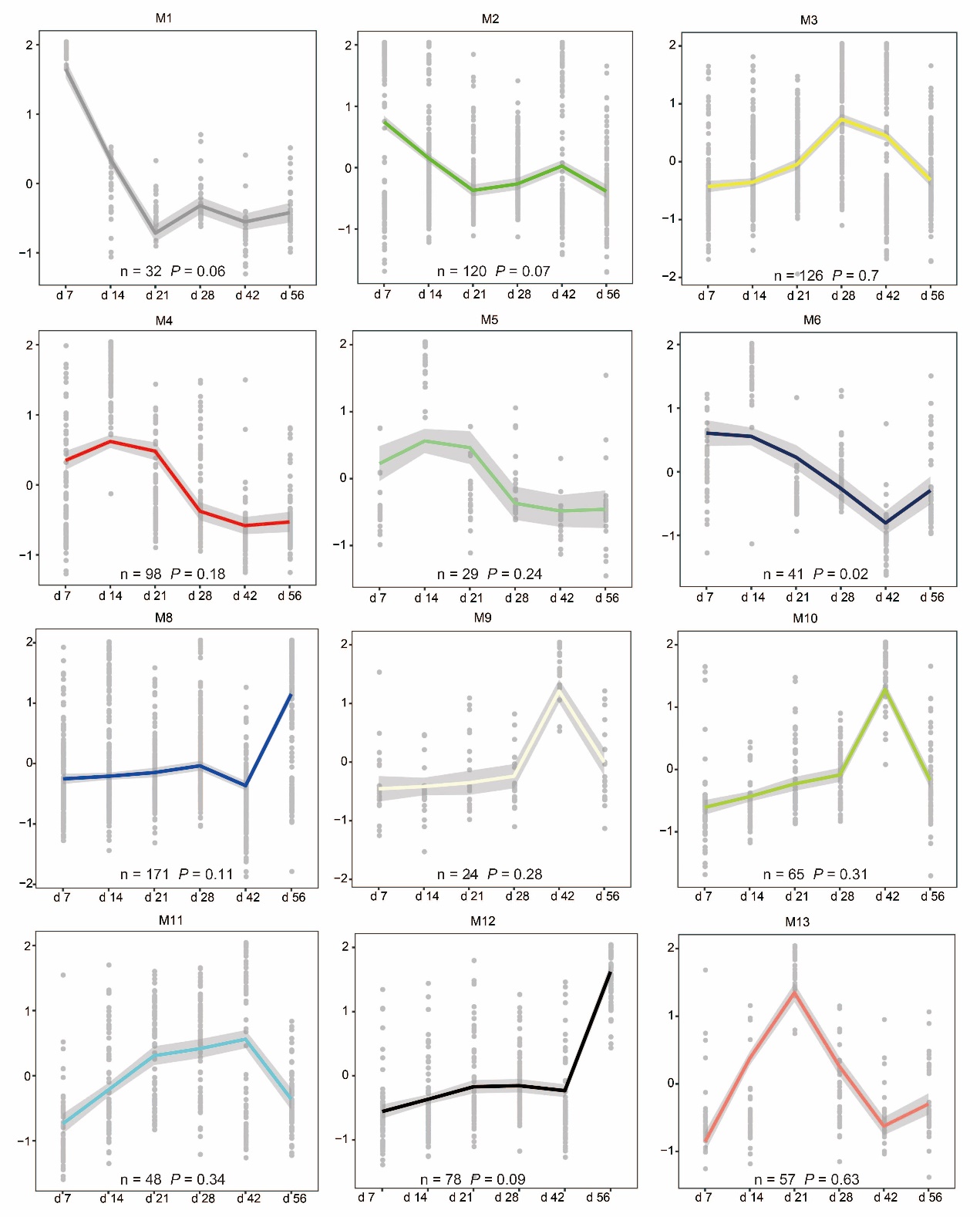

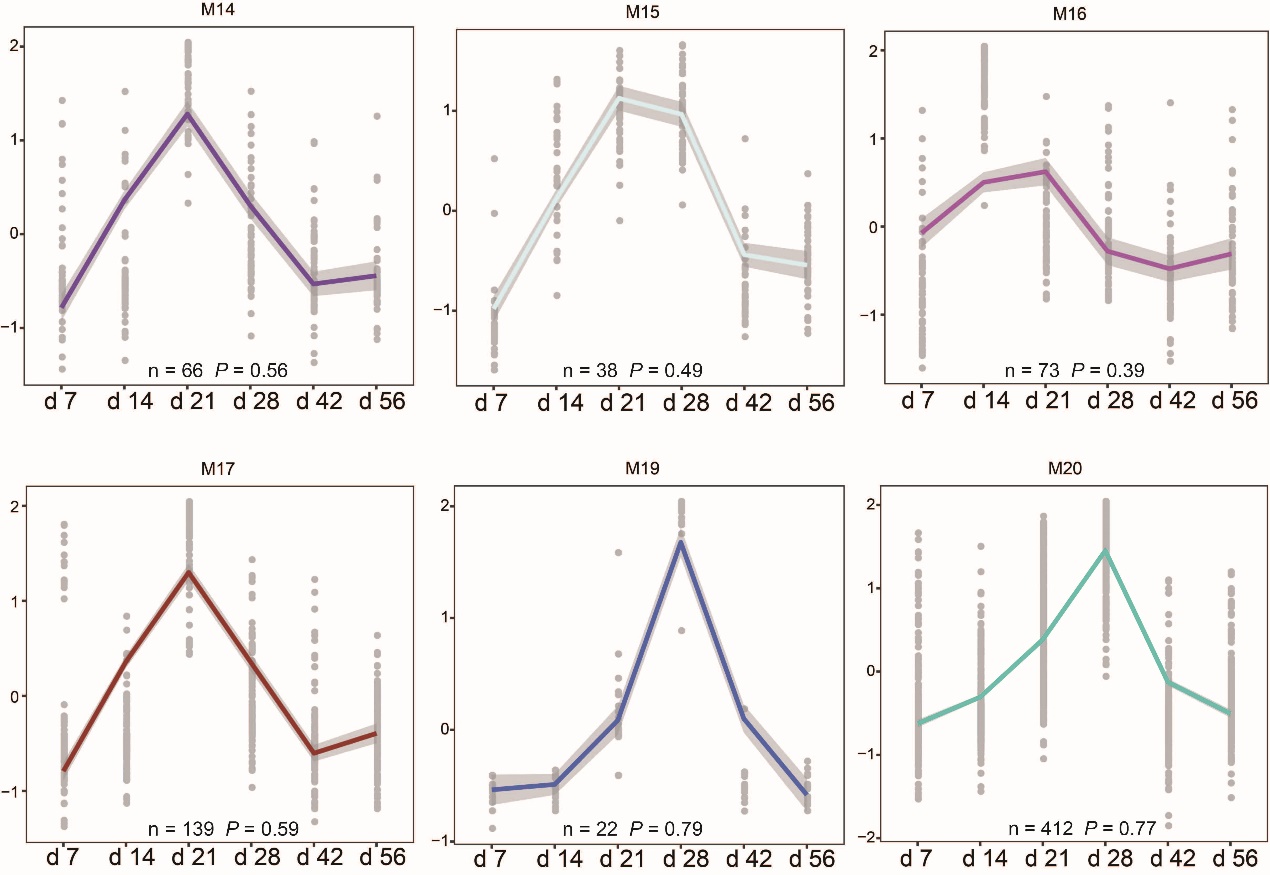


## Fig. S8. Weighted gene co-expression network analysis (WGCNA) of postnatal rumen development metagenomes. Rumen microbial modules at genus level. In each module, the expression regression line was generated using the Loess curve-fitting method. The eigengene is a central rumen microbial genus whose dynamic abundance pattern can represent the whole module.


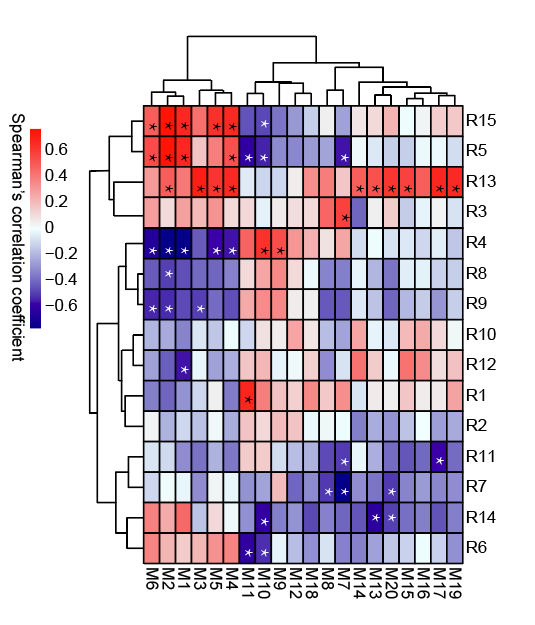


## Fig. S9. Correlations of host modules with microbial modules at genus level. Heatmap showing the Spearman correlation coefficient between the first eigenvector from the PCA of each rumen transcriptome module and each rumen microbial module at genus level. The black asterisk represents positive correlation and the white asterisk represents negative correlation.


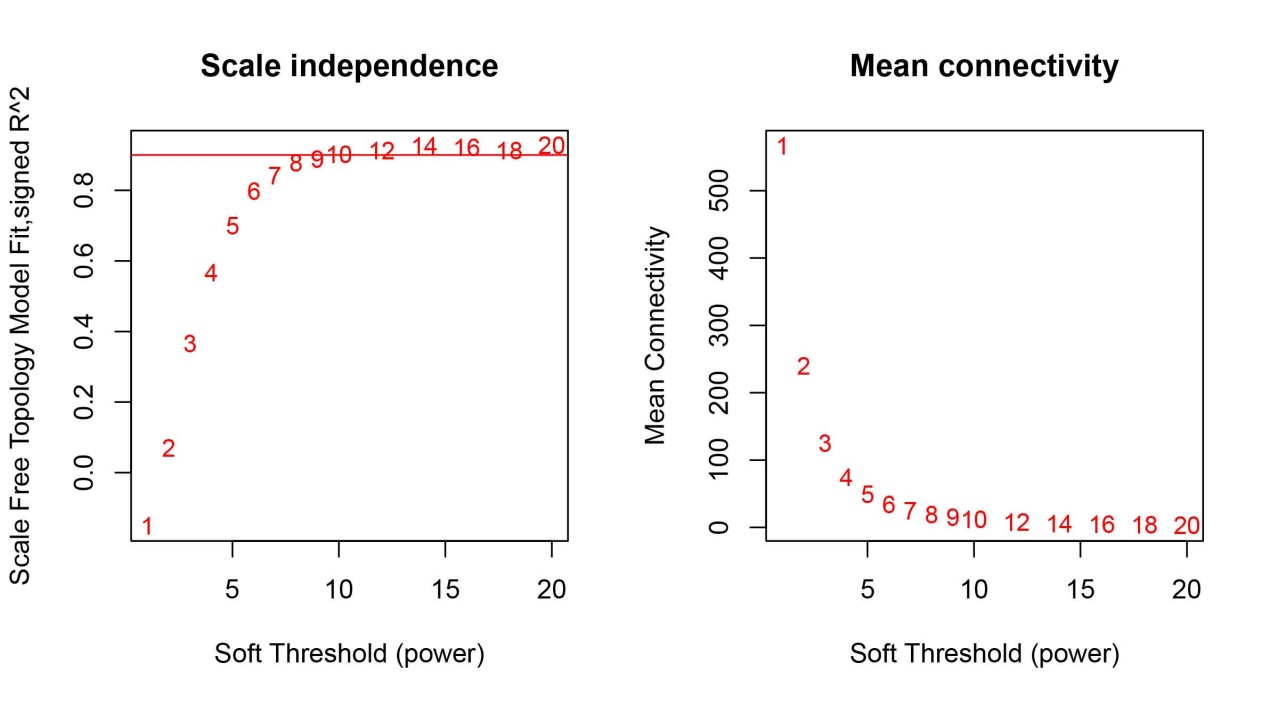


## Fig. S10. The tests on the determination of the optimal soft threshold power for rumen host modules. Based on the scale free topology model fit value and mean connectivity value, 8 was selected as the optimal soft threshold power.


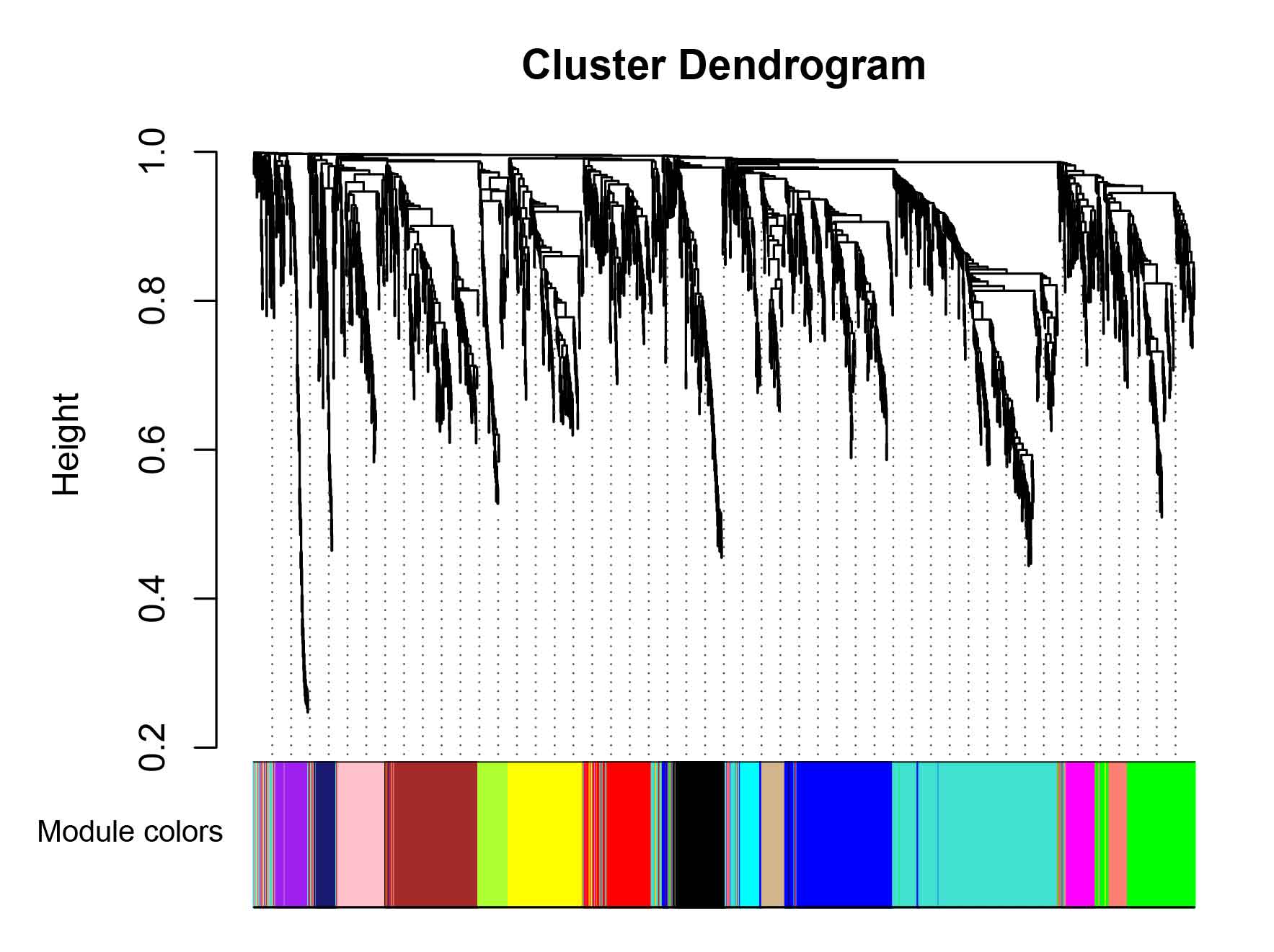


## Fig. S11. The clustering tree of 2,035 DEGs on the determination of the optimal soft threshold power for rumen modules. The colors indicate the genes in different expression modules.


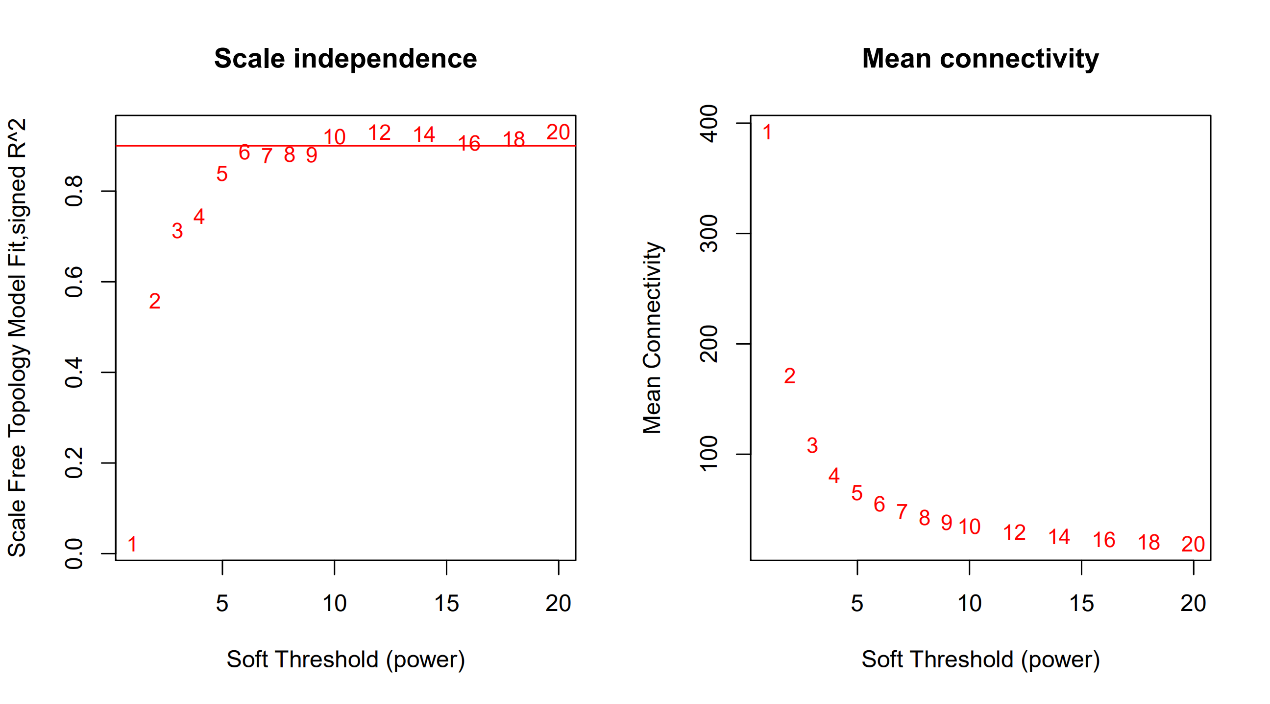


## Fig. S12. The tests on the determination of the optimal soft threshold power for rumen microbiome modules in genus level. Based on the scale free topology model fit value and mean connectivity value, 10 was selected as the optimal soft threshold power.

## Table S1. Statistical data for 21 rumen transcriptome samples in goats.

| **Sample ID** | **Aligned ratio (%)** | **Raw Sequence (bp)** | **Number of raw reads** | **Clean Sequence (bp)** | **Number of clean reads** | **NCBI_ID** |
| --- | --- | --- | --- | --- | --- | --- |
| d 0-1 | 95.21 | 8,059,366,800 | 53,729,112 | 7,408,485,391 | 51,136,326 | SRR7755226 |
| d 0-2 | 96.11 | 7,490,763,000 | 49,938,420 | 7,053,292,478 | 48,342,732 | SRR7755229 |
| d 0-3 | 92.47 | 7,229,715,900 | 48,198,106 | 6,737,921,421 | 46,319,336 | SRR7755228 |
| d 7-1 | 93.16 | 7,114,925,100 | 47,432,834 | 6,656,455,513 | 45,723,372 | SRR7755343 |
| d 7-2 | 94.44 | 5,895,736,200 | 39,304,908 | 5,216,105,745 | 36,938,260 | SRR7755342 |
| d 7-3 | 94.91 | 6,443,587,200 | 42,957,248 | 5,683,311,653 | 40,238,666 | SRR7755222 |
| d 14-1 | 94.94 | 6,250,417,800 | 41,669,452 | 5,563,495,046 | 39,345,260 | SRR7755231 |
| d 14-2 | 95.11 | 6,853,569,300 | 45,690,462 | 6,100,666,742 | 43,402,146 | SRR7755230 |
| d 14-3 | 93.71 | 6,819,967,200 | 45,466,448 | 5,975,367,365 | 42,470,912 | SRR7755233 |
| d 21-1 | 93.39 | 6,823,797,900 | 45,491,986 | 5,865,717,127 | 43,166,412 | SRR7755232 |
| d 21-2 | 94.43 | 6,794,253,600 | 45,295,024 | 5,982,448,697 | 42,450,828 | SRR7755225 |
| d 21-3 | 94.59 | 6,004,837,800 | 40,032,252 | 5,302,627,439 | 37,522,496 | SRR7755224 |
| d 28-1 | 94.84 | 7,903,203,600 | 52,688,024 | 7,015,342,737 | 49,309,020 | SRR7755349 |
| d 28-2 | 94.17 | 7,330,878,900 | 48,872,526 | 6,470,066,685 | 45,580,368 | SRR7755348 |
| d 28-3 | 94.8 | 7,067,435,400 | 47,116,236 | 6,283,209,325 | 44,172,922 | SRR7755351 |
| d 42-1 | 94.82 | 8,093,920,200 | 53,959,468 | 7,224,790,932 | 50,713,244 | SRR7755350 |
| d 42-2 | 92.81 | 7,077,046,200 | 47,180,308 | 6,276,104,505 | 44,163,736 | SRR7755345 |
| d 42-3 | 94.17 | 7,535,035,200 | 50,233,568 | 6,747,366,149 | 47,293,984 | SRR7755344 |
| d 56-1 | 92.9 | 7,740,651,300 | 51,604,342 | 6,893,188,326 | 48,364,700 | SRR7755347 |
| d 56-2 | 89.52 | 9,173,647,800 | 61,157,652 | 8,678,550,866 | 59,970,472 | SRR7755227 |
| d 56-3 | 94.34 | 7,850,237,700 | 52,334,918 | 6,811,496,185 | 48,078,338 | SRR7755346 |
| **Sum** | **-** | **151,552,994,100** | **1,010,353,294** | **135,946,010,327** | **954,703,530** | **-** |
| **Average** | **94.04** | **7,216,809,243** | **48,112,062** | **6,473,619,539** | **45,462,073** | **-** |
| **SD** | **1.344186778** | **757,982,234** | **5,053,215** | **783,057,512** | **5,090,014** | **-** |

| **ID** | **Type** | **Description** | **Fisher's exact test *P* value** | **Adjusted *P* value** | **Input number** | **Background number** |
| --- | --- | --- | --- | --- | --- | --- |
| GO:0000775 | CC | chromosome, centromeric region | 8.53E-07 | 0.014523 | 12 | 49 |
| GO:0002376 | BP | immune system process | 2.85E-07 | 0.004858 | 55 | 619 |
| GO:0005615 | CC | extracellular space | 2.31E-06 | 0.039286 | 57 | 695 |
| GO:0044421 | CC | extracellular region part | 7.89E-09 | 0.000134 | 143 | 2119 |

## Table S2. GO enrichment analysis of 852 DEGs between d 1 *vs*. d 7 during rumen development. BP denotes Biological Process and CC denotes Cellular Component.

## Table S3. GO enrichment analysis of 285 DEGs between d 7 *vs*. d 14 during rumen development. BP denotes Biological Process, MF denotes Molecular Function, and CC denotes Cellular Component.

| **ID** | **Type** | **Description** | **Fisher's exact test *P* value** | **Adjusted *P* value** | **Input number** | **Background number** |
| --- | --- | --- | --- | --- | --- | --- |
| GO:0001817 | BP | regulation of cytokine production | 1.49E-06 | 0.025372 | 16 | 316 |
| GO:0001968 | MF | fibronectin binding | 6.94E-07 | 0.011821 | 5 | 15 |
| GO:0002376 | BP | immune system process | 1.01E-11 | 1.73E-07 | 31 | 619 |
| GO:0002682 | BP | regulation of immune system process | 4.91E-07 | 0.008357 | 23 | 579 |
| GO:0004896 | MF | cytokine receptor activity | 7.93E-07 | 0.013509 | 8 | 62 |
| GO:0005576 | CC | extracellular region | 5.52E-07 | 0.009407 | 22 | 538 |
| GO:0005615 | CC | extracellular space | 6.58E-08 | 0.00112 | 27 | 695 |
| GO:0006952 | BP | defense response | 1.29E-07 | 0.002192 | 19 | 373 |
| GO:0006955 | BP | immune response | 1.20E-07 | 0.00204 | 17 | 297 |
| GO:0009986 | CC | cell surface | 1.63E-11 | 2.77E-07 | 21 | 275 |
| GO:0010033 | BP | response to organic substance | 2.33E-07 | 0.003967 | 32 | 990 |
| GO:0034097 | BP | response to cytokine | 1.72E-09 | 2.93E-05 | 20 | 319 |
| GO:0034341 | BP | response to interferon-gamma | 9.08E-07 | 0.015466 | 6 | 28 |
| GO:0043230 | CC | extracellular organelle | 1.09E-06 | 0.018531 | 41 | 1,574 |
| GO:0044421 | CC | extracellular region part | 5.19E-11 | 8.84E-07 | 59 | 2,119 |
| GO:0050900 | BP | leukocyte migration | 1.08E-07 | 0.001844 | 10 | 88 |
| GO:0065010 | CC | extracellular membrane-bounded organelle | 1.09E-06 | 0.018531 | 41 | 1,574 |
| GO:0070062 | CC | extracellular exosome | 1.07E-06 | 0.018222 | 41 | 1,573 |
| GO:0070098 | BP | chemokine-mediated signaling pathway | 3.91E-07 | 0.006653 | 7 | 39 |
| GO:0071345 | BP | cellular response to cytokine stimulus | 1.54E-06 | 0.026254 | 15 | 279 |

## Table S4. GO enrichment analysis of 358 DEGs between d 14 *vs*. d 21 during rumen development. BP denotes Biological Process, MF denotes Molecular Function, and CC denotes Cellular Component.

| **ID** | **Type** | **Description** | **Fisher's exact test *P* value** | **Adjusted *P* value** | **Input number** | | **Background number** |
| --- | --- | --- | --- | --- | --- | --- | --- |
| GO:0002252 | BP | immune effector process | 1.91E-06 | 0.032557 | 13 | 159 | |
| GO:0002376 | BP | immune system process | 1.24E-09 | 2.11E-05 | 33 | 619 | |
| GO:0006629 | BP | lipid metabolic process | 2.53E-08 | 0.000431 | 28 | 524 | |
| GO:0006952 | BP | defense response | 7.15E-07 | 0.012173 | 21 | 373 | |
| GO:0006955 | BP | immune response | 1.65E-06 | 0.028108 | 18 | 297 | |
| GO:0007159 | BP | leukocyte cell-cell adhesion | 2.38E-06 | 0.040507 | 6 | 25 | |
| GO:0032787 | BP | monocarboxylic acid metabolic process | 1.2E-07 | 0.002039 | 15 | 171 | |
| GO:0044255 | BP | cellular lipid metabolic process | 1.89E-07 | 0.003215 | 23 | 407 | |
| GO:0044281 | BP | small molecule metabolic process | 1.86E-06 | 0.031631 | 29 | 684 | |
| GO:0044421 | CC | extracellular region part | 6.39E-08 | 0.001088 | 65 | 2119 | |
| GO:0050900 | BP | leukocyte migration | 1.73E-07 | 0.002951 | 11 | 88 | |

## Table S5. GO enrichment analysis of 458 DEGs between d 21 *vs*. d 28 during rumen development. BP denotes Biological Process and CC denotes Cellular Component.

| **ID** | **Type** | **Description** | **Fisher's exact test *P* value** | **Adjusted *P* value** | **Input number** | **Background number** |
| --- | --- | --- | --- | --- | --- | --- |
| GO:0005912 | CC | adherens junction | 2.04E-08 | 0.000348 | 25 | 289 |
| GO:0005924 | CC | cell-substrate adherens junction | 1.26E-09 | 2.14E-05 | 24 | 233 |
| GO:0005925 | CC | focal adhesion | 5.47E-09 | 9.32E-05 | 23 | 231 |
| GO:0007044 | BP | cell-substrate junction assembly | 2.28E-07 | 0.00389 | 7 | 20 |
| GO:0030055 | CC | cell-substrate junction | 1.37E-09 | 2.34E-05 | 24 | 234 |
| GO:0043230 | CC | extracellular organelle | 1.40E-06 | 0.023915 | 66 | 1574 |
| GO:0044421 | CC | extracellular region part | 1.47E-08 | 0.00025 | 88 | 2119 |
| GO:0044449 | CC | contractile fiber part | 8.73E-10 | 1.49E-05 | 16 | 97 |
| GO:0065010 | CC | extracellular membrane-bounded organelle | 1.40E-06 | 0.023915 | 66 | 1574 |
| GO:0070062 | CC | extracellular exosome | 1.37E-06 | 0.023379 | 66 | 1573 |
| GO:0070161 | CC | anchoring junction | 4.02E-08 | 0.000684 | 25 | 299 |

## Table S6. GO enrichment analysis of 791 DEGs between d 28 *vs*. d 42 during rumen development. BP denotes Biological Process and CC denotes Cellular Component.

| **ID** | **Type** | **Description** | **Fisher's exact test *P* value** | **Adjusted *P* value** | **Input number** | **Background number** |
| --- | --- | --- | --- | --- | --- | --- |
| GO:0006082 | BP | organic acid metabolic process | ### | 0.015459 | 32 | 322 |
| GO:0019752 | BP | carboxylic acid metabolic process | ### | 0.009481 | 32 | 315 |
| GO:0031224 | CC | intrinsic component of membrane | ### | 0.028426 | 157 | 2934 |
| GO:0032787 | BP | monocarboxylic acid metabolic process | ### | 0.000693 | 24 | 171 |
| GO:0042180 | BP | cellular ketone metabolic process | ### | 0.028284 | 32 | 331 |
| GO:0043436 | BP | oxoacid metabolic process | ### | 0.009481 | 32 | 315 |
| GO:0044281 | BP | small molecule metabolic process | ### | 0.014605 | 53 | 684 |
| GO:0044421 | CC | extracellular region part | ### | 0.020241 | 122 | 2119 |

## Table S7. GO enrichment analysis of 535 DEGs between d 42 *vs*. d 56 during rumen development. BP denotes Biological Process and CC denotes Cellular Component.

| **ID** | **Type** | **Description** | **Fisher's exact test *P* value** | **Adjusted *P* value** | **Input number** | **Background number** |
| --- | --- | --- | --- | --- | --- | --- |
| GO:0005615 | CC | extracellular space | 4.05E-07 | 0.006897 | 43 | 695 |
| GO:0008217 | BP | regulation of blood pressure | 1.57E-06 | 0.026701 | 11 | 66 |
| GO:0031224 | CC | intrinsic component of membrane | 1.36E-06 | 0.02318 | 118 | 2934 |
| GO:0044057 | BP | regulation of system process | 1.17E-06 | 0.019925 | 26 | 328 |

## Table S8. GO enrichment analysis of 1,032 DEGs in the first phase during rumen development. BP denotes Biological Process, MF denotes Molecular Function, and CC denotes Cellular Component.

| **ID** | **Type** | **Description** | **Fisher's exact test *P* value** | **Adjusted *P* value** | **Input number** | **Background number** |
| --- | --- | --- | --- | --- | --- | --- |
| GO:0044421 | CC | extracellular region part | 9.64E-12 | 1.64E-07 | 179 | 2,119 |
| GO:0002376 | BP | immune system process | 7.79E-10 | 1.33E-05 | 72 | 619 |
| GO:0005615 | CC | extracellular space | 6.26E-09 | 0.000107 | 75 | 695 |
| GO:0003676 | MF | nucleic acid binding | 2.67E-08 | 0.000454 | 65 | 2,268 |
| GO:0006396 | BP | RNA processing | 7.67E-08 | 0.001306 | 1 | 397 |
| GO:0002682 | BP | regulation of immune system process | 2.21E-07 | 0.003765 | 62 | 579 |
| GO:0006955 | BP | immune response | 1.11E-06 | 0.018898 | 38 | 297 |
| GO:0030529 | CC | intracellular ribonucleoprotein complex | 1.54E-06 | 0.026195 | 4 | 460 |
| GO:0050865 | BP | regulation of cell activation | 1.66E-06 | 0.028256 | 34 | 249 |
| GO:0016070 | BP | RNA metabolic process | 2.50E-06 | 0.04263 | 29 | 1,208 |
| GO:0050863 | BP | regulation of T cell activation | 2.89E-06 | 0.049235 | 24 | 147 |
| GO:0009897 | CC | external side of plasma membrane | 2.91E-06 | 0.049566 | 21 | 116 |

## Table S9. GO enrichment analysis of 1,509 DEGs in the second phase during rumen development. BP denotes Biological Process, MF denotes Molecular Function, and CC denotes Cellular Component.

| **ID** | **Type** | **Description** | **Fisher's exact test *P* value** | **Adjusted *P* value** | **Input number** | **Background number** |
| --- | --- | --- | --- | --- | --- | --- |
| GO:0002376 | BP | immune system process | 8.12E-07 | 0.013837 | 86 | 619 |
| GO:0003676 | MF | nucleic acid binding | 1.43E-11 | 2.44E-07 | 98 | 2,268 |
| GO:0003723 | MF | RNA binding | 3.98E-07 | 0.006787 | 34 | 945 |
| GO:0005615 | CC | extracellular space | 9.29E-12 | 1.58E-07 | 111 | 695 |
| GO:0005634 | CC | nucleus | 4.39E-13 | 7.48E-09 | 140 | 3,014 |
| GO:0005886 | CC | plasma membrane | 4.45E-08 | 0.000758 | 187 | 1,614 |
| GO:0006139 | BP | nucleobase-containing compound metabolic process | 1.34E-10 | 2.28E-06 | 67 | 1,710 |
| GO:0006396 | BP | RNA processing | 1.98E-07 | 0.003375 | 6 | 397 |
| GO:0006631 | BP | fatty acid metabolic process | 2.44E-06 | 0.041484 | 28 | 122 |
| GO:0006725 | BP | cellular aromatic compound metabolic process | 8.26E-09 | 0.000141 | 77 | 1,775 |
| GO:0006807 | BP | nitrogen compound metabolic process | 2.98E-09 | 5.07E-05 | 79 | 1,833 |
| GO:0006974 | BP | cellular response to DNA damage stimulus | 4.36E-07 | 0.007425 | 5 | 356 |
| GO:0009611 | BP | response to wounding | 1.52E-06 | 0.025964 | 40 | 211 |
| GO:0016021 | CC | integral component of membrane | 1.71E-07 | 0.002904 | 295 | 2,886 |
| GO:0016070 | BP | RNA metabolic process | 4.30E-07 | 0.007317 | 49 | 1,208 |
| GO:0019752 | BP | carboxylic acid metabolic process | 2.50E-06 | 0.042593 | 52 | 315 |
| GO:0030529 | CC | intracellular ribonucleoprotein complex | 7.36E-08 | 0.001253 | 8 | 460 |
| GO:0031224 | CC | intrinsic component of membrane | 1.30E-08 | 0.000222 | 306 | 2,934 |
| GO:0031982 | CC | vesicle | 1.18E-08 | 0.000201 | 219 | 1,932 |
| GO:0032787 | BP | monocarboxylic acid metabolic process | 3.98E-09 | 6.78E-05 | 41 | 171 |
| GO:0032991 | CC | macromolecular complex | 2.71E-12 | 4.61E-08 | 130 | 2,814 |
| GO:0034641 | BP | cellular nitrogen compound metabolic process | 1.83E-09 | 3.13E-05 | 77 | 1,812 |
| GO:0043170 | BP | macromolecule metabolic process | 7.49E-08 | 0.001276 | 198 | 3,518 |
| GO:0043229 | CC | intracellular organelle | 2.90E-07 | 0.004941 | 367 | 5,753 |
| GO:0043230 | CC | extracellular organelle | 1.66E-07 | 0.002835 | 181 | 1,574 |
| GO:0043231 | CC | intracellular membrane-bounded organelle | 1.01E-07 | 0.001727 | 318 | 5,151 |
| GO:0043234 | CC | protein complex | 1.72E-08 | 0.000292 | 121 | 2,440 |
| GO:0043436 | BP | oxoacid metabolic process | 2.50E-06 | 0.042593 | 52 | 315 |
| GO:0044260 | BP | cellular macromolecule metabolic process | 1.82E-12 | 3.10E-08 | 156 | 3,219 |
| GO:0044421 | CC | extracellular region part | 4.27E-13 | 7.27E-09 | 256 | 2,119 |
| GO:0044422 | CC | organelle part | 2.73E-11 | 4.65E-07 | 241 | 4,367 |
| GO:0044424 | CC | intracellular part | 1.62E-07 | 0.002767 | 511 | 7,585 |
| GO:0044427 | CC | chromosomal part | 2.41E-07 | 0.004103 | 9 | 462 |
| GO:0044428 | CC | nuclear part | 1.97E-16 | 3.36E-12 | 93 | 2,443 |
| GO:0044446 | CC | intracellular organelle part | 1.10E-13 | 1.87E-09 | 225 | 4,294 |
| GO:0044451 | CC | nucleoplasm part | 9.61E-09 | 0.000164 | 23 | 819 |
| GO:0046483 | BP | heterocycle metabolic process | 2.05E-10 | 3.49E-06 | 72 | 1,782 |
| GO:0051239 | BP | regulation of multicellular organismal process | 2.90E-07 | 0.004935 | 176 | 1,537 |
| GO:0051240 | BP | positive regulation of multicellular organismal process | 3.95E-07 | 0.006725 | 61 | 376 |
| GO:0051276 | BP | chromosome organization | 1.11E-08 | 0.000189 | 6 | 441 |
| GO:0065010 | CC | extracellular membrane-bounded organelle | 1.66E-07 | 0.002835 | 181 | 1,574 |
| GO:0070062 | CC | extracellular exosome | 1.29E-07 | 0.002196 | 181 | 1,573 |
| GO:0090304 | BP | nucleic acid metabolic process | 2.03E-11 | 3.46E-07 | 54 | 1521 |
| GO:1901360 | BP | organic cyclic compound metabolic process | 6.65E-08 | 0.001132 | 86 | 1858 |

## Table S10. KEGG enrichment results for the DEGs during early rumen development in the first phase.

| **Term** | **ID** | **Input number** | **Background number** | ***P*-Value** | **Corrected *P*-Value** | **Input** |
| --- | --- | --- | --- | --- | --- | --- |
| Chagas disease (American trypanosomiasis) | chx05142 | 16 | 111 | 4.91E-06 | 0.000687 | NOS2\|NFKBIA\|PLCB4\|CCL5\|IL10\|CXCL8\|KNG1\|C1QC\|C1QB\|C1QA\|TNF\|PIK3R3\|C3\|CD3D\|CD3E\|CD3G |
| Primary immunodeficiency | chx05340 | 9 | 34 | 9.54E-06 | 0.000891 | TAP1\|ICOS\|IL2RG\|CIITA\|PTPRC\|CD8A\|CD3D\|CD3E\|CD79A |
| Human T-cell leukemia virus 1 infection | chx05166 | 23 | 232 | 1.54E-05 | 0.001077 | BUB1B\|NFKBIA\|B2M\|IL1R2\|PIK3R3\|JAK3\|MAP3K14\|PTTG1\|ITGB2\|CREB5\|SLC25A5\|TNF\|RELB\|CCNB2\|E2F2\|IL2RG\|CCND3\|CDC20\|ITGAL\|ELK4\|CD3D\|CD3E\|CD3G |
| Viral protein interaction with cytokine and cytokine receptor | chx04061 | 14 | 99 | 2.30E-05 | 0.001186 | CCR7\|IL10RA\|CX3CR1\|CCL5\|IL10\|CXCL8\|IL2RG\|XCR1\|CXCR3\|TNF\|CCL19\|CCL20\|CCL22\|CXCL14 |
| T cell receptor signaling pathway | chx04660 | 14 | 100 | 2.54E-05 | 0.001186 | ICOS\|RASGRP1\|IL10\|VAV3\|NFKBIA\|PIK3R3\|TNF\|PTPRC\|LCP2\|CD8A\|MAP3K14\|CD3D\|CD3E\|CD3G |
| Ether lipid metabolism | chx00565 | 10 | 53 | 3.98E-05 | 0.001592 | PLD4\|AGPS\|PLD1\|PLA2G2F\|GDPD3\|PLA2G2C\|PLA2G3\|PLB1\|PLA2G4F\|PLA2G4E |
| Chemokine signaling pathway | chx04062 | 19 | 184 | 5.01E-05 | 0.001754 | CCL20\|NFKBIA\|PLCB4\|CX3CR1\|CCL5\|CXCL8\|VAV3\|CCR7\|XCR1\|CCL19\|CXCR3\|PIK3R3\|JAK3\|HCK\|RAC2\|CCL22\|WAS\|CXCL14\|CXCL16 |
| Influenza A | chx05164 | 18 | 175 | 8.21E-05 | 0.002531 | RSAD2\|DDX58\|NFKBIA\|IRF7\|SOCS3\|CCL5\|CXCL8\|CCND3\|EIF2AK2\|TMPRSS4\|SLC25A5\|MX1\|TMPRSS2\|TNF\|PIK3R3\|KPNA2\|CIITA\|RNASEL |
| Sulfur metabolism | chx00920 | 5 | 10 | 9.04E-05 | 0.002531 | SELENBP1\|PAPSS2\|BPNT1\|SUOX\|SQRDL |
| Epstein-Barr virus infection | chx05169 | 20 | 215 | 0.000121 | 0.00308 | TAP1\|DDX58\|NFKBIA\|IRF7\|E2F2\|FCER2\|PIK3R3\|CCND3\|EIF2AK2\|MAP3K14\|GADD45G\|B2M\|TNF\|ISG15\|JAK3\|RELB\|ITGAL\|CD3D\|CD3E\|CD3G |
| Staphylococcus aureus infection | chx05150 | 10 | 64 | 0.000161 | 0.003582 | MAP28\|ITGB2\|IL10\|CFD\|C1QC\|C1QB\|C1QA\|SELPLG\|ITGAL\|C3 |
| p53 signaling pathway | chx04115 | 11 | 78 | 0.000174 | 0.003582 | CCNB2\|CCNB1\|IGFBP3\|CCND3\|GTSE1\|THBS1\|SFN\|CDK1\|RRM2\|BBC3\|GADD45G |
| Hematopoietic cell lineage | chx04640 | 13 | 107 | 0.000179 | 0.003582 | CD38\|TFRC\|FCER2\|ITGA4\|IL1R2\|CD5\|TNF\|CD2\|CD8A\|CD24\|CD3D\|CD3E\|CD3G |
| Cell cycle | chx04110 | 14 | 126 | 0.00024 | 0.004475 | CCNB2\|CCNB1\|BUB1\|E2F2\|BUB1B\|CDKN1C\|CCND3\|TTK\|CDC20\|SFN\|CDK1\|PTTG1\|GADD45G\|CDC6 |
| Measles | chx05162 | 15 | 144 | 0.000277 | 0.004841 | DDX58\|NFKBIA\|IRF7\|HSPA6\|IL2RG\|CCND3\|EIF2AK2\|MX1\|CD209\|PIK3R3\|JAK3\|BBC3\|CD3D\|CD3E\|CD3G |
| MAPK signaling pathway | chx04010 | 23 | 299 | 0.000511 | 0.00842 | MAP4K1\|PDGFRA\|RASGRP1\|HSPA6\|JUND\|NGFR\|FLT4\|NTRK2\|GADD45G\|PLA2G4E\|RELB\|PLA2G4F\|DUSP8\|RAC2\|NR4A1\|VEGFD\|TNF\|PTPN7\|DUSP10\|MAPK8IP2\|RPS6KA5\|MAP3K14\|ELK4 |
| Complement and coagulation cascades | chx04610 | 11 | 91 | 0.000574 | 0.008928 | ITGB2\|PLAUR\|CFD\|KNG1\|C1QC\|C1QB\|C1QA\|F3\|TFPI\|C3\|F13A1 |
| Pertussis | chx05133 | 10 | 77 | 0.00061 | 0.008992 | NOS2\|ITGB2\|IL10\|CXCL8\|IRF8\|C1QC\|C1QB\|C1QA\|TNF\|C3 |
| Malaria | chx05144 | 8 | 52 | 0.00079 | 0.011055 | ITGB2\|IL10\|CXCL8\|HBBC\|THBS3\|THBS1\|TNF\|ITGAL |
| Cytokine-cytokine receptor interaction | chx04060 | 24 | 335 | 0.00099 | 0.013206 | IL10RA\|CCL5\|NGFR\|CCR7\|IL1R2\|XCR1\|CXCR3\|INHBB\|CCL20\|GDF15\|CCL22\|IL17D\|TNF\|CXCL14\|CXCL17\|CXCL16\|BMP6\|IL36B\|CX3CR1\|IL2RG\|IL10\|CXCL8\|IL16\|CCL19 |
| alpha-Linolenic acid metabolism | chx00592 | 6 | 31 | 0.001264 | 0.016053 | PLB1\|PLA2G2C\|PLA2G3\|PLA2G2F\|PLA2G4F\|PLA2G4E |
| Legionellosis | chx05134 | 8 | 57 | 0.001347 | 0.016053 | ITGB2\|HSPA6\|CXCL8\|NFKBIA\|TNF\|C3\|SAR1B\|BNIP3 |
| Focal adhesion | chx04510 | 17 | 207 | 0.001376 | 0.016053 | PDGFRA\|RAC2\|ITGB7\|LAMB3\|VAV3\|CCND3\|COL9A2\|THBS3\|THBS1\|VEGFD\|ITGA10\|PIK3R3\|PPP1R12C\|LAMC2\|FLT4\|ITGA4\|PARVG |
| TNF signaling pathway | chx04668 | 12 | 120 | 0.00151 | 0.016908 | CEBPB\|NFKBIA\|RPS6KA5\|SOCS3\|CCL5\|CREB5\|MMP9\|VEGFD\|TNF\|MAP3K14\|CCL20\|PIK3R3 |
| Cell adhesion molecules (CAMs) | chx04514 | 14 | 158 | 0.001847 | 0.01989 | CLDN10\|ITGB2\|CD2\|ITGB7\|ITGA4\|ICOS\|CD86\|CNTN2\|CLDN11\|ITGAL\|SPN\|CD8A\|PTPRC\|SELPLG |
| Fc gamma R-mediated phagocytosis | chx04666 | 10 | 96 | 0.002772 | 0.027491 | INPP5D\|RAC2\|PLD1\|VAV3\|PIK3R3\|PTPRC\|HCK\|WAS\|PLA2G4F\|PLA2G4E |
| Antigen processing and presentation | chx04612 | 9 | 80 | 0.002777 | 0.027491 | TAP1\|CD74\|CTSL\|HSPA6\|CIITA\|B2M\|TNF\|CD8A\|CTSS |
| Fc epsilon RI signaling pathway | chx04664 | 8 | 65 | 0.002847 | 0.027491 | INPP5D\|VAV3\|TNF\|PIK3R3\|LCP2\|RAC2\|PLA2G4F\|PLA2G4E |
| Toxoplasmosis | chx05145 | 11 | 114 | 0.003015 | 0.028137 | NOS2\|SOCS1\|HSPA6\|IL10\|CIITA\|NFKBIA\|TNF\|LAMC2\|PPIF\|LAMB3\|IL10RA |
| ECM-receptor interaction | chx04512 | 10 | 98 | 0.003177 | 0.028698 | HMMR\|ITGB7\|ITGA4\|COL9A2\|THBS3\|THBS1\|FREM2\|ITGA10\|LAMC2\|LAMB3 |
| Tuberculosis | chx05152 | 15 | 188 | 0.003307 | 0.028932 | CD74\|MRC1\|NOS2\|ITGB2\|IL10RA\|CEBPB\|IL10\|CIITA\|CLEC7A\|MAP28\|CD209\|CORO1A\|C3\|CTSS\|TNF |
| Arachidonic acid metabolism | chx00590 | 9 | 83 | 0.003478 | 0.029508 | PLA2G2F\|PLA2G2C\|GPX2\|PLA2G3\|PLB1\|LTA4H\|ALOX12B\|PLA2G4F\|PLA2G4E |
| Transcriptional misregulation in cancer | chx05202 | 15 | 190 | 0.003625 | 0.029853 | CEBPB\|ZBTB16\|ITGB7\|IGFBP3\|CXCL8\|TMPRSS2\|ELK4\|GADD45G\|PER2\|PAX8\|NGFR\|REL\|MMP9\|CD86\|IL1R2 |
| C-type lectin receptor signaling pathway | chx04625 | 10 | 101 | 0.003872 | 0.030791 | IL17D\|CLEC7A\|IL10\|NFKBIA\|CD209\|TNF\|MAP3K14\|RELB\|PIK3R3\|CCL22 |
| Linoleic acid metabolism | chx00591 | 6 | 40 | 0.003959 | 0.030791 | PLB1\|PLA2G2C\|PLA2G3\|PLA2G2F\|PLA2G4F\|PLA2G4E |
| Hepatitis C | chx05160 | 13 | 159 | 0.004977 | 0.037666 | RSAD2\|CLDN10\|CLDN11\|IRF7\|E2F2\|SOCS3\|EIF2AK2\|DDX58\|MX1\|TNF\|PIK3R3\|NFKBIA\|RNASEL |
| PPAR signaling pathway | chx03320 | 9 | 89 | 0.00529 | 0.038982 | DBI\|HMGCS2\|CPT2\|LPL\|ME1\|FABP5\|UCP1\|ADIPOQ\|PLIN1 |
| IL-17 signaling pathway | chx04657 | 9 | 90 | 0.005653 | 0.040024 | IL17D\|CEBPB\|CXCL8\|FOSB\|NFKBIA\|JUND\|TNF\|MMP9\|CCL20 |
| Osteoclast differentiation | chx04380 | 11 | 125 | 0.005718 | 0.040024 | SOCS1\|RELB\|SOCS3\|TYROBP\|FOSB\|NFKBIA\|JUND\|TNF\|PIK3R3\|LCP2\|MAP3K14 |
| Amoebiasis | chx05146 | 10 | 108 | 0.005962 | 0.040719 | NOS2\|ITGB2\|PLCB4\|IL10\|CXCL8\|TNF\|PIK3R3\|LAMC2\|LAMB3\|IL1R2 |
| Yersinia infection | chx05135 | 11 | 128 | 0.006715 | 0.044763 | FYB\|RAC2\|IL10\|CXCL8\|VAV3\|NFKBIA\|ITGA4\|TNF\|PIK3R3\|LCP2\|WAS |
| Circadian rhythm | chx04710 | 5 | 32 | 0.007183 | 0.04633 | BHLHE41\|PER1\|RORC\|CRY2\|PER2 |
| Type II diabetes mellitus | chx04930 | 6 | 46 | 0.00728 | 0.04633 | SOCS1\|SOCS3\|IRS2\|TNF\|PIK3R3\|ADIPOQ |
| Intestinal immune network for IgA production | chx04672 | 6 | 47 | 0.007985 | 0.049685 | ICOS\|ITGB7\|IL10\|ITGA4\|CD86\|MAP3K14 |

## Table S11. KEGG enrichment results for the DEGs during early rumen development in the second phase.

| **Term** | **ID** | **Input number** | **Background number** | ***P*-Value** | **Corrected *P*-Value** | **Input** |
| --- | --- | --- | --- | --- | --- | --- |
| Focal adhesion | chx04510 | 32 | 207 | 1.73E-07 | 2.38E-05 | ITGA9\|COL4A1\|ITGA1\|ITGA5\|ITGA7\|LAMA4\|TLN1\|COL2A1\|ZYX\|PGF\|THBS4\|TNXB\|THBS1\|RELN\|PPP1R12B\|PPP1R12C\|PARVB\|PARVG\|PDGFA\|RAC2\|ITGB7\|PRKCA\|VCL\|PRKCB\|COL9A2\|COL4A6\|COL4A2\|TNC\|LAMC1\|ACTN1\|SPP1\|FLNC |
| ECM-receptor interaction | chx04512 | 21 | 98 | 2.32E-07 | 2.38E-05 | ITGA9\|LAMA4\|TNC\|ITGB7\|COL9A2\|THBS4\|HSPG2\|TNXB\|ITGA5\|CD36\|THBS1\|COL4A6\|SPP1\|COL4A2\|COL4A1\|ITGA1\|FRAS1\|LAMC1\|COL2A1\|ITGA7\|RELN |
| PPAR signaling pathway | chx03320 | 19 | 89 | 9.05E-07 | 6.97E-05 | PCK2\|HMGCS2\|PCK1\|SCD\|ACSL1\|CD36\|ACSL5\|FABP7\|LPL\|ME1\|ADIPOQ\|ME3\|SORBS1\|PLIN4\|SLC27A6\|PLIN2\|APOA1\|APOA2\|PLIN1 |
| Salivary secretion | chx04970 | 17 | 97 | 3.23E-05 | 0.001771 | CD38\|MAP28\|PLCB2\|AQP5\|ADCY2\|ADCY5\|PRKCA\|ATP1B2\|PRKCB\|CHRM3\|KCNMA1\|ADRB3\|KCNN4\|ATP1A3\|BST1\|ATP2B3\|ATP2B4 |
| Amoebiasis | chx05146 | 18 | 108 | 3.45E-05 | 0.001771 | IL10\|ACTN1\|ITGB2\|LAMA4\|PRKCA\|ARG1\|PRKCB\|VCL\|NOS2\|PLCB2\|COL4A6\|GNA14\|TGFB3\|COL4A2\|COL4A1\|LAMC1\|IL1R2\|GNA15 |
| Aldosterone synthesis and secretion | chx04925 | 17 | 103 | 6.28E-05 | 0.002418 | ADCY5\|PLCB2\|PRKCB\|LIPE\|KCNK9\|ADCY2\|CACNA1C\|PRKCA\|CREB5\|NR4A1\|ATP1B2\|KCNK3\|CREB3L3\|ATP1A3\|ATP2B3\|CACNA1G\|ATP2B4 |
| Pancreatic secretion | chx04972 | 17 | 103 | 6.28E-05 | 0.002418 | CD38\|ADCY5\|PLCB2\|ADCY2\|PRKCA\|ATP1B2\|PRKCB\|CHRM3\|KCNMA1\|SLC4A4\|SLC26A3\|ATP1A3\|PLA2G3\|PLA2G2F\|BST1\|ATP2B3\|ATP2B4 |
| cAMP signaling pathway | chx04024 | 29 | 247 | 7.27E-05 | 0.002486 | NFATC1\|RRAS\|SOX9\|LIPE\|PPP1R1B\|CREB3L3\|CACNA1C\|ATP2B4\|GLI1\|TNNI3\|GLP1R\|EDN1\|EDN3\|RAC2\|FOS\|CREB5\|ATP1B2\|HCAR2\|ATP2B3\|GRIN1\|ADCY5\|PLD1\|ADCY2\|SST\|HCN2\|DRD2\|GRIN2C\|ATP1A3\|HTR1B |
| Thyroid hormone synthesis | chx04918 | 14 | 75 | 8.69E-05 | 0.002678 | ADCY5\|PLCB2\|DUOXA2\|ADCY2\|CREB3L3\|PRKCA\|CREB5\|PRKCB\|ATP1B2\|GSR\|TPO\|GPX3\|GPX2\|ATP1A3 |
| MAPK signaling pathway | chx04010 | 32 | 299 | 0.000154 | 0.004304 | MAP4K1\|NFATC1\|TGFB3\|HSPA2\|RRAS\|NGFR\|MAPK13\|TGFA\|AREG\|CACNB2\|CACNA1C\|NTRK2\|CACNA1G\|FGF18\|PLA2G4E\|DUSP5\|DUSP4\|DUSP8\|IGF2\|PDGFA\|NR4A1\|RAC2\|FOS\|PRKCA\|PRKCB\|PGF\|RELB\|PTPN7\|PLA2G4F\|MAPK8IP2\|EREG\|FLNC |
| Adrenergic signaling in cardiomyocytes | chx04261 | 20 | 155 | 0.000307 | 0.007881 | PPP1R1A\|ADCY5\|PLCB2\|ADCY2\|PRKCA\|CACNB2\|CREB5\|CACNA1C\|ATP1B2\|PPP2R2C\|MAPK13\|CREB3L3\|MYL4\|ATP1A3\|TNNI3\|ACTC1\|SLC8A1\|ATP2B3\|ATP2B4\|CREM |
| Pertussis | chx05133 | 13 | 77 | 0.000367 | 0.008341 | NOS2\|ITGB2\|FOS\|IL10\|IRF8\|C1QC\|C1QB\|C1QA\|ITGA5\|SERPING1\|CFL2\|MAPK13\|C3 |
| Insulin secretion | chx04911 | 14 | 88 | 0.000379 | 0.008341 | ADCY5\|PLCB2\|CACNA1C\|ADCY2\|PRKCA\|ATP1B2\|CREB5\|PRKCB\|CHRM3\|KCNMA1\|CREB3L3\|KCNN4\|ATP1A3\|GLP1R |
| PI3K-Akt signaling pathway | chx04151 | 36 | 375 | 0.000426 | 0.00845 | PCK2\|ITGA9\|PCK1\|COL4A1\|PDGFA\|ITGA1\|ITGA5\|ITGA7\|NGFR\|COL2A1\|TGFA\|AREG\|PRLR\|THBS4\|TNXB\|NTRK2\|THBS1\|FGF18\|RELN\|GNB3\|IGF2\|PGF\|LAMA4\|ITGB7\|PRKCA\|CREB5\|NR4A1\|COL9A2\|PPP2R2C\|COL4A6\|COL4A2\|TNC\|LAMC1\|CREB3L3\|SPP1\|EREG |
| Proximal tubule bicarbonate reclamation | chx04964 | 7 | 23 | 0.000453 | 0.00845 | PCK2\|PCK1\|SLC9A3\|ATP1B2\|CA4\|SLC4A4\|ATP1A3 |
| Amphetamine addiction | chx05031 | 12 | 69 | 0.000482 | 0.00845 | ADCY5\|CACNA1C\|GRIN2C\|PRKCA\|FOSB\|PRKCB\|CREB3L3\|PPP1R1B\|TH\|GRIN1\|CREB5\|FOS |
| AMPK signaling pathway | chx04152 | 17 | 125 | 0.000494 | 0.00845 | PCK2\|PCK1\|LIPE\|ACACB\|SCD\|PFKFB3\|PFKFB4\|PPARGC1A\|PPP2R2C\|PFKP\|CREB3L3\|FASN\|CD36\|FBP2\|CAMKK2\|CREB5\|ADIPOQ |
| Fatty acid metabolism | chx01212 | 11 | 60 | 0.000555 | 0.009001 | HADH\|ACADS\|ACAA2\|SCD\|ACSL1\|ACSL5\|ELOVL6\|ELOVL7\|ELOVL4\|FASN\|ACADSB |
| Cocaine addiction | chx05030 | 10 | 51 | 0.000621 | 0.009556 | ADCY5\|PPP1R1B\|GRIN2C\|FOSB\|DRD2\|CREB3L3\|CDK5R1\|TH\|GRIN1\|CREB5 |
| Arachidonic acid metabolism | chx00590 | 13 | 83 | 0.00069 | 0.010121 | PTGS2\|EPHX2\|PTGIS\|GPX3\|GPX2\|GGT1\|PLA2G3\|PLA2G2F\|PTGES\|ALOX12B\|PLA2G4F\|PLA2G4E\|ALOX12 |
| Calcium signaling pathway | chx04020 | 22 | 202 | 0.001264 | 0.017367 | CD38\|PLCB2\|EDNRB\|ADORA2B\|CASQ1\|CACNA1C\|CACNA1G\|GNA15\|HRH1\|SLC8A1\|GRIN1\|NOS2\|PRKCA\|PRKCB\|CHRM3\|ATP2B3\|ATP2B4\|ADCY2\|GRIN2C\|GNA14\|ADRB3\|TACR2 |
| Human papillomavirus infection | chx05165 | 33 | 357 | 0.001297 | 0.017367 | PTGS2\|COL4A1\|ITGA9\|ITGA1\|MX1\|ITGA5\|ITGA7\|COL2A1\|WNT10B\|TNXB\|THBS1\|ISG15\|RELN\|TERT\|LAMA4\|ITGB7\|CREB5\|COL9A2\|PPP2R2C\|COL4A6\|COL4A2\|TNC\|LAMC1\|HEY1\|HES4\|EIF2AK2\|WNT4\|CREB3L3\|SPP1\|WNT5B\|WNT5A\|WNT7A\|THBS4 |
| Vascular smooth muscle contraction | chx04270 | 17 | 140 | 0.001533 | 0.019675 | ADCY5\|ADORA2B\|PRKCB\|ADCY2\|PRKCA\|PPP1R12C\|PLCB2\|KCNMA1\|CACNA1C\|PLA2G3\|PPP1R12B\|PLA2G2F\|EDN1\|EDN3\|CALD1\|PLA2G4F\|PLA2G4E |
| Mineral absorption | chx04978 | 9 | 50 | 0.001937 | 0.022655 | SLC9A3\|ATP1B2\|HMOX1\|SLC26A3\|ATP1A3\|CLCN2\|SLC8A1\|SLC26A9\|TRPM6 |
| Dopaminergic synapse | chx04728 | 16 | 131 | 0.00197 | 0.022655 | CALY\|PLCB2\|KIF5C\|FOS\|PRKCA\|ADCY5\|CREB5\|PRKCB\|DRD2\|PPP2R2C\|CREB3L3\|PPP1R1B\|GNB3\|TH\|MAPK13\|CACNA1C |
| Relaxin signaling pathway | chx04926 | 17 | 144 | 0.002012 | 0.022655 | ADCY5\|NOS2\|ADCY2\|FOS\|PRKCA\|CREB5\|PLCB2\|GNB3\|CREB3L3\|GNA15\|COL4A2\|COL4A1\|MAPK13\|EDN1\|EDNRB\|MMP2\|COL4A6 |
| Glutathione metabolism | chx00480 | 10 | 61 | 0.00206 | 0.022655 | GSR\|GSTM3\|GCLC\|MGST3\|GPX3\|GPX2\|GGT6\|ANPEP\|ODC1\|GGT1 |
| Dilated cardiomyopathy (DCM) | chx05414 | 13 | 98 | 0.002648 | 0.026797 | ADCY5\|TGFB3\|ADCY2\|ITGB7\|ITGA9\|CACNB2\|CACNA1C\|ITGA5\|ITGA7\|TNNI3\|ITGA1\|SLC8A1\|ACTC1 |
| Staphylococcus aureus infection | chx05150 | 10 | 64 | 0.002815 | 0.026797 | MAP28\|ITGB2\|IL10\|C1QC\|C1QB\|C1QA\|SELPLG\|ITGAL\|C3\|DSG1 |
| Chagas disease (American trypanosomiasis) | chx05142 | 14 | 111 | 0.002815 | 0.026797 | NOS2\|TGFB3\|FOS\|PLCB2\|C1QC\|C1QB\|PPP2R2C\|IL10\|GNA14\|GNA15\|C1QA\|MAPK13\|C3\|SERPINE1 |
| Proteoglycans in cancer | chx05205 | 21 | 203 | 0.002832 | 0.026797 | HPSE\|IGF2\|HSPG2\|CTSL\|MAPK13\|PRKCA\|WNT7A\|WNT10B\|PRKCB\|ITGA5\|THBS1\|RRAS\|SHH\|WNT4\|PPP1R12B\|PPP1R12C\|HCLS1\|WNT5B\|WNT5A\|FLNC\|MMP2 |
| Wnt signaling pathway | chx04310 | 18 | 163 | 0.002926 | 0.026797 | RSPO3\|NFATC1\|PRICKLE2\|RAC2\|VANGL2\|SFRP5\|PRKCA\|DAAM2\|PRKCB\|PLCB2\|WNT4\|BAMBI\|WIF1\|FOSL1\|WNT5B\|WNT5A\|WNT7A\|WNT10B |
| Glutamatergic synapse | chx04724 | 14 | 112 | 0.00303 | 0.026797 | ADCY5\|PLCB2\|CACNA1C\|PLD1\|ADCY2\|PRKCA\|PRKCB\|GNB3\|GRIN2C\|GRIN1\|HOMER2\|PLA2G4F\|PLA2G4E\|SLC1A3 |
| Melanogenesis | chx04916 | 13 | 100 | 0.003101 | 0.026797 | ADCY5\|PLCB2\|ADCY2\|PRKCA\|WNT10B\|PRKCB\|WNT4\|CREB3L3\|EDN1\|WNT5B\|WNT5A\|WNT7A\|EDNRB |
| Apelin signaling pathway | chx04371 | 16 | 138 | 0.00316 | 0.026797 | ADCY5\|NOS2\|CTGF\|LIPE\|ADCY2\|PLCB2\|EGR1\|PPARGC1A\|GNB3\|RRAS\|MYL4\|SPP1\|SLC8A1\|APLNR\|SERPINE1\|PLIN1 |
| Cytokine-cytokine receptor interaction | chx04060 | 30 | 335 | 0.003228 | 0.026797 | TGFB3\|IL17F\|BMPR1B\|NGFR\|CCR7\|TNFRSF12A\|IL1R2\|IFNLR1\|PRLR\|XCR1\|CXCR3\|CXCR2\|INHBB\|IL22RA1\|IL36B\|TNFRSF6B\|IL36RN\|IL17B\|LTB\|TNFRSF11A\|CXCL10\|CXCL17\|CXCL16\|INHA\|BMP6\|BMP2\|CXCL9\|IL10\|EDAR\|IL17RE |
| Gastric acid secretion | chx04971 | 11 | 77 | 0.003306 | 0.026797 | ATP1B2\|ADCY5\|PLCB2\|ATP4A\|KCNJ15\|PRKCA\|SST\|PRKCB\|CHRM3\|ATP1A3\|ADCY2 |
| Pathways in cancer | chx05200 | 43 | 537 | 0.003529 | 0.027873 | PTGS2\|TGFB3\|PDGFA\|PLCB2\|RET\|NQO1\|MGST3\|LAMA4\|EDNRB\|TGFA\|ZBTB16\|WNT10B\|FGF18\|EDN1\|HEY1\|IGF2\|PGF\|NOS2\|RAC2\|FOS\|PRKCA\|PRKCB\|GNB3\|COL4A6\|COL4A2\|COL4A1\|SHH\|LAMC1\|BMP2\|EGLN3\|ADCY5\|TERT\|ALK\|ADCY2\|MMP2\|GSTM3\|HMOX1\|WNT4\|PTCH2\|WNT5B\|WNT5A\|WNT7A\|GLI1 |
| IL-17 signaling pathway | chx04657 | 12 | 90 | 0.003661 | 0.028187 | PTGS2\|IL17F\|IL17B\|FOS\|S100A9\|FOSB\|FOSL1\|S100A8\|MAPK4\|MAPK13\|CXCL10\|IL17RE |
| Cortisol synthesis and secretion | chx04927 | 10 | 67 | 0.003777 | 0.028371 | ADCY5\|PLCB2\|CACNA1C\|ADCY2\|CREB5\|NR4A1\|KCNK3\|CREB3L3\|CACNA1G\|PDE8B |
| Rap1 signaling pathway | chx04015 | 21 | 211 | 0.004271 | 0.031318 | PGF\|PDGFA\|ADORA2B\|ITGB2\|RAC2\|ADCY2\|ADCY5\|PRKCA\|ENAH\|PRKCB\|DRD2\|PLCB2\|THBS1\|RRAS\|FGF18\|ITGAL\|NGFR\|MAPK13\|FYB\|GRIN1\|TLN1 |
| Protein digestion and absorption | chx04974 | 16 | 144 | 0.004602 | 0.032473 | ATP1B2\|COL15A1\|ELN\|COL7A1\|SLC9A3\|KCNK5\|COL9A2\|COL4A6\|KCNN4\|ATP1A3\|COL4A2\|COL4A1\|SLC8A1\|ACE2\|COL5A1\|COL2A1 |
| GnRH signaling pathway | chx04912 | 12 | 93 | 0.004639 | 0.032473 | ADCY5\|PLCB2\|EGR1\|PLD1\|ADCY2\|PRKCA\|PRKCB\|CACNA1C\|MAPK13\|MMP2\|PLA2G4F\|PLA2G4E |
| Leishmaniasis | chx05140 | 10 | 70 | 0.004982 | 0.033941 | MARCKSL1\|PTGS2\|NOS2\|ITGB2\|FOS\|IL10\|PRKCB\|TGFB3\|MAPK13\|C3 |
| Arrhythmogenic right ventricular cardiomyopathy (ARVC) | chx05412 | 11 | 82 | 0.005069 | 0.033941 | ACTN3\|ACTN2\|ITGB7\|ITGA9\|CACNB2\|CACNA1C\|ITGA5\|ITGA7\|DSG2\|ITGA1\|SLC8A1 |
| Serotonergic synapse | chx04726 | 14 | 120 | 0.00528 | 0.0346 | PTGS2\|PLCB2\|CACNA1C\|ADCY5\|PRKCA\|PRKCB\|GNB3\|PLA2G4E\|SLC6A4\|GABRB2\|ALOX12B\|PLA2G4F\|HTR1B\|ALOX12 |
| Hypertrophic cardiomyopathy (HCM) | chx05410 | 12 | 95 | 0.0054 | 0.034649 | ITGA9\|TGFB3\|ITGB7\|CACNB2\|CACNA1C\|ITGA5\|ITGA7\|EDN1\|TNNI3\|ITGA1\|SLC8A1\|ACTC1 |
| Inflammatory mediator regulation of TRP channels | chx04750 | 13 | 109 | 0.005976 | 0.037562 | ADCY5\|PLCB2\|ADCY2\|PRKCA\|PRKCB\|ASIC1\|MAPK13\|ALOX12\|HRH1\|P2RY2\|TRPA1\|PLA2G4F\|PLA2G4E |
| Pyruvate metabolism | chx00620 | 7 | 39 | 0.006102 | 0.037587 | PCK2\|PCK1\|ACACB\|PC\|ME1\|ME3\|LDHD |
| Leukocyte transendothelial migration | chx04670 | 13 | 111 | 0.006839 | 0.04074 | ACTN1\|ITGB2\|RAC2\|CLDN17\|PRKCA\|PRKCB\|VCL\|CLDN23\|ITGAL\|MAPK13\|MMP2\|CLDN5\|CLDN7 |
| African trypanosomiasis | chx05143 | 7 | 40 | 0.006878 | 0.04074 | PLCB2\|HBBC\|LAMA4\|PRKCA\|PRKCB\|IL10\|APOA1 |
| Basal cell carcinoma | chx05217 | 9 | 63 | 0.007534 | 0.043783 | BMP2\|WNT10B\|WNT4\|PTCH2\|GLI1\|SHH\|WNT5B\|WNT5A\|WNT7A |
| Circadian entrainment | chx04713 | 12 | 100 | 0.007739 | 0.043823 | ADCY5\|PLCB2\|GRIN2C\|ADCY2\|FOS\|PRKCA\|PRKCB\|CACNA1C\|CACNA1G\|PER2\|GNB3\|GRIN1 |
| Malaria | chx05144 | 8 | 52 | 0.007826 | 0.043823 | TGFB3\|IL10\|THBS4\|ITGB2\|THBS1\|HBBC\|ITGAL\|CD36 |
| Oxytocin signaling pathway | chx04921 | 16 | 155 | 0.008619 | 0.047404 | CD38\|NFATC1\|PLCB2\|CACNA1C\|ADCY2\|PTGS2\|PRKCA\|ADCY5\|FOS\|PRKCB\|CACNB2\|CAMKK2\|PPP1R12B\|PPP1R12C\|PLA2G4F\|PLA2G4E |
| Cushing syndrome | chx04934 | 16 | 156 | 0.009091 | 0.049126 | ADCY5\|PLCB2\|CACNA1C\|ADCY2\|CACNA1G\|CREB5\|NR4A1\|WNT4\|CREB3L3\|WNT5B\|WNT5A\|KCNK3\|WNT7A\|WNT10B\|PDE8B\|CRHR1 |

## Table S12. Spearman’s correlation coefficient and *p* value of each host module with age.

| **ID** | **Number of genes** | **Module** | **Correlation** | ***P* value** |
| --- | --- | --- | --- | --- |
| R1 | 211 | MEbrown | 0.59 | 0.005 |
| R2 | 104 | MEpink | 0.51 | 0.02 |
| R3 | 67 | MEgreenyellow | 0.46 | 0.04 |
| R4 | 417 | MEturquoise | 0.87 | 4.00E-07 |
| R5 | 136 | MEred | -0.61 | 0.003 |
| R6 | 180 | MEyellow | -0.0026 | 0.9 |
| R7 | 73 | MEmagenta | -0.0023 | 1 |
| R8 | 174 | MEgreen | 0.43 | 0.05 |
| R9 | 63 | MEsalmon | 0.49 | 0.02 |
| R10 | 46 | MEmidnightblue | 0.063 | 0.8 |
| R11 | 47 | MEbcyan | 0.42 | 0.06 |
| R12 | 64 | MEtan | 0.36 | 1 |
| R13 | 68 | MEpurple | -0.075 | 0.7 |
| R14 | 113 | MEblack | -0.4 | 0.07 |
| R15 | 257 | MEblue | -0.79 | 2.00E-05 |

## Table S13 Statistical data for the metagenomics raw data.

| **Sample ID** | **Number of clean reads** | **Total clean reads number of bases (bp)** | **Number of raw reads** | **Total raw reads number of bases (bp)** | **SRA_ID** |
| --- | --- | --- | --- | --- | --- |
| d 7-1 | 47,137,847 | 6,036,440,799 | 58,068,965 | 8,710,344,702 | SRR7755414 |
| d 7-3 | 58,802,813 | 6,640,419,436 | 66,805,613 | 10,020,841,992 | SRR7755417 |
| d 14-1 | 41,173,880 | 4,913,772,149 | 51,523,825 | 7,728,573,808 | SRR7755428 |
| d 14-2 | 44,656,394 | 5,209,154,509 | 55,170,433 | 8,275,564,966 | SRR7755419 |
| d 14-3 | 45,495,146 | 5,227,350,653 | 55,998,733 | 8,399,809,880 | SRR7755418 |
| d 21-1 | 42,907,466 | 5,035,565,977 | 53,090,938 | 7,963,640,642 | SRR7755430 |
| d 21-2 | 42,829,928 | 5,764,383,388 | 50,375,100 | 7,556,264,990 | SRR7755429 |
| d 21-3 | 50,145,262 | 6,676,745,250 | 57,554,645 | 8,633,196,688 | SRR7755276 |
| d 28-1 | 41,749,677 | 4,851,754,541 | 51,837,501 | 7,775,625,106 | SRR7755277 |
| d 28-2 | 52,110,384 | 6,918,083,846 | 59,769,154 | 8,965,373,132 | SRR7755274 |
| d 28-3 | 49,661,323 | 6,408,694,002 | 56,497,194 | 8,474,579,040 | SRR7755275 |
| d 42-1 | 42,759,897 | 4,748,436,279 | 52,493,636 | 7,874,045,396 | SRR7755280 |
| d 42-2 | 47,800,153 | 6,314,800,770 | 54,870,573 | 8,230,585,992 | SRR7755281 |
| d 42-3 | 47,054,893 | 6,242,304,527 | 54,013,848 | 8,102,077,140 | SRR7755078 |
| d 56-1 | 47,577,200 | 6,278,998,552 | 54,550,528 | 8,182,579,166 | SRR7755105 |
| d 56-2 | 35,601,553 | 3,724,775,116 | 43,218,388 | 6,482,758,274 | SRR7755116 |
| d 56-3 | 38,900,493 | 4,615,024,729 | 47,962,825 | 7,194,423,690 | SRR7755431 |
| **Sum** | **776,364,309** | **95,606,704,523** | **923,801,897** | **138,570,284,604** | **-** |
| **Average** | **45,668,489** | **5,623,923,795** | **54,341,288** | **8,151,193,212** | **-** |
| **SD** | **5,258,962** | **877,103,845** | **4,951,973** | **742,795,950** | **-** |

## Table S14. Statistical data for metagenomics assembly (contig-level).

| **Sample ID** | **Number of contigs** | **Total contig length** | **Max contig length** | **Contig N50** | **Host mapping rate** |
| --- | --- | --- | --- | --- | --- |
| d 7-1 | 20,702 | 50,723,535 | 142,702 | 5,762 | 9.78% |
| d 7-3 | 27,426 | 66,471,685 | 140,571 | 6,212 | 21.19% |
| d 14-1 | 22,340 | 45,190,413 | 148,620 | 3,775 | 10.39% |
| d 14-2 | 39,648 | 60,227,899 | 51,123 | 2,150 | 12.86% |
| d 14-3 | 24,954 | 48,076,694 | 96,013 | 3,665 | 14.41% |
| d 21-1 | 43,421 | 59,822,055 | 52,587 | 1,762 | 12.27% |
| d 21-2 | 27,465 | 55,108,393 | 118,402 | 4,177 | 5.39% |
| d 21-3 | 38,853 | 69,927,538 | 79,646 | 2,778 | 6.76% |
| d 28-1 | 42,572 | 70,403,108 | 96,080 | 2,626 | 12.99% |
| d 28-2 | 38,424 | 54,699,352 | 69,146 | 1,799 | 7.12% |
| d 28-3 | 50,068 | 78,864,757 | 122,251 | 2,442 | 9.72% |
| d 42-1 | 30,022 | 45,018,879 | 73,581 | 2,226 | 17.39% |
| d 42-2 | 54,261 | 83,232,123 | 69,167 | 2,127 | 7.68% |
| d 42-3 | 43,006 | 61,693,849 | 82,349 | 1,886 | 7.44% |
| d 56-1 | 64,898 | 83,462,278 | 70,471 | 1,464 | 7.82% |
| d 56-2 | 27,253 | 29,671,520 | 50,390 | 1,155 | 20.22% |
| d 56-3 | 44,328 | 49,230,675 | 45,869 | 1,171 | 11.55% |
| **Sum** | **639,641** | **1,011,824,753** | **-** | **-** | **-** |
| **Average** | **37,626** | **59,519,103** | **88,763** | **2,775** | **11%** |
| **SD** | **11,799** | **14,253,110** | **33,236** | **1,444** | **5%** |

## Table S15. Statistical data for metagenomics assembly (scaffold-level).

| **Sample ID** | **Number of scaffold** | **Total scaffold length** | **Max scaffold length** | **Scaffold N50** | **Average length** |
| --- | --- | --- | --- | --- | --- |
| d 7-1 | 13,991 | 55,974,824 | 263,696 | 18,197 | 4,000 |
| d 7-3 | 20,988 | 75,565,566 | 429,658 | 12,729 | 3,600 |
| d 14-1 | 17,349 | 51,789,597 | 264,408 | 8,911 | 2,985 |
| d 14-2 | 31,959 | 74,310,892 | 245,786 | 4,484 | 2,325 |
| d 14-3 | 20,229 | 55,608,185 | 254,023 | 6,621 | 2,748 |
| d 21-1 | 36,828 | 76,405,276 | 235,483 | 3,494 | 2,074 |
| d 21-2 | 24,454 | 67,023,624 | 406,502 | 10,033 | 2,740 |
| d 21-3 | 24,361 | 81,664,983 | 214,328 | 10,751 | 3,352 |
| d 28-1 | 34,940 | 85,096,215 | 172,341 | 5,593 | 2,435 |
| d 28-2 | 29,673 | 69,787,833 | 242,658 | 4,609 | 2,351 |
| d 28-3 | 42,051 | 101,261,148 | 403,494 | 6,177 | 2,408 |
| d 42-1 | 27,180 | 57,019,017 | 428,145 | 3,930 | 2,097 |
| d 42-2 | 39,116 | 103,749,485 | 174,317 | 6,327 | 2,652 |
| d 42-3 | 34,937 | 79,915,056 | 468,543 | 5,074 | 2,287 |
| d 56-1 | 51,599 | 112,930,859 | 125,993 | 3,709 | 2,188 |
| d 56-2 | 25,811 | 40,331,946 | 200,565 | 2,048 | 1,562 |
| d 56-3 | 46,292 | 66,891,748 | 45,869 | 1,815 | 1,444 |
| **Sum** | **521,758** | **1,255,326,254** | **-** | **-** | **43,248** |
| **Average** | **30,692** | **73,842,721** | **269,165** | **6,735** | **2,544** |
| **SD** | **10,095** | **18,886,489** | **115,504** | **4,108** | **645** |

## Table S16. Statistical data for non-redundant gene catalog annotation.

| **ORF No.** | **Integrity:all** | **Integrity:start** | **Integrity:end** | **Integrity:none** | **Total Len. (Mbp)** | **Average Len. (bp)** | **GC percent** |
| --- | --- | --- | --- | --- | --- | --- | --- |
| 1,320,084 | 723,645 (54.82%) | 230,382 (17.45%) | 286,104 (21.67%) | 79,953 (6.05%) | 954.30 | 723 | 51.67% |

## Table S17. The relative abundance of KEGG pathway abundance (Mean ± SEM) at the third level during the early rumen development.

| **taxa** | **d 7** | **d 14** | **d 21** | **d 28** | **d 42** | **d 56** | **level-1** | **level-2** | **level-3** |
| --- | --- | --- | --- | --- | --- | --- | --- | --- | --- |
| ko00010 | 4.599235954±0.210441452 | 4.262197124±0.335722435 | 3.991320582±0.152621126 | 3.827133627±0.064342788 | 3.318531585±0.037942996 | 3.257281354±0.391703082 | Metabolism | Carbohydrate Metabolism | Glycolysis / Gluconeogenesis |
| ko00020 | 1.791704588±0.059622677 | 1.553188357±0.092451023 | 1.423634624±0.109030559 | 1.471286505±0.068618158 | 1.261857894±0.104084986 | 1.147012338±0.354104388 | Metabolism | Carbohydrate Metabolism | Citrate cycle (TCA cycle) |
| ko00030 | 2.45632553±0.142069672 | 2.875643642±0.123790361 | 2.746885932±0.125599947 | 2.682171058±0.030977361 | 1.906246757±0.094219084 | 2.123705659±0.244931526 | Metabolism | Carbohydrate Metabolism | Pentose phosphate pathway |
| ko00040 | 0.940564107±0.149345915 | 0.869743567±0.160912465 | 0.992222923±0.0635526 | 0.983665493±0.196856504 | 1.511681621±0.217035406 | 1.125398474±0.302974048 | Metabolism | Carbohydrate Metabolism | Pentose and glucuronate interconversions |
| ko00051 | 12.14673558±1.053664573 | 12.20390117±1.571367583 | 16.31203067±0.578771599 | 15.87771343±2.129483231 | 20.54516558±0.840466322 | 15.2075272±2.079024664 | Metabolism | Carbohydrate Metabolism | Fructose and mannose metabolism |
| ko00052 | 7.8421166±1.321219649 | 8.26944928±0.436308155 | 11.1514794±0.551963994 | 9.719140041±1.352922883 | 13.06894764±0.959149127 | 9.623783021±2.840426188 | Metabolism | Carbohydrate Metabolism | Galactose metabolism |
| ko00053 | 0.177010237±0.020263833 | 0.166340846±0.013021564 | 0.191664276±0.024299587 | 0.17965079±0.048686941 | 0.214093737±0.013614183 | 0.154213876±0.027224991 | Metabolism | Carbohydrate Metabolism | Ascorbate and aldarate metabolism |
| ko00061 | 1.620816378±0.260748852 | 1.278551035±0.088621946 | 2.294421761±0.186961326 | 2.023009528±0.805176374 | 1.613446799±0.134962532 | 1.630325287±0.048775812 | Metabolism | Lipid Metabolism | Fatty acid biosynthesis |
| ko00071 | 2.129186345±0.123541568 | 2.05972803±0.35820386 | 3.07687179±0.437439923 | 2.45464732±0.269781042 | 2.239253808±0.099543725 | 2.027114767±0.509280539 | Metabolism | Lipid Metabolism | Fatty acid metabolism |
| ko00072 | 0.036428474±0.015782008 | 0.034190538±0.009482422 | 0.014292576±0.00485627 | 0.016257268±0.007059267 | 0.002646137±0.002242803 | 0.007849144±0.002003148 | Metabolism | Lipid Metabolism | Synthesis and degradation of ketone bodies |
| ko00100 | 5.34E-05±2.85958E-05 | 1.91E-05±1.75522E-05 | 0.000400429±0.000386727 | 0.000208037±7.23352E-05 | 7.64852E-05±7.48907E-05 | 3.49751E-05±3.75339E-05 | Metabolism | Lipid Metabolism | Steroid biosynthesis |
| ko00120 | 0.000229093±0.000221573 | 0.000282347±0.000130705 | 0.001190434±0.000767299 | 0.000790419±0.00053311 | 0.002290669±0.001666647 | 0.001427389±0.000493127 | Metabolism | Lipid Metabolism | Primary bile acid biosynthesis |
| ko00121 | 0.000162762±0.000162762 | 1.42E-04±0.000150452 | 0.00098967±0.000797295 | 0.000540614±0.000431891 | 0.002139338±0.0016244 | 0.000962926±0.00058068 | Metabolism | Lipid Metabolism | Secondary bile acid biosynthesis |
| ko00130 | 0.366582195±0.001257348 | 0.255234472±0.016535732 | 0.215403303±0.024275824 | 0.223055041±0.045942078 | 0.283364311±0.01579636 | 0.227247967±0.021537864 | Metabolism | Metabolism of Cofactors and Vitamins | Ubiquinone and other terpenoid-quinone biosynthesis |
| ko00140 | 0.044356279±0.003916787 | 0.035491496±0.008082605 | 0.041872572±0.027169994 | 0.034906718±0.009725455 | 0.049621832±0.029213485 | 0.03248437±0.017990644 | Metabolism | Lipid Metabolism | Steroid hormone biosynthesis |
| ko00190 | 2.356659067±0.181045274 | 2.405788264±0.102694355 | 2.420413375±0.336977477 | 2.354939868±0.121276556 | 1.793537107±0.071419183 | 1.938776644±0.374447997 | Metabolism | Energy Metabolism | Oxidative phosphorylation |
| ko00194 | 0.572031188±0.090701009 | 0.664574267±0.099124393 | 0.673981012±0.162045624 | 0.623126009±0.047495723 | 0.555706248±0.023558054 | 0.57157222±0.068669842 | Metabolism | Energy Metabolism | Photosynthesis proteins |
| ko00195 | 0.567319848±0.09154459 | 0.659831578±0.099728952 | 0.671087549±0.161748425 | 0.620637613±0.046257027 | 0.54960054±0.026482872 | 0.567742205±0.066205026 | Metabolism | Energy Metabolism | Photosynthesis |
| ko00230 | 11.91718471±0.825003674 | 11.31960462±0.583494302 | 13.17471909±0.554914321 | 13.03213228±0.781701405 | 11.21879014±0.152651529 | 12.11977092±1.163694466 | Metabolism | Nucleotide Metabolism | Purine metabolism |
| ko00232 | 0±0 | 0±0 | 4.59921E-06±6.50427E-06 | 2.04992E-06±2.89902E-06 | 8.25E-07±1.16644E-06 | 0±0 | Metabolism | Biosynthesis of Other Secondary Metabolites | Caffeine metabolism |
| ko00240 | 11.1294748±0.784357813 | 10.57630237±0.422852276 | 12.19166198±0.449293556 | 12.14548735±0.759180831 | 11.28112721±0.428021106 | 11.61349868±0.67721183 | Metabolism | Nucleotide Metabolism | Pyrimidine metabolism |
| ko00250 | 5.105835734±0.160202104 | 4.829231407±0.181116537 | 6.442653924±0.416139343 | 5.957113588±0.44540435 | 7.395905951±0.306906343 | 6.624090656±1.222812311 | Metabolism | Amino Acid Metabolism | Alanine, aspartate and glutamate metabolism |
| ko00253 | 0.039915067±0.023600056 | 0.011304909±0.008306261 | 0.015422381±0.007644664 | 0.023173907±0.018757832 | 0.011109953±0.007617065 | 0.027876448±0.015405163 | Metabolism | Metabolism of Terpenoids and Polyketides | Tetracycline biosynthesis |
| ko00260 | 3.037823616±0.10837619 | 2.912672296±0.079573086 | 2.626797962±0.133685267 | 2.799996464±0.237150776 | 2.069356368±0.062118176 | 2.256151433±0.219639489 | Metabolism | Amino Acid Metabolism | Glycine, serine and threonine metabolism |
| ko00270 | 3.703885452±0.058702727 | 3.8182733±0.246499615 | 3.054260161±0.151574477 | 3.431405906±0.262481667 | 3.532584856±0.21774474 | 3.540467121±0.337178463 | Metabolism | Amino Acid Metabolism | Cysteine and methionine metabolism |
| ko00280 | 0.931318181±0.018657702 | 0.854845206±0.045079083 | 0.838169703±0.141925075 | 0.78821793±0.063714395 | 0.589252868±0.047723606 | 0.610360867±0.18893824 | Metabolism | Amino Acid Metabolism | Valine, leucine and isoleucine degradation |
| ko00281 | 0.239297627±0.011780292 | 0.180296073±0.009217979 | 0.227683442±0.031411464 | 0.185422559±0.049109987 | 0.170219404±0.024321115 | 0.120010944±0.013273223 | Metabolism | Metabolism of Terpenoids and Polyketides | Geraniol degradation |
| ko00290 | 2.542940171±0.197343562 | 2.228473417±0.107666231 | 2.660911359±0.118844423 | 2.675678803±0.420476717 | 2.442196426±0.107429072 | 2.563851578±0.278085173 | Metabolism | Amino Acid Metabolism | Valine, leucine and isoleucine biosynthesis |
| ko00300 | 2.050147054±0.125929662 | 2.098727645±0.104317963 | 1.794710102±0.019413191 | 1.946395282±0.15477087 | 1.77557252±0.016454767 | 1.891961511±0.196756747 | Metabolism | Amino Acid Metabolism | Lysine biosynthesis |
| ko00310 | 0.657314475±0.000248536 | 0.617919778±0.020441662 | 0.80944836±0.091864474 | 0.682794478±0.121689536 | 0.410768421±0.039478381 | 0.341096371±0.006942148 | Metabolism | Amino Acid Metabolism | Lysine degradation |
| ko00311 | 0.058708402±0.013496213 | 0.093095586±0.025413804 | 0.193745333±0.016473084 | 0.131455717±0.022600862 | 0.110052748±0.020275926 | 0.132844772±0.034440783 | Metabolism | Biosynthesis of Other Secondary Metabolites | Penicillin and cephalosporin biosynthesis |
| ko00312 | 0.024494179±0.003422018 | 0.065983565±0.034732869 | 0.158086626±0.020539238 | 0.11551304±0.014327638 | 0.075368388±0.024630501 | 0.111523224±0.042038401 | Metabolism | Biosynthesis of Other Secondary Metabolites | beta-Lactam resistance |
| ko00330 | 3.418364043±0.132874381 | 3.097370365±0.049564888 | 3.703795725±0.147653407 | 3.505900885±0.09610616 | 4.123261326±0.224294759 | 3.636249878±0.64264362 | Metabolism | Amino Acid Metabolism | Arginine and proline metabolism |
| ko00340 | 6.377828946±0.122644146 | 5.807586825±0.142335987 | 5.997076677±0.677323319 | 5.682065885±0.178529467 | 5.697829249±0.203962885 | 5.665677489±0.342317975 | Metabolism | Amino Acid Metabolism | Histidine metabolism |
| ko00350 | 7.441489192±0.399910418 | 7.683672015±0.386034542 | 7.730338883±0.629433038 | 7.928073701±0.5420974 | 7.892028786±0.507190579 | 7.239022522±0.578033349 | Metabolism | Amino Acid Metabolism | Tyrosine metabolism |
| ko00351 | 5.67E-05±2.83124E-05 | 2.84E-05±2.81794E-05 | 6.77239E-05±3.39762E-05 | 4.54603E-05±2.1807E-05 | 5.4732E-06±7.74028E-06 | 6.04656E-05±6.16469E-05 | Metabolism | Xenobiotics Biodegradation and Metabolism | 1,1,1-Trichloro-2,2-bis(4-chlorophenyl)ethane (DDT) degradation |
| ko00360 | 0.618976658±0.059253792 | 0.60183722±0.027455108 | 0.706604504±0.082056592 | 0.65678342±0.091614225 | 0.760001501±0.149249901 | 0.631452955±0.182347291 | Metabolism | Amino Acid Metabolism | Phenylalanine metabolism |
| ko00361 | 0.370027957±0.041398328 | 0.268802435±0.053645014 | 0.328610948±0.027035465 | 0.298333376±0.056723703 | 0.302865444±0.047451365 | 0.286976566±0.022329495 | Metabolism | Xenobiotics Biodegradation and Metabolism | gamma-Hexachlorocyclohexane degradation |
| ko00362 | 0.462090429±0.070037735 | 0.44458634±0.021732356 | 0.330785867±0.027044253 | 0.276047058±0.116269418 | 0.301467358±0.022018264 | 0.328781998±0.064199718 | Metabolism | Xenobiotics Biodegradation and Metabolism | Benzoate degradation via hydroxylation |
| ko00363 | 1.95659171±0.066422206 | 2.574373176±0.187113974 | 3.05921751±0.199182859 | 2.853658221±0.081875635 | 2.249622837±0.179574285 | 2.37234322±0.275511222 | Metabolism | Xenobiotics Biodegradation and Metabolism | Bisphenol A degradation |
| ko00364 | 0.008753774±0.001366391 | 0.005314979±0.001673654 | 0.005993758±0.003193656 | 0.006235316±0.002743863 | 0.004587617±0.001155887 | 0.004231318±0.002441922 | Metabolism | Xenobiotics Biodegradation and Metabolism | Fluorobenzoate degradation |
| ko00380 | 0.307744335±0.048935846 | 0.2689923±0.002970603 | 0.207598234±0.022788711 | 0.230603549±0.018371993 | 0.174789947±0.018472536 | 0.191451924±0.034137693 | Metabolism | Amino Acid Metabolism | Tryptophan metabolism |
| ko00400 | 1.688145323±0.019299776 | 1.479633431±0.091635246 | 1.652741618±0.178503155 | 1.644008818±0.196980361 | 1.832701116±0.056987021 | 1.515352618±0.203872096 | Metabolism | Amino Acid Metabolism | Phenylalanine, tyrosine and tryptophan biosynthesis |
| ko00401 | 0.402625765±0.01973088 | 0.36978823±0.023814163 | 0.36063511±0.023578303 | 0.349824006±0.021016963 | 0.47236556±0.057155157 | 0.348247927±0.081089552 | Metabolism | Biosynthesis of Other Secondary Metabolites | Novobiocin biosynthesis |
| ko00410 | 0.59975675±0.023078324 | 0.507569384±0.08173086 | 0.593928137±0.119500835 | 0.483961406±0.042992113 | 0.600213347±0.05031377 | 0.492989067±0.044934677 | Metabolism | Metabolism of Other Amino Acids | beta-Alanine metabolism |
| ko00430 | 0.493096998±0.03963085 | 0.417679333±0.008121706 | 0.385369957±0.022896679 | 0.382905624±0.042890637 | 0.317040952±0.04828866 | 0.294546672±0.088295579 | Metabolism | Metabolism of Other Amino Acids | Taurine and hypotaurine metabolism |
| ko00440 | 0.234571234±0.053883728 | 0.181769896±0.006194731 | 0.265397944±0.041383137 | 0.228142199±0.051755195 | 0.354837456±0.03414711 | 0.273706252±0.050297032 | Metabolism | Metabolism of Other Amino Acids | Phosphonate and phosphinate metabolism |
| ko00450 | 6.343604189±0.215689389 | 5.946202187±0.116076735 | 5.82007334±0.574020378 | 5.667258847±0.265671949 | 5.517828859±0.213230187 | 5.821838106±0.270986939 | Metabolism | Metabolism of Other Amino Acids | Selenoamino acid metabolism |
| ko00460 | 2.027774386±0.29189517 | 1.645512669±0.204579748 | 2.978669565±0.407488696 | 2.64797595±0.643887945 | 4.699802395±0.450539421 | 3.147133428±1.452188452 | Metabolism | Metabolism of Other Amino Acids | Cyanoamino acid metabolism |
| ko00471 | 0.712127526±0.062949315 | 0.657881427±0.018410274 | 0.696183948±0.062081869 | 0.692193577±0.080022675 | 0.554771374±0.030879049 | 0.598392786±0.032894536 | Metabolism | Metabolism of Other Amino Acids | D-Glutamine and D-glutamate metabolism |
| ko00472 | 0.000405007±0.000332475 | 2.80E-04±0.000259941 | 0.000358624±0.000137444 | 0.0007209±0.000422297 | 0.000257402±0.00014442 | 0.000730297±0.000369568 | Metabolism | Metabolism of Other Amino Acids | D-Arginine and D-ornithine metabolism |
| ko00473 | 0.589769253±0.037871156 | 0.548422303±0.024273045 | 0.591607037±0.075310476 | 0.613852967±0.054729967 | 0.482181093±0.013729667 | 0.481778829±0.103931197 | Metabolism | Metabolism of Other Amino Acids | D-Alanine metabolism |
| ko00480 | 0.717591081±0.047431696 | 0.636732265±0.07647487 | 0.764076946±0.115927434 | 0.675300258±0.042756406 | 0.788046499±0.021809509 | 0.649094033±0.054789652 | Metabolism | Metabolism of Other Amino Acids | Glutathione metabolism |
| ko00500 | 7.817596751±0.84143726 | 6.924392081±0.413729335 | 9.796908792±0.99845655 | 8.628806006±1.59497646 | 13.34783938±0.47465558 | 10.32850706±2.851582577 | Metabolism | Carbohydrate Metabolism | Starch and sucrose metabolism |
| ko00510 | 0.170870265±0.04179133 | 0.296292676±0.064509171 | 0.312827141±0.078657774 | 0.35220356±0.092049703 | 0.247849914±0.011506092 | 0.246769215±0.011818968 | Metabolism | Glycan Biosynthesis and Metabolism | N-Glycan biosynthesis |
| ko00511 | 5.216314559±1.008551743 | 5.827808779±0.5883862 | 7.797973305±0.917609298 | 6.974444442±0.98552321 | 8.31742242±0.799777149 | 6.068170477±2.020559956 | Metabolism | Glycan Biosynthesis and Metabolism | Other glycan degradation |
| ko00512 | 0±0 | 0±0 | 0±0 | 0±0 | 0±0 | 0±0 | Metabolism | Glycan Biosynthesis and Metabolism | O-Glycan biosynthesis |
| ko00513 | 3.42E-06±3.41981E-06 | 0±0 | 2.25788E-07±3.19312E-07 | 0±0 | 8.61156E-05±6.10135E-05 | 2.88576E-06±4.08108E-06 | Metabolism | Glycan Biosynthesis and Metabolism | High-mannose type N-glycan biosynthesis |
| ko00514 | 0.000587278±9.12134E-05 | 0.00012628±0.000100982 | 0.000409933±0.00010622 | 0.00138031±0.000856321 | 0.000884794±0.000266236 | 0.000892555±0.000632128 | Metabolism | Glycan Biosynthesis and Metabolism | O-Mannosyl glycan biosynthesis |
| ko00520 | 8.316533506±0.442913939 | 8.312319139±0.546745694 | 8.546851703±0.321174095 | 8.4365531±0.317342414 | 8.820608267±0.599800626 | 7.45399227±1.854884188 | Metabolism | Carbohydrate Metabolism | Amino sugar and nucleotide sugar metabolism |
| ko00521 | 1.919075713±0.078315439 | 1.520655121±0.139000228 | 1.701411066±0.086112791 | 1.746355043±0.040793982 | 1.826939444±0.077974733 | 1.649405794±0.12764779 | Metabolism | Biosynthesis of Other Secondary Metabolites | Streptomycin biosynthesis |
| ko00522 | 0±0 | 3.60176E-06±5.09366E-06 | 9.75E-04±0.001233253 | 1.52E-03±0.002145581 | 0±0 | 7.38697E-06±1.04468E-05 | Metabolism | Metabolism of Terpenoids and Polyketides | Biosynthesis of 12-, 14- and 16-membered macrolides |
| ko00523 | 0.978921679±0.035347747 | 0.776294464±0.083362804 | 1.051329772±0.163788683 | 1.128555097±0.210599021 | 1.040961558±0.059482349 | 1.035784779±0.104955993 | Metabolism | Metabolism of Terpenoids and Polyketides | Polyketide sugar unit biosynthesis |
| ko00524 | 0.75838839±0.060537691 | 0.561568209±0.125601342 | 0.554309612±0.065604994 | 0.473848029±0.119333636 | 0.62973765±0.027987688 | 0.47652213±0.13532545 | Metabolism | Biosynthesis of Other Secondary Metabolites | Butirosin and neomycin biosynthesis |
| ko00531 | 1.215023231±0.16032535 | 1.12447325±0.272792734 | 1.179570405±0.263900003 | 1.125357696±0.062086117 | 0.734203847±0.177412359 | 0.607689666±0.229344747 | Metabolism | Glycan Biosynthesis and Metabolism | Glycosaminoglycan degradation |
| ko00532 | 0.000001356±1.356E-06 | 1.42381E-07±2.01357E-07 | 0±0 | 0±0 | 7.36E-06±3.56634E-06 | 4.06342E-05±5.11461E-05 | Metabolism | Glycan Biosynthesis and Metabolism | Glycosaminoglycan biosynthesis - chondroitin sulfate |
| ko00535 | 1.03E-05±1.02913E-05 | 0.000829835±0.000635307 | 0.000160402±7.39355E-05 | 0.000204029±0.000177207 | 0.000164681±0.000133549 | 9.92E-05±5.1294E-05 | Metabolism | Glycan Biosynthesis and Metabolism | Proteoglycans |
| ko00540 | 0.914099572±0.074468649 | 0.664303314±0.116492278 | 0.977902995±0.167759954 | 0.927838598±0.301608749 | 0.938482404±0.051372735 | 0.864999414±0.159188534 | Metabolism | Glycan Biosynthesis and Metabolism | Lipopolysaccharide biosynthesis |
| ko00550 | 4.11711979±0.21804645 | 4.129825136±0.084549892 | 3.915456584±0.113561974 | 4.020609999±0.291818181 | 3.750131133±0.075848516 | 3.97028929±0.260897377 | Metabolism | Glycan Biosynthesis and Metabolism | Peptidoglycan biosynthesis |
| ko00561 | 0.61564962±0.033081961 | 0.666705226±0.082806949 | 1.058754445±0.021967524 | 0.954050462±0.154353706 | 0.807148723±0.036269586 | 0.806488886±0.250533662 | Metabolism | Lipid Metabolism | Glycerolipid metabolism |
| ko00562 | 0.343511112±0.016261152 | 0.332096658±0.043364787 | 0.226405419±0.002343025 | 0.274499008±0.038990396 | 0.268580393±0.011323893 | 0.198669946±0.02041928 | Metabolism | Carbohydrate Metabolism | Inositol phosphate metabolism |
| ko00563 | 0.001530139±0.000168475 | 0.001150187±0.000182871 | 0.000795638±0.000367118 | 0.001954288±0.00092437 | 0.000589926±0.00042721 | 2.03E-04±0.000132604 | Metabolism | Glycan Biosynthesis and Metabolism | Glycosylphosphatidylinositol(GPI)-anchor biosynthesis |
| ko00564 | 1.228324995±0.146627096 | 1.100792064±0.08902055 | 1.207152996±0.033994093 | 1.170214464±0.098492274 | 0.83118629±0.047836126 | 0.832314243±0.094066325 | Metabolism | Lipid Metabolism | Glycerophospholipid metabolism |
| ko00565 | 0.000680252±2.09784E-05 | 0.00091386±0.000336688 | 0.002349428±0.001061335 | 0.001932021±0.001416099 | 2.72E-04±0.000213717 | 2.41E-04±0.000248934 | Metabolism | Lipid Metabolism | Ether lipid metabolism |
| ko00590 | 0.109221063±0.033459571 | 0.124352376±0.017658062 | 0.157947325±0.010294377 | 0.169192956±0.010378884 | 0.177175825±0.032615668 | 0.141020927±0.023328453 | Metabolism | Lipid Metabolism | Arachidonic acid metabolism |
| ko00591 | 1.878180952±0.062919446 | 2.522078998±0.194016327 | 3.029737561±0.208817463 | 2.82828369±0.072411656 | 2.186835134±0.178281942 | 2.313289777±0.269846282 | Metabolism | Lipid Metabolism | Linoleic acid metabolism |
| ko00592 | 0.001478549±7.07605E-05 | 0.000773979±0.000923294 | 0.000546686±0.000421565 | 0.001963208±0.002149854 | 0.001785802±0.000701501 | 0.000543241±0.000446725 | Metabolism | Lipid Metabolism | alpha-Linolenic acid metabolism |
| ko00600 | 3.86018967±0.781585039 | 4.676840355±0.170125739 | 6.590289751±0.73396514 | 5.76262673±1.049953766 | 7.744244385±0.746698632 | 5.614734646±1.837910424 | Metabolism | Lipid Metabolism | Sphingolipid metabolism |
| ko00601 | 0.000146363±5.94642E-05 | 0.001031207±0.001041132 | 0.002288243±0.00122132 | 0.001919758±0.00198936 | 0.000847706±0.000337032 | 0.000877943±0.00060259 | Metabolism | Glycan Biosynthesis and Metabolism | Glycosphingolipid biosynthesis - lacto and neolacto series |
| ko00603 | 1.279027471±0.175669078 | 1.333111476±0.230154378 | 1.621802691±0.293837375 | 1.497330262±0.209987143 | 1.128282496±0.234592125 | 0.9851332±0.399988656 | Metabolism | Glycan Biosynthesis and Metabolism | Glycosphingolipid biosynthesis - globo series |
| ko00604 | 1.07532614±0.146326787 | 1.029671395±0.271165148 | 1.040318556±0.270922165 | 0.997397125±0.049134706 | 0.588905572±0.173790564 | 0.511396676±0.204436389 | Metabolism | Glycan Biosynthesis and Metabolism | Glycosphingolipid biosynthesis - ganglio series |
| ko00620 | 2.665962875±0.183259255 | 2.41163106±0.048900777 | 2.292402067±0.139798123 | 2.376626403±0.216623506 | 1.759056211±0.081592731 | 1.76120079±0.450976434 | Metabolism | Carbohydrate Metabolism | Pyruvate metabolism |
| ko00621 | 0.006581512±0.001633909 | 0.008404634±0.00120885 | 0.007352566±0.002612994 | 0.010115213±0.003959952 | 0.009305927±0.002085859 | 0.012525271±0.001131264 | Metabolism | Xenobiotics Biodegradation and Metabolism | Biphenyl degradation |
| ko00622 | 0.007652573±0.00217321 | 0.00882571±0.001192294 | 0.00757819±0.002712732 | 0.010330454±0.003923746 | 0.009462742±0.001966495 | 0.012627176±0.001184621 | Metabolism | Xenobiotics Biodegradation and Metabolism | Toluene and xylene degradation |
| ko00623 | 0.029830808±0.005332906 | 0.02940131±0.016384524 | 0.03241145±0.009709463 | 0.039973±0.001267155 | 0.040412734±0.014318901 | 0.060918481±0.011711565 | Metabolism | Xenobiotics Biodegradation and Metabolism | 2,4-Dichlorobenzoate degradation |
| ko00624 | 2.869971957±0.331138147 | 3.426056072±0.353397248 | 3.404448881±0.281646322 | 3.680128625±0.226951979 | 3.499671975±0.288752827 | 2.925056517±0.181812546 | Metabolism | Xenobiotics Biodegradation and Metabolism | 1- and 2-Methylnaphthalene degradation |
| ko00625 | 1.879550689±0.063859268 | 2.523318323±0.194516558 | 3.030365054±0.208509492 | 2.831561046±0.074973971 | 2.189724451±0.176457806 | 2.322070344±0.272530471 | Metabolism | Xenobiotics Biodegradation and Metabolism | Tetrachloroethene degradation |
| ko00626 | 5.01840716±0.14283076 | 4.603154999±0.118711672 | 4.579558847±0.551531399 | 4.405297354±0.177488991 | 4.507003106±0.183384401 | 4.523586321±0.323974936 | Metabolism | Xenobiotics Biodegradation and Metabolism | Naphthalene and anthracene degradation |
| ko00627 | 0.016370481±0.000828411 | 0.013861338±0.001403866 | 0.013468822±0.0018621 | 0.016552568±0.002949941 | 0.014007753±0.003176822 | 0.016831001±0.001504005 | Metabolism | Xenobiotics Biodegradation and Metabolism | 1,4-Dichlorobenzene degradation |
| ko00628 | 0.006574142±0.001651004 | 0.008488077±0.001239777 | 0.007379021±0.002594181 | 0.010118462±0.003969431 | 0.009304623±0.00208141 | 0.01260062±0.001213052 | Metabolism | Xenobiotics Biodegradation and Metabolism | Fluorene degradation |
| ko00629 | 0.006537036±0.001627154 | 0.008400334±0.001207838 | 0.007352566±0.002612994 | 0.010108271±0.003969447 | 0.009296647±0.002080216 | 0.012508058±0.001107414 | Metabolism | Xenobiotics Biodegradation and Metabolism | Carbazole degradation |
| ko00630 | 1.303368609±0.038410513 | 1.330390568±0.147081012 | 1.153285834±0.073442071 | 1.234909431±0.13178499 | 0.976393563±0.024372302 | 0.999477648±0.19698461 | Metabolism | Carbohydrate Metabolism | Glyoxylate and dicarboxylate metabolism |
| ko00631 | 0.011025±0.00317322 | 0.013769581±0.004344109 | 0.01500527±0.010182615 | 0.011553833±0.005249614 | 0.002160979±0.000694124 | 0.014897603±0.004363929 | Metabolism | Xenobiotics Biodegradation and Metabolism | 1,2-Dichloroethane degradation |
| ko00632 | 3.009530345±0.354133809 | 3.577001475±0.304834391 | 3.438036417±0.273209208 | 3.745668261±0.398175363 | 3.568201353±0.285080884 | 3.057397418±0.133434475 | Metabolism | Xenobiotics Biodegradation and Metabolism | Benzoate degradation via CoA ligation |
| ko00633 | 0.005260667±0.002449021 | 0.004008267±0.000369481 | 0.003861453±0.002428355 | 0.003869793±0.001327649 | 0.003208751±0.002145126 | 0.008481722±0.000381734 | Metabolism | Xenobiotics Biodegradation and Metabolism | Trinitrotoluene degradation |
| ko00640 | 1.243917637±0.094594368 | 1.082550695±0.047323214 | 1.064732352±0.078767931 | 1.047266953±0.05821773 | 0.886346078±0.06353226 | 0.933324732±0.267289909 | Metabolism | Carbohydrate Metabolism | Propanoate metabolism |
| ko00641 | 0.060794672±0.019467782 | 0.081226274±0.029724555 | 0.076124586±0.02056723 | 0.10228043±0.05871665 | 0.018177515±0.003539911 | 0.056673679±0.014154609 | Metabolism | Xenobiotics Biodegradation and Metabolism | 3-Chloroacrylic acid degradation |
| ko00642 | 2.06191415±0.259369527 | 2.713048398±0.343136074 | 2.773358826±0.281301062 | 3.129609574±0.434461353 | 2.970360698±0.295500162 | 2.406803338±0.235365925 | Metabolism | Xenobiotics Biodegradation and Metabolism | Ethylbenzene degradation |
| ko00643 | 0.015650857±0.005684486 | 0.014825591±0.001701873 | 0.009238041±0.002815929 | 0.013122522±0.00227334 | 0.009565253±0.002195867 | 0.014702054±0.002595063 | Metabolism | Xenobiotics Biodegradation and Metabolism | Styrene degradation |
| ko00650 | 3.318484372±0.241547301 | 3.824546795±0.175567371 | 4.104249184±0.255431099 | 3.918090592±0.093945546 | 3.276183082±0.124025193 | 3.559343312±0.299811189 | Metabolism | Carbohydrate Metabolism | Butanoate metabolism |
| ko00660 | 0.381564944±0.010953619 | 0.239370309±0.071292483 | 0.215003771±0.024809083 | 0.29220193±0.022874013 | 0.470086582±0.076139003 | 0.55871935±0.178516013 | Metabolism | Carbohydrate Metabolism | C5-Branched dibasic acid metabolism |
| ko00670 | 1.972870705±0.09786112 | 1.847768172±0.050061958 | 1.843992265±0.04049467 | 1.810064811±0.031624428 | 1.65625621±0.067166452 | 1.688535868±0.177545715 | Metabolism | Metabolism of Cofactors and Vitamins | One carbon pool by folate |
| ko00680 | 0.482839493±0.049731693 | 0.512635254±0.035024549 | 0.456104221±0.028463354 | 0.47626082±0.088962727 | 0.338521741±0.015316209 | 0.422671388±0.093381049 | Metabolism | Energy Metabolism | Methane metabolism |
| ko00710 | 2.495661399±0.092046338 | 2.779060616±0.078194451 | 2.644899126±0.160433225 | 2.651419602±0.169969246 | 1.945681398±0.193077879 | 2.030829818±0.214318783 | Metabolism | Energy Metabolism | Carbon fixation in photosynthetic organisms |
| ko00720 | 1.388737768±0.105611618 | 1.243000519±0.111534507 | 1.12850727±0.095718856 | 1.147795578±0.10127544 | 1.14509291±0.095171619 | 1.056320231±0.33527208 | Metabolism | Energy Metabolism | Reductive carboxylate cycle (CO2 fixation) |
| ko00730 | 1.240459979±0.124063948 | 1.194289754±0.120519253 | 1.116036841±0.093905584 | 1.119246316±0.222529878 | 0.871827092±0.068409837 | 0.86721252±0.081967402 | Metabolism | Metabolism of Cofactors and Vitamins | Thiamine metabolism |
| ko00740 | 0.589769961±0.012688757 | 0.482707856±0.009420456 | 0.460638703±0.055940848 | 0.474728841±0.1106066 | 0.54122954±0.019425666 | 0.504376008±0.04180181 | Metabolism | Metabolism of Cofactors and Vitamins | Riboflavin metabolism |
| ko00750 | 0.565669405±0.014202892 | 0.502341755±0.012513174 | 0.556056023±0.020984558 | 0.531326645±0.032308736 | 0.44951289±0.009547736 | 0.484921587±0.050918252 | Metabolism | Metabolism of Cofactors and Vitamins | Vitamin B6 metabolism |
| ko00760 | 1.25094021±0.000399883 | 1.139751762±0.040047006 | 1.221815664±0.075858904 | 1.169097356±0.089054218 | 1.163236432±0.027997939 | 0.973259389±0.117161625 | Metabolism | Metabolism of Cofactors and Vitamins | Nicotinate and nicotinamide metabolism |
| ko00770 | 1.178392143±0.016157722 | 0.961979716±0.111907102 | 0.848723358±0.040112745 | 0.937625548±0.187397661 | 1.071327178±0.069497739 | 1.077431409±0.094432764 | Metabolism | Metabolism of Cofactors and Vitamins | Pantothenate and CoA biosynthesis |
| ko00780 | 0.885748469±0.010916144 | 0.794788756±0.039469812 | 0.920257269±0.093193016 | 0.810319105±0.160524106 | 0.5301989±0.046622057 | 0.478095216±0.039574239 | Metabolism | Metabolism of Cofactors and Vitamins | Biotin metabolism |
| ko00785 | 0.066629889±0.013307735 | 0.075871476±0.019996836 | 0.087334342±0.019230921 | 0.077320925±0.014559209 | 0.059555549±0.003224114 | 0.04068576±0.017875353 | Metabolism | Metabolism of Cofactors and Vitamins | Lipoic acid metabolism |
| ko00790 | 0.785108368±0.02067662 | 0.651988824±0.077677028 | 0.798486611±0.033410988 | 0.734512309±0.077650349 | 0.571125756±0.028871041 | 0.556552621±0.035409717 | Metabolism | Metabolism of Cofactors and Vitamins | Folate biosynthesis |
| ko00791 | 0.046077163±0.005053866 | 0.040693354±0.008796828 | 0.067245922±0.002902454 | 0.048816801±0.01321305 | 0.032012707±0.011726977 | 0.026961219±0.009974666 | Metabolism | Xenobiotics Biodegradation and Metabolism | Atrazine degradation |
| ko00830 | 0.016904929±0.005822166 | 0.028251272±0.009711735 | 0.046576507±0.009294845 | 0.042161886±0.018160152 | 0.027914561±0.00372474 | 0.023412897±0.012528672 | Metabolism | Metabolism of Cofactors and Vitamins | Retinol metabolism |
| ko00860 | 2.119677468±0.281692294 | 1.836934365±0.355386403 | 1.455908074±0.142531206 | 1.32146738±0.177693425 | 1.225699588±0.067359628 | 1.459696341±0.175130881 | Metabolism | Metabolism of Cofactors and Vitamins | Porphyrin and chlorophyll metabolism |
| ko00900 | 1.6407607±0.088380541 | 1.54567546±0.067840288 | 1.491966883±0.122903433 | 1.45368005±0.056397562 | 1.47626372±0.062491394 | 1.38870951±0.09415111 | Metabolism | Metabolism of Terpenoids and Polyketides | Terpenoid backbone biosynthesis |
| ko00901 | 0±0 | 0±0 | 0±0 | 0±0 | 0±0 | 0±0 | Metabolism | Biosynthesis of Other Secondary Metabolites | Indole alkaloid biosynthesis |
| ko00902 | 0±0 | 0±0 | 0±0 | 0±0 | 0±0 | 0±0 | Metabolism | Metabolism of Terpenoids and Polyketides | Monoterpenoid biosynthesis |
| ko00903 | 2.59598079±0.336035978 | 3.188589075±0.341674141 | 3.126898876±0.255100027 | 3.411102873±0.333304575 | 3.315663425±0.305955746 | 2.773242682±0.190178899 | Metabolism | Metabolism of Terpenoids and Polyketides | Limonene and pinene degradation |
| ko00906 | 0.00194907±0.000719255 | 0.001281792±0.000772687 | 0.000753133±0.000477655 | 0.00078765±0.000482936 | 2.14E-04±0.000245659 | 3.96E-04±0.000437251 | Metabolism | Metabolism of Terpenoids and Polyketides | Carotenoid biosynthesis |
| ko00908 | 0.410547436±0.001742862 | 0.366500517±0.014064016 | 0.341590983±0.039993161 | 0.307026741±0.024372607 | 0.342154836±0.013619298 | 0.242210954±0.090780639 | Metabolism | Metabolism of Terpenoids and Polyketides | Zeatin biosynthesis |
| ko00910 | 2.18201038±0.027925348 | 2.057723487±0.093453641 | 3.003470919±0.184076079 | 2.678898327±0.048043859 | 3.221950266±0.188780328 | 3.033938529±0.670387139 | Metabolism | Energy Metabolism | Nitrogen metabolism |
| ko00920 | 0.455497212±0.009117827 | 0.406618021±0.110348709 | 0.297981994±0.066025352 | 0.394084896±0.033446375 | 0.427725569±0.04155509 | 0.449564387±0.099478552 | Metabolism | Energy Metabolism | Sulfur metabolism |
| ko00930 | 0.005278027±0.003538416 | 0.002512672±0.002379724 | 0.004283977±0.000380353 | 0.004005251±0.001330533 | 0.001917776±0.000813576 | 0.003526793±0.00131227 | Metabolism | Xenobiotics Biodegradation and Metabolism | Caprolactam degradation |
| ko00940 | 1.577319386±0.331058207 | 1.271140747±0.186993787 | 2.632171998±0.399325592 | 2.291133772±0.677337367 | 4.32226092±0.464638297 | 2.807716864±1.412463188 | Metabolism | Biosynthesis of Other Secondary Metabolites | Phenylpropanoid biosynthesis |
| ko00941 | 0.006682457±0.002521935 | 0.012803263±0.006880624 | 0.016312515±0.004003647 | 0.014785322±0.006044951 | 0.000876168±0.000261104 | 0.004859586±0.003846482 | Metabolism | Biosynthesis of Other Secondary Metabolites | Flavonoid biosynthesis |
| ko00943 | 0±0 | 0±0 | 9.71447E-06±1.24685E-05 | 2.16667E-05±1.76917E-05 | 0.000113916±0.00015287 | 1.84188E-06±2.60481E-06 | Metabolism | Biosynthesis of Other Secondary Metabolites | Isoflavonoid biosynthesis |
| ko00944 | 0.009380811±0.001684368 | 0.009383285±0.002219698 | 0.034594294±0.014358574 | 0.021731588±0.004443138 | 0.059784428±0.011693767 | 0.045185676±0.016105628 | Metabolism | Biosynthesis of Other Secondary Metabolites | Flavone and flavonol biosynthesis |
| ko00945 | 0.000325578±0.000275964 | 4.02E-06±3.48634E-06 | 2.97E-05±3.5235E-05 | 3.52E-04±0.00040881 | 0.000114205±0.00012195 | 0.000138535±9.81656E-05 | Metabolism | Biosynthesis of Other Secondary Metabolites | Stilbenoid, diarylheptanoid and gingerol biosynthesis |
| ko00950 | 0.160816904±0.011688089 | 0.200996013±0.070857443 | 0.217509425±0.051528958 | 0.202968989±0.009357797 | 0.2720582±0.01942046 | 0.193538598±0.039712139 | Metabolism | Biosynthesis of Other Secondary Metabolites | Isoquinoline alkaloid biosynthesis |
| ko00960 | 0.425579289±0.014945449 | 0.390618067±0.022992486 | 0.400093503±0.021436375 | 0.403245432±0.03429468 | 0.519161256±0.068412778 | 0.443136078±0.041410033 | Metabolism | Biosynthesis of Other Secondary Metabolites | Tropane, piperidine and pyridine alkaloid biosynthesis |
| ko00965 | 0±0 | 0±0 | 0±0 | 0±0 | 0±0 | 0±0 | Metabolism | Biosynthesis of Other Secondary Metabolites | Betalain biosynthesis |
| ko00970 | 7.030028351±0.737005495 | 6.884063167±0.339598502 | 7.828759474±0.457170292 | 7.826835681±1.107137598 | 5.767407724±0.299041566 | 6.223070299±1.174407924 | Genetic Information Processing | Translation | Aminoacyl-tRNA biosynthesis |
| ko00980 | 0.019191024±0.006498328 | 0.026311839±0.004303806 | 0.025015323±0.006715605 | 0.032000564±0.005401272 | 0.023536535±0.007728722 | 0.018536105±0.006867818 | Metabolism | Xenobiotics Biodegradation and Metabolism | Metabolism of xenobiotics by cytochrome P450 |
| ko00982 | 0.018013063±0.006705321 | 0.024438627±0.004326906 | 0.019751806±0.002764839 | 0.023902597±0.009243263 | 0.00839767±0.002361383 | 0.009351504±0.004880915 | Metabolism | Xenobiotics Biodegradation and Metabolism | Drug metabolism - cytochrome P450 |
| ko00983 | 1.713916596±0.02390603 | 1.612995455±0.148544578 | 2.432149287±0.31462991 | 2.146930687±0.25354798 | 2.317320252±0.069224403 | 2.341627122±0.344796137 | Metabolism | Xenobiotics Biodegradation and Metabolism | Drug metabolism - other enzymes |
| ko01001 | 2.131347955±0.350589761 | 2.342040502±0.094450389 | 2.517511746±0.323618934 | 3.099572048±0.626031593 | 2.829825345±0.266558762 | 2.846724093±0.172586824 | Metabolism | Enzyme Families | Protein kinases |
| ko01002 | 11.016576±0.152678327 | 10.58168072±1.401396745 | 13.1873144±0.858275253 | 11.76138361±0.606789418 | 13.06216352±0.456598053 | 10.709924±2.047535883 | Metabolism | Enzyme Families | Peptidases |
| ko01003 | 1.591721707±0.001778554 | 1.618291839±0.107896346 | 1.629442185±0.097038224 | 1.704209149±0.134297297 | 1.899328123±0.11043909 | 1.859520737±0.217626625 | Metabolism | Glycan Biosynthesis and Metabolism | Glycosyltransferases |
| ko01004 | 3.895776446±0.089756547 | 3.493051974±0.390613268 | 5.658892207±0.5730773 | 4.727905011±1.06178174 | 4.100715629±0.269043197 | 3.845657643±0.536526665 | Metabolism | Lipid Metabolism | Lipid biosynthesis proteins |
| ko01005 | 1.373147066±0.026268361 | 1.206834747±0.12513762 | 1.443808008±0.180102388 | 1.428175896±0.370990576 | 1.561054746±0.13111248 | 1.242681656±0.08865157 | Metabolism | Glycan Biosynthesis and Metabolism | Lipopolysaccharide biosynthesis proteins |
| ko01020 | 0±0 | 0±0 | 3.66E-06±5.09988E-06 | 0.00E+00±0 | 2.40E-06±2.78832E-06 | 2.00454E-08±2.83484E-08 | Environmental Information Processing | Signaling Molecules and Interaction | Enzyme-linked receptors |
| ko01040 | 0.717710735±0.16520508 | 0.618585513±0.01135103 | 0.88963012±0.00844027 | 0.758765871±0.202320914 | 0.60663575±0.091816641 | 0.741950362±0.077477309 | Metabolism | Lipid Metabolism | Biosynthesis of unsaturated fatty acids |
| ko01051 | 0.745152374±0.053608501 | 1.027559798±0.03110522 | 1.005106896±0.125086914 | 0.92442679±0.101998443 | 0.630469518±0.118643688 | 0.712721603±0.124073772 | Metabolism | Metabolism of Terpenoids and Polyketides | Biosynthesis of ansamycins |
| ko01053 | 0.024906761±0.003414216 | 0.024585061±0.008418668 | 0.076581514±0.009369157 | 0.051972882±0.031057128 | 0.054824884±0.014428097 | 0.071863275±0.030593491 | Metabolism | Metabolism of Terpenoids and Polyketides | Biosynthesis of siderophore group nonribosomal peptides |
| ko01055 | 0.543747731±0.018767927 | 0.368680061±0.043545931 | 0.454421376±0.134500568 | 0.600746173±0.147538418 | 0.476426936±0.071793429 | 0.49880981±0.0043359 | Metabolism | Metabolism of Terpenoids and Polyketides | Biosynthesis of vancomycin group antibiotics |
| ko01056 | 0±0 | 0±0 | 0±0 | 0±0 | 0±0 | 0±0 | Metabolism | Metabolism of Terpenoids and Polyketides | Biosynthesis of type II polyketide backbone |
| ko02000 | 26.47706111±2.396174639 | 24.42308176±2.933158211 | 21.32596507±1.44912927 | 23.46186007±5.313666004 | 14.43314799±0.25386144 | 19.8045005±2.897661122 | Environmental Information Processing | Membrane Transport | Transporters |
| ko02010 | 6.008152516±2.110533445 | 5.594895245±0.381345021 | 4.623799102±0.490060228 | 5.461188626±1.609259848 | 3.497424089±0.444068816 | 5.167329921±0.586505183 | Environmental Information Processing | Membrane Transport | ABC transporters |
| ko02020 | 4.27631212±0.233447226 | 3.714848502±0.111056063 | 3.813991555±0.302722845 | 4.122887537±0.523588227 | 4.886674487±0.964626147 | 6.117959857±0.974267294 | Environmental Information Processing | Signal Transduction | Two-component system |
| ko02022 | 2.47166379±0.059307007 | 2.235792301±0.151543381 | 1.911168372±0.207932186 | 2.197142679±0.484670229 | 2.554551467±0.482325167 | 2.813533901±0.298474046 | Environmental Information Processing | Signal Transduction | Two-component system |
| ko02030 | 0.624534842±0.183113181 | 0.172957956±0.02716799 | 0.236291192±0.081009584 | 0.495311481±0.303773955 | 1.09187358±0.601824494 | 2.240365958±1.124889211 | Cellular Processes | Cell Motility | Bacterial chemotaxis |
| ko02035 | 0.701866884±0.210512839 | 0.228137691±0.053333347 | 0.370366412±0.199869302 | 0.702811932±0.216953916 | 1.099824902±0.643977713 | 2.606987733±1.451570388 | Cellular Processes | Cell Motility | Bacterial motility proteins |
| ko02040 | 0.080619778±0.030554984 | 0.055983243±0.017309192 | 0.053846319±0.020569429 | 0.060365892±0.029864664 | 0.106474311±0.055663591 | 0.323637563±0.231191631 | Cellular Processes | Cell Motility | Flagellar assembly |
| ko02042 | 0.783225694±0.047516817 | 0.666907998±0.059374819 | 0.63529048±0.085071431 | 0.67978971±0.055514337 | 0.589829877±0.031518259 | 0.55497798±0.125390037 | Environmental Information Processing | Signaling Molecules and Interaction | Bacterial toxins |
| ko02044 | 3.395523591±0.190152543 | 3.480961963±0.085797569 | 3.739736879±0.185109302 | 4.108734731±0.443186291 | 2.8632058±0.031850654 | 3.268930623±0.344411277 | Environmental Information Processing | Membrane Transport | Secretion system |
| ko02060 | 0.084514803±0.021428959 | 0.058005642±0.01719552 | 0.05043929±0.023854544 | 0.064501838±0.039055597 | 0.048415911±0.022110696 | 0.121701545±0.071424326 | Environmental Information Processing | Membrane Transport | Phosphotransferase system (PTS) |
| ko03000 | 2.755680474±0.078422324 | 3.100865798±0.581338236 | 1.960843772±0.286453631 | 2.303948609±0.839667022 | 2.021790086±0.27873265 | 2.323886681±0.01255659 | Genetic Information Processing | Transcription | Transcription factors |
| ko03010 | 2.240857535±0.084769136 | 2.310248764±0.081757308 | 1.644107151±0.137241054 | 1.795420198±0.093558796 | 1.280171665±0.132563004 | 1.587262885±0.293085956 | Genetic Information Processing | Translation | Ribosome |
| ko03011 | 2.240857535±0.084769136 | 2.310248764±0.081757308 | 1.644107151±0.137241054 | 1.795420198±0.093558796 | 1.280171665±0.132563004 | 1.587262885±0.293085956 | Genetic Information Processing | Translation | Ribosome |
| ko03012 | 3.644088861±0.102133963 | 3.712103132±0.269011636 | 3.705671098±0.232527126 | 3.981514145±0.239275702 | 2.969317842±0.093479124 | 3.314033464±0.149997814 | Genetic Information Processing | Translation | Translation factors |
| ko03018 | 3.591273476±0.071314889 | 2.863284184±0.227740725 | 3.161368748±0.321368992 | 3.200302373±0.519951795 | 3.254297274±0.192489056 | 3.167166585±0.189784005 | Genetic Information Processing | Folding, Sorting and Degradation | RNA degradation |
| ko03020 | 12.37124887±2.920558759 | 12.07605811±0.180582842 | 10.42570439±1.40055583 | 11.8560578±2.786981715 | 10.62660147±1.124697079 | 10.03045577±2.116465763 | Genetic Information Processing | Transcription | RNA polymerase |
| ko03022 | 2.52E-05±3.03122E-06 | 8.57E-06±1.30373E-06 | 6.22E-06±4.49155E-06 | 1.04E-05±6.60677E-06 | 4.68143E-06±6.62054E-06 | 1.50711E-05±1.66265E-05 | Genetic Information Processing | Transcription | Basal transcription factors |
| ko03030 | 4.709872743±0.436637589 | 4.624150828±0.136535565 | 4.803337431±0.200896958 | 4.6201474±0.36423008 | 4.032756905±0.252662653 | 4.775821383±0.768569313 | Genetic Information Processing | Replication and Repair | DNA replication |
| ko03032 | 8.597396949±0.415067827 | 9.026972347±0.171475926 | 8.474686058±0.283275849 | 8.494130391±0.805878877 | 7.382676183±0.458189087 | 8.415433081±0.712840714 | Genetic Information Processing | Replication and Repair | DNA replication proteins |
| ko03036 | 11.6887476±0.22155228 | 11.41721092±0.686414411 | 9.392454861±0.269955781 | 9.269758504±0.330460997 | 9.11540184±0.333959151 | 9.645799185±2.125953308 | Genetic Information Processing | Replication and Repair | Chromosome |
| ko03040 | 0.000715206±0.000217931 | 0.002354558±0.00087988 | 0.002380114±0.000804187 | 0.00259184±0.00147072 | 0.001476801±0.000347314 | 0.001960528±0.001111552 | Genetic Information Processing | Transcription | Spliceosome |
| ko03041 | 0.002202911±0.000304604 | 0.003844424±0.001100837 | 0.003559715±0.001124892 | 0.004123194±0.000994281 | 0.002365511±0.000401891 | 0.00307472±0.001058024 | Genetic Information Processing | Transcription | Spliceosome |
| ko03050 | 4.13E-05±3.93794E-05 | 7.89E-05±4.86688E-05 | 6.52409E-05±3.72162E-05 | 3.79974E-05±3.16795E-05 | 1.45E-04±0.000141194 | 2.90E-05±3.57857E-05 | Genetic Information Processing | Folding, Sorting and Degradation | Proteasome |
| ko03051 | 0.349651814±0.041178201 | 0.317811771±0.030675138 | 0.34346343±0.051921972 | 0.331865307±0.056984516 | 0.279188001±0.022190844 | 0.282568687±0.051657515 | Genetic Information Processing | Folding, Sorting and Degradation | Proteasome |
| ko03060 | 2.583570207±0.079333749 | 2.522866374±0.139530854 | 2.764408696±0.071249373 | 3.170822379±0.208638517 | 2.318107254±0.116908734 | 2.470424147±0.478239298 | Genetic Information Processing | Folding, Sorting and Degradation | Protein export |
| ko03070 | 2.713007909±0.158920626 | 2.686598421±0.119181671 | 2.983688652±0.080776504 | 3.242463271±0.220543356 | 2.359131584±0.047165048 | 2.545729937±0.514525581 | Environmental Information Processing | Membrane Transport | Bacterial secretion system |
| ko03110 | 5.199033918±0.036833143 | 4.900539709±0.567778778 | 5.336557556±0.4981568 | 5.282550716±0.463925772 | 4.467436226±0.35983543 | 4.528506543±0.519830271 | Genetic Information Processing | Folding, Sorting and Degradation | Chaperones and folding catalysts |
| ko03310 | 0±0 | 6.37E-06±4.38412E-06 | 5.96E-06±3.29311E-06 | 3.75E-06±3.00834E-06 | 0±0 | 6.53E-06±9.2335E-06 | Environmental Information Processing | Signaling Molecules and Interaction | Nuclear receptors |
| ko03320 | 2.01711501±0.172043579 | 1.929198865±0.342142364 | 3.018830697±0.424052964 | 2.366694613±0.200153459 | 2.242561244±0.098516046 | 2.009300459±0.52954918 | Organismal Systems | Endocrine System | PPAR signaling pathway |
| ko03400 | 19.15036568±1.046799719 | 19.40906642±1.508157577 | 22.57188634±1.023483565 | 21.99140724±1.591472582 | 19.34637991±0.732837844 | 20.26675016±0.763727025 | Genetic Information Processing | Replication and Repair | DNA repair and recombination proteins |
| ko03410 | 1.794754383±0.259041823 | 1.690192184±0.048684318 | 1.732296544±0.114809564 | 1.576770436±0.073548213 | 1.380354537±0.060381556 | 1.561250467±0.116505745 | Genetic Information Processing | Replication and Repair | Base excision repair |
| ko03420 | 4.503646991±0.412481351 | 4.521113963±0.475172163 | 5.988235877±0.190765568 | 5.313107254±0.520234389 | 4.685499164±0.365624741 | 5.25192592±0.347239966 | Genetic Information Processing | Replication and Repair | Nucleotide excision repair |
| ko03430 | 5.750506577±0.286913518 | 5.920012897±0.111326873 | 6.226484681±0.448954411 | 5.827993518±0.341976369 | 5.294441281±0.334427441 | 5.773859216±0.452895179 | Genetic Information Processing | Replication and Repair | Mismatch repair |
| ko03440 | 6.527016918±0.481636075 | 7.076058956±0.582065972 | 7.927510588±0.665896782 | 7.933293615±0.836713267 | 7.004503564±0.25487597 | 6.902495456±0.172071825 | Genetic Information Processing | Replication and Repair | Homologous recombination |
| ko03450 | 0.002108974±0.000349906 | 0.003253322±0.001340499 | 0.000518608±0.000226406 | 0.000653278±0.000153549 | 2.12E-04±0.000272553 | 0.000355877±0.000354984 | Genetic Information Processing | Replication and Repair | Non-homologous end-joining |
| ko04010 | 0±0 | 5.03585E-05±7.06735E-05 | 4.14E-05±2.35334E-05 | 1.17E-04±9.19933E-05 | 0.000276305±0.000210193 | 0.000421969±0.000365738 | Environmental Information Processing | Signal Transduction | MAPK signaling pathway |
| ko04011 | 0.033566689±5.53293E-05 | 0.032997779±0.007221353 | 0.020613884±0.005897199 | 0.022894013±0.007109982 | 0.012556939±0.000574001 | 0.015030697±0.002558271 | Environmental Information Processing | Signal Transduction | MAPK signaling pathway - yeast |
| ko04012 | 0±0 | 0±0 | 1.78645E-05±2.19665E-05 | 0.000032884±2.7523E-05 | 1.38186E-05±1.16978E-05 | 1.02E-05±1.32209E-05 | Environmental Information Processing | Signal Transduction | ErbB signaling pathway |
| ko04013 | 0±0 | 0±0 | 0±0 | 0±0 | 0±0 | 0±0 | Environmental Information Processing | Signal Transduction | MAPK signaling pathway - fly |
| ko04020 | 6.76E-05±2.22949E-05 | 5.71E-05±5.39694E-05 | 0.000371627±0.000263957 | 0.000509217±0.000417129 | 0.000438414±0.000288304 | 0.000249583±0.000217164 | Environmental Information Processing | Signal Transduction | Calcium signaling pathway |
| ko04030 | 0±0 | 0.000129425±9.67419E-05 | 3.04E-05±2.25427E-05 | 2.20E-04±0.000165016 | 4.42E-05±3.14324E-05 | 0.000320618±0.000108277 | Environmental Information Processing | Signaling Molecules and Interaction | G protein-coupled receptors |
| ko04031 | 0±0 | 0±0 | 0±0 | 2.6844E-06±3.79632E-06 | 1.03E-05±8.83781E-06 | 5.39848E-06±4.85069E-06 | Environmental Information Processing | Signaling Molecules and Interaction | GTP-binding proteins |
| ko04040 | 0.057136743±0.016544278 | 0.056948246±0.007576789 | 0.075023525±0.009649296 | 0.057595037±0.023710974 | 0.068260766±0.017161455 | 0.048360155±0.016597406 | Environmental Information Processing | Signaling Molecules and Interaction | Ion channels |
| ko04050 | 0±0 | 0±0 | 3.66E-06±5.09988E-06 | 0.00E+00±0 | 2.40E-06±2.78832E-06 | 2.00454E-08±2.83484E-08 | Environmental Information Processing | Signaling Molecules and Interaction | Cytokine receptors |
| ko04052 | 0±0 | 0±0 | 0±0 | 0±0 | 0±0 | 0±0 | Environmental Information Processing | Signaling Molecules and Interaction | Cytokines |
| ko04060 | 0±0 | 0±0 | 0±0 | 0±0 | 0±0 | 0±0 | Environmental Information Processing | Signaling Molecules and Interaction | Cytokine-cytokine receptor interaction |
| ko04062 | 1.71249E-05±1.71249E-05 | 2.63822E-07±3.731E-07 | 5.00846E-05±6.74739E-05 | 7.38761E-05±5.65784E-05 | 0.000166824±8.45828E-05 | 1.77E-04±0.000155704 | Organismal Systems | Immune System | Chemokine signaling pathway |
| ko04070 | 0.241456163±0.012079368 | 0.198801885±0.00978907 | 0.16693557±0.008555062 | 0.17360802±0.01530365 | 0.175466003±0.020189415 | 0.131550414±0.033084917 | Environmental Information Processing | Signal Transduction | Phosphatidylinositol signaling system |
| ko04080 | 0.001382479±0.000906335 | 0.003329137±0.003278229 | 0.002261212±0.001222169 | 0.003151493±0.001524069 | 0.003252691±0.001870766 | 0.002145915±0.002134872 | Environmental Information Processing | Signaling Molecules and Interaction | Neuroactive ligand-receptor interaction |
| ko04090 | 0.862708994±0.13655084 | 0.70514179±0.110507109 | 1.104328067±0.050331755 | 0.824637584±0.113855773 | 1.052651333±0.104302568 | 0.635806941±0.29928626 | Environmental Information Processing | Signaling Molecules and Interaction | Cellular antigens |
| ko04091 | 1.70E-05±1.04704E-05 | 0.000210085±0.000116933 | 0.000150824±0.000113257 | 0.00023618±0.00020753 | 7.57E-06±6.46601E-06 | 8.52E-05±7.14437E-05 | Environmental Information Processing | Signaling Molecules and Interaction | Glycan bindng proteins |
| ko04110 | 0.047386375±0.005970123 | 0.050838285±0.008949169 | 0.044131147±0.008911159 | 0.054922628±0.022555967 | 0.047143155±0.007019061 | 0.0272506±0.009043398 | Cellular Processes | Cell Growth and Death | Cell cycle |
| ko04111 | 0.047687275±0.005800672 | 0.051030251±0.008838081 | 0.044624093±0.009075203 | 0.055463548±0.022777727 | 0.047412494±0.006914046 | 0.02780449±0.009440034 | Cellular Processes | Cell Growth and Death | Cell cycle - yeast |
| ko04112 | 3.462126133±0.274274173 | 3.004023203±0.158975433 | 3.166930217±0.108431221 | 3.098706045±0.088878824 | 3.03142297±0.261495805 | 3.846830402±0.990871899 | Cellular Processes | Cell Growth and Death | Cell cycle - Caulobacter |
| ko04113 | 0.13653637±0.025914493 | 0.090000345±0.039671536 | 0.051396046±0.018982734 | 0.057131147±0.014403305 | 0.079394605±0.013272531 | 0.102316654±0.050665067 | Cellular Processes | Cell Growth and Death | Meiosis - yeast |
| ko04114 | 0.003966964±0.000831036 | 0.002044073±0.001524804 | 0.001450798±6.13967E-05 | 0.000863329±0.000557821 | 0.000510431±0.000421793 | 3.63E-04±0.000214284 | Cellular Processes | Cell Growth and Death | Oocyte meiosis |
| ko04115 | 1.86E-06±1.8586E-06 | 1.37E-05±1.15933E-05 | 6.79E-06±4.34761E-06 | 5.03E-05±1.34982E-05 | 8.80E-05±9.25955E-05 | 5.62E-05±4.26076E-05 | Cellular Processes | Cell Growth and Death | p53 signaling pathway |
| ko04120 | 0.047204095±0.005933183 | 0.050363404±0.009087898 | 0.044213899±0.009093001 | 0.055663466±0.021910465 | 0.048400774±0.00755491 | 0.028426798±0.009237138 | Genetic Information Processing | Folding, Sorting and Degradation | Ubiquitin mediated proteolysis |
| ko04121 | 0.049872359±0.006027301 | 0.054124661±0.01050676 | 0.044970545±0.008740507 | 0.056831364±0.021250234 | 0.050243529±0.007783842 | 0.030822379±0.008657217 | Genetic Information Processing | Folding, Sorting and Degradation | Ubiquitin system |
| ko04131 | 0±0 | 0±0 | 1.08E-04±0.000149456 | 7.53E-06±8.4028E-06 | 0±0 | 1.42455E-05±1.10359E-05 | Genetic Information Processing | Folding, Sorting and Degradation | SNAREs |
| ko04140 | 0±0 | 0±0 | 1.04284E-06±1.4748E-06 | 6.43116E-06±9.09503E-06 | 2.69405E-05±3.80996E-05 | 1.22338E-05±1.73012E-05 | Cellular Processes | Transport and Catabolism | Regulation of autophagy |
| ko04141 | 0.381561717±0.040045256 | 0.370002012±0.025803485 | 0.422282411±0.066508237 | 0.399177428±0.060994736 | 0.335746505±0.020244632 | 0.330806463±0.060060695 | Genetic Information Processing | Folding, Sorting and Degradation | Protein processing in endoplasmic reticulum |
| ko04142 | 1.338073517±0.163354179 | 1.298392297±0.355811256 | 1.518967519±0.305952373 | 1.450763212±0.084849618 | 0.923319861±0.17605062 | 0.765950318±0.301978933 | Cellular Processes | Transport and Catabolism | Lysosome |
| ko04144 | 0.026303644±0.011536604 | 0.022054419±0.00363042 | 0.01490022±0.00318602 | 0.010542892±0.004518847 | 0.030107495±0.001011668 | 0.022811082±0.012843438 | Cellular Processes | Transport and Catabolism | Endocytosis |
| ko04145 | 1.48E-05±1.31247E-05 | 0.000413818±0.000217256 | 0.000584543±0.000421716 | 0.000342679±0.000224895 | 0.000264166±0.0001774 | 0.000517902±0.000546053 | Cellular Processes | Transport and Catabolism | Phagosome |
| ko04146 | 2.098902924±0.199895116 | 1.974421246±0.344326155 | 3.032931456±0.441729735 | 2.402105771±0.216559005 | 2.315610059±0.086070399 | 2.035200718±0.530326233 | Cellular Processes | Transport and Catabolism | Peroxisome |
| ko04150 | 0±0 | 2.56876E-07±3.63278E-07 | 8.40E-06±7.43521E-06 | 1.19E-05±9.84728E-06 | 4.90501E-05±4.97547E-05 | 2.01E-05±1.87507E-05 | Environmental Information Processing | Signal Transduction | mTOR signaling pathway |
| ko04210 | 0.076070578±0.005370317 | 0.090170543±0.015767456 | 0.120224724±0.003840129 | 0.093825405±0.026548852 | 0.121856302±0.023954652 | 0.070052712±0.043671115 | Cellular Processes | Cell Growth and Death | Apoptosis |
| ko04260 | 0.003421064±0.000963623 | 0.003097228±0.001524926 | 0.00080383±0.00015965 | 0.000730466±0.000225208 | 2.75E-04±0.000176979 | 0.000412893±0.000303544 | Organismal Systems | Circulatory System | Cardiac muscle contraction |
| ko04270 | 0.000392748±0.000237121 | 0.000553625±0.000463955 | 0.001127608±0.000389285 | 0.00168747±0.001471893 | 0.002769315±0.002858599 | 0.001436209±0.001101028 | Organismal Systems | Circulatory System | Vascular smooth muscle contraction |
| ko04310 | 1.49843E-05±1.49843E-05 | 4.89809E-07±3.48055E-07 | 5.02133E-05±5.92313E-05 | 7.06563E-05±5.24867E-05 | 0.000148423±7.55637E-05 | 1.78E-04±0.000119928 | Environmental Information Processing | Signal Transduction | Wnt signaling pathway |
| ko04320 | 0±0 | 2.44698E-05±2.25706E-05 | 7.75E-06±9.78153E-06 | 4.09E-05±1.71193E-05 | 1.87951E-05±1.38938E-05 | 5.56E-06±6.00804E-06 | Organismal Systems | Development | Dorso-ventral axis formation |
| ko04330 | 1.55E-05±8.65458E-06 | 1.22559E-05±8.74547E-06 | 6.78209E-05±9.52163E-05 | 5.18152E-06±3.86434E-06 | 2.33109E-07±3.29666E-07 | 0±0 | Environmental Information Processing | Signal Transduction | Notch signaling pathway |
| ko04340 | 8.00548E-05±8.00548E-05 | 3.29E-06±4.65854E-06 | 0.000019658±2.1025E-05 | 4.09617E-05±3.68632E-05 | 4.88533E-06±5.63004E-06 | 9.63E-06±1.36155E-05 | Environmental Information Processing | Signal Transduction | Hedgehog signaling pathway |
| ko04350 | 1.71249E-05±1.71249E-05 | 2.63822E-07±3.731E-07 | 7.21E-05±4.77119E-05 | 4.36E-05±5.81154E-05 | 0.000175828±8.05958E-05 | 1.70E-04±0.000169105 | Environmental Information Processing | Signal Transduction | TGF-beta signaling pathway |
| ko04360 | 5.58E-05±2.57817E-05 | 1.63E-05±1.33235E-05 | 5.07E-05±5.89312E-05 | 1.06E-04±3.83081E-05 | 0.000152987±6.49787E-05 | 1.63E-04±0.000132408 | Organismal Systems | Development | Axon guidance |
| ko04370 | 0±0 | 0±0 | 0±0 | 0±0 | 0±0 | 0±0 | Environmental Information Processing | Signal Transduction | VEGF signaling pathway |
| ko04510 | 0.011549413±0.003759728 | 0.013304199±0.010710734 | 0.006570227±0.001494909 | 0.007101372±0.003308737 | 0.010521257±0.008247383 | 0.010058156±0.00506453 | Cellular Processes | Cell Communication | Focal adhesion |
| ko04512 | 0.011532288±0.003776853 | 0.013305325±0.010709517 | 0.006729584±0.001676466 | 0.00719549±0.003496243 | 0.010583917±0.008222872 | 0.009980093±0.005057548 | Environmental Information Processing | Signaling Molecules and Interaction | ECM-receptor interaction |
| ko04514 | 0±0 | 0±0 | 0±0 | 7.30148E-06±1.03259E-05 | 0±0 | 3.7835E-06±5.35068E-06 | Environmental Information Processing | Signaling Molecules and Interaction | Cell adhesion molecules (CAMs) |
| ko04515 | 3.29E-06±3.28828E-06 | 1.43335E-07±2.02707E-07 | 1.76E-05±1.11445E-05 | 3.13E-05±2.72069E-05 | 3.53E-06±4.79247E-06 | 6.71304E-06±9.49368E-06 | Environmental Information Processing | Signaling Molecules and Interaction | Cell adhesion molecules (CAMs) |
| ko04516 | 0.011532288±0.003776853 | 0.01344087±0.010896861 | 0.006556047±0.001574415 | 0.007117094±0.003254793 | 0.010420852±0.008247724 | 0.009863803±0.005142511 | Environmental Information Processing | Signaling Molecules and Interaction | CAM ligands |
| ko04520 | 0.000122223±9.83477E-06 | 0.00031334±4.16693E-05 | 1.80E-04±0.000201078 | 1.44E-04±0.000138132 | 0.000143657±8.72017E-05 | 0.00019106±8.78223E-05 | Cellular Processes | Cell Communication | Adherens junction |
| ko04530 | 0.066896129±0.006723697 | 0.061479193±0.027871292 | 0.06859817±0.012353411 | 0.060176179±0.033451037 | 0.085208565±0.030897757 | 0.043869692±0.029344125 | Cellular Processes | Cell Communication | Tight junction |
| ko04540 | 0±0 | 2.72E-05±2.90992E-05 | 0±0 | 0.000108439±0.000122013 | 3.59E-04±0.000363864 | 0.000541433±0.000345692 | Cellular Processes | Cell Communication | Gap junction |
| ko04610 | 2.09E-06±1.62997E-06 | 1.29E-04±5.12826E-05 | 0.000218682±1.60483E-05 | 0.000299788±0.000195144 | 0.000155556±4.41541E-05 | 6.60E-05±4.08182E-05 | Organismal Systems | Immune System | Complement and coagulation cascades |
| ko04612 | 0.349569141±0.041114194 | 0.317928396±0.030353103 | 0.344951289±0.051875476 | 0.33391631±0.055769696 | 0.283881699±0.023674264 | 0.284581173±0.053041124 | Organismal Systems | Immune System | Antigen processing and presentation |
| ko04614 | 0±0 | 1.84147E-05±2.60423E-05 | 0.000117618±0.00012856 | 1.34505E-05±1.05518E-05 | 1.56393E-06±2.21173E-06 | 3.14964E-05±3.53918E-05 | Organismal Systems | Endocrine System | Renin-angiotensin system |
| ko04620 | 0±0 | 0±0 | 5.47E-06±7.73215E-06 | 6.24E-06±8.82838E-06 | 6.3758E-08±9.01675E-08 | 1.26356E-06±1.78695E-06 | Organismal Systems | Immune System | Toll-like receptor signaling pathway |
| ko04621 | 0.350040982±0.040799993 | 0.317711952±0.030734358 | 0.343506524±0.05205638 | 0.331793674±0.057042087 | 0.279052849±0.021983065 | 0.282035469±0.052176522 | Organismal Systems | Immune System | NOD-like receptor signaling pathway |
| ko04622 | 0.000321382±0.000102896 | 1.30E-04±0.000156823 | 0.000144582±6.88926E-05 | 5.46059E-05±2.90146E-05 | 9.60E-05±8.75809E-05 | 0.00024528±0.000228226 | Organismal Systems | Immune System | RIG-I-like receptor signaling pathway |
| ko04626 | 0.593519515±0.074415191 | 0.516476726±0.059655339 | 0.492053823±0.035787539 | 0.494383808±0.018787753 | 0.372646716±0.02674845 | 0.569210981±0.110943379 | Organismal Systems | Environmental Adaptation | Plant-pathogen interaction |
| ko04630 | 0±0 | 0.000104846±0.000148275 | 1.24307E-06±1.75796E-06 | 0±0 | 6.83068E-06±9.66004E-06 | 5.45122E-05±6.70017E-05 | Environmental Information Processing | Signal Transduction | Jak-STAT signaling pathway |
| ko04640 | 0±0 | 9.69E-07±7.92123E-07 | 0±0 | 2.41308E-06±3.4126E-06 | 0±0 | 0±0 | Organismal Systems | Immune System | Hematopoietic cell lineage |
| ko04660 | 3.49E-05±3.41327E-05 | 3.33E-05±4.09439E-05 | 1.78645E-05±2.19665E-05 | 3.29771E-05±2.76504E-05 | 2.45E-07±3.4663E-07 | 1.09E-05±1.28158E-05 | Organismal Systems | Immune System | T cell receptor signaling pathway |
| ko04662 | 3.49E-05±3.41327E-05 | 3.33E-05±4.09439E-05 | 1.78645E-05±2.19665E-05 | 3.29771E-05±2.76504E-05 | 2.45E-07±3.4663E-07 | 9.63E-06±1.36155E-05 | Organismal Systems | Immune System | B cell receptor signaling pathway |
| ko04664 | 0±0 | 0±0 | 0±0 | 0±0 | 0±0 | 0±0 | Organismal Systems | Immune System | Fc epsilon RI signaling pathway |
| ko04666 | 0.026345042±0.01149647 | 0.020514176±0.003074642 | 0.013177818±0.002994199 | 0.009644404±0.003726574 | 0.029680174±0.000909451 | 0.021491089±0.01267041 | Organismal Systems | Immune System | Fc gamma R-mediated phagocytosis |
| ko04670 | 4.59E-05±1.96593E-06 | 6.21041E-05±8.78285E-05 | 1.15E-04±0.000148783 | 9.21E-05±0.000120039 | 0.00024583±2.75242E-05 | 2.60E-04±0.000168782 | Organismal Systems | Immune System | Leukocyte transendothelial migration |
| ko04710 | 0±0 | 2.90E-05±2.41417E-05 | 5.58E-06±3.49239E-06 | 1.32E-05±1.42608E-05 | 1.46E-05±1.98616E-05 | 0.000010152±1.43571E-05 | Organismal Systems | Environmental Adaptation | Circadian rhythm - mammal |
| ko04711 | 0±0 | 2.90E-05±2.41417E-05 | 2.34E-05±2.10815E-05 | 4.61E-05±4.14453E-05 | 1.81E-05±1.76656E-05 | 1.98E-05±1.40011E-05 | Organismal Systems | Environmental Adaptation | Circadian rhythm - fly |
| ko04712 | 0±0 | 0±0 | 0±0 | 0±0 | 0±0 | 0±0 | Organismal Systems | Environmental Adaptation | Circadian rhythm - plant |
| ko04720 | 0±0 | 2.56876E-07±3.63278E-07 | 3.26305E-06±4.61465E-06 | 0±0 | 6.82074E-07±5.25942E-07 | 1.83E-05±2.58399E-05 | Organismal Systems | Nervous System | Long-term potentiation |
| ko04722 | 0.00042328±0.000107265 | 0.003959246±0.005285638 | 1.76E-04±0.000155028 | 1.44E-04±3.65768E-05 | 2.74291E-06±3.0195E-06 | 2.13E-05±3.01585E-05 | Organismal Systems | Nervous System | Neurotrophin signaling pathway |
| ko04730 | 0±0 | 6.38E-05±3.92353E-05 | 3.56577E-05±3.84357E-05 | 0.000124146±0.000116918 | 3.56E-04±0.000359231 | 0.000533715±0.000348937 | Organismal Systems | Nervous System | Long-term depression |
| ko04740 | 0±0 | 6.65E-05±4.20965E-05 | 1.32E-05±1.61425E-05 | 1.02E-04±0.000106282 | 0.000361972±0.000334139 | 0.000493915±0.000313303 | Organismal Systems | Sensory System | Olfactory transduction |
| ko04742 | 0.000477823±1.16357E-05 | 0.00059842±0.000243707 | 0.000220472±3.29295E-05 | 0.000380595±0.000294558 | 9.00787E-05±9.06073E-05 | 6.89E-05±4.82955E-05 | Organismal Systems | Sensory System | Taste transduction |
| ko04744 | 0±0 | 4.14E-05±5.52103E-05 | 1.35381E-05±1.60202E-05 | 1.75808E-05±1.26761E-05 | 4.30E-05±2.92598E-05 | 6.03E-05±6.75834E-05 | Organismal Systems | Sensory System | Phototransduction |
| ko04745 | 0±0 | 0±0 | 0±0 | 1.59311E-06±2.25299E-06 | 6.00777E-07±8.49627E-07 | 4.79E-06±6.77585E-06 | Organismal Systems | Sensory System | Phototransduction - fly |
| ko04810 | 0.026289807±0.011583958 | 0.020498957±0.003069195 | 0.013197184±0.003045745 | 0.009623371±0.00369852 | 0.029833991±0.000957126 | 0.021679214±0.012749256 | Cellular Processes | Cell Motility | Regulation of actin cytoskeleton |
| ko04812 | 2.03672707±0.015604613 | 2.584863735±0.311597592 | 1.824805287±0.08494945 | 1.905335578±0.233484658 | 1.645814588±0.080333342 | 1.782545877±0.144148245 | Cellular Processes | Cell Motility | Cytoskeleton proteins |
| ko04910 | 0.483760374±0.065768668 | 0.49953966±0.055098726 | 0.784414085±0.04625112 | 0.654518659±0.07156636 | 0.53319389±0.038463809 | 0.762549614±0.106025837 | Organismal Systems | Endocrine System | Insulin signaling pathway |
| ko04912 | 6.96099E-05±5.3193E-05 | 1.58E-05±1.06284E-05 | 1.5373E-06±2.17407E-06 | 3.38492E-05±4.787E-05 | 1.29E-05±1.25204E-05 | 1.10E-05±4.77657E-06 | Organismal Systems | Endocrine System | GnRH signaling pathway |
| ko04914 | 0.396521159±0.046795299 | 0.367854839±0.035490848 | 0.387177998±0.060831026 | 0.38636855±0.078028633 | 0.325910492±0.022182261 | 0.309149793±0.058522049 | Organismal Systems | Endocrine System | Progesterone-mediated oocyte maturation |
| ko04916 | 0±0 | 0±0 | 1.19097E-05±1.46444E-05 | 2.19227E-05±1.83487E-05 | 9.54E-06±7.83849E-06 | 1.74E-05±1.13903E-05 | Organismal Systems | Endocrine System | Melanogenesis |
| ko04920 | 1.967138873±0.186960633 | 1.87923385±0.3555644 | 2.958009095±0.434669087 | 2.310251505±0.222267088 | 2.211349331±0.098826085 | 1.951539816±0.508095408 | Organismal Systems | Endocrine System | Adipocytokine signaling pathway |
| ko04930 | 0.123896116±0.01396814 | 0.072167582±0.028877514 | 0.024132264±0.011592324 | 0.032743131±0.006154384 | 0.048153511±0.018028795 | 0.050811228±0.015189162 | Human Diseases | Metabolic Diseases | Type II diabetes mellitus |
| ko04940 | 0.277542626±0.032001053 | 0.262963974±0.01300471 | 0.22645399±0.009145605 | 0.263024799±0.054434027 | 0.175426811±0.014819249 | 0.200513086±0.044365456 | Human Diseases | Metabolic Diseases | Type I diabetes mellitus |
| ko04950 | 0±0 | 0±0 | 0±0 | 0±0 | 0±0 | 0±0 | Human Diseases | Metabolic Diseases | Maturity onset diabetes of the young |
| ko04960 | 1.09E-06±1.08681E-06 | 4.05387E-06±5.73303E-06 | 3.12E-05±3.67606E-05 | 1.87E-05±1.35568E-05 | 1.52E-05±3.11629E-06 | 9.58E-06±5.82896E-06 | Organismal Systems | Excretory System | Aldosterone-regulated sodium reabsorption |
| ko04962 | 0.007237317±0.004168475 | 0.001271851±0.000672569 | 0.00876368±0.002741828 | 0.00644572±0.006174004 | 0.009151878±0.004865523 | 0.007111438±0.002556498 | Organismal Systems | Excretory System | Vasopressin-regulated water reabsorption |
| ko04964 | 0.011220805±0.005531408 | 0.006246085±0.000234683 | 0.007888497±0.002130654 | 0.006256117±0.002485161 | 0.007610319±0.001235314 | 0.00828195±0.001719855 | Organismal Systems | Excretory System | Proximal tubule bicarbonate reclamation |
| ko04966 | 0±0 | 0±0 | 0±0 | 5.50092E-07±7.77948E-07 | 1.54379E-06±2.18325E-06 | 1.30E-06±1.84247E-06 | Organismal Systems | Excretory System | Collecting duct acid secretion |
| ko04970 | 3.44E-05±8.7566E-06 | 0.000169138±3.98837E-05 | 0.001130487±0.000898479 | 0.001462096±0.001430961 | 0.000555241±0.000305972 | 0.000635162±0.000322294 | Organismal Systems | Digestive System | Salivary secretion |
| ko04971 | 0.000148349±0.00014492 | 0±0 | 1.55E-05±1.59523E-05 | 4.67E-05±2.77338E-05 | 2.45E-05±1.21465E-05 | 1.40E-05±1.19965E-06 | Organismal Systems | Digestive System | Gastric acid secretion |
| ko05010 | 0.19209866±0.019648155 | 0.197153403±0.042219228 | 0.171090284±0.050003604 | 0.158677812±0.017940373 | 0.062146201±0.012145931 | 0.079340174±0.013264699 | Human Diseases | Neurodegenerative Diseases | Alzheimer's disease |
| ko05012 | 0.003421064±0.000963623 | 0.003097228±0.001524926 | 0.000791241±9.55923E-05 | 0.000771199±0.000178476 | 2.89E-04±0.000176114 | 0.000417123±0.000302307 | Human Diseases | Neurodegenerative Diseases | Parkinson's disease |
| ko05014 | 0.003036988±0.001022548 | 0.003030784±0.000465718 | 0.003420703±0.00118494 | 0.002547662±0.00016393 | 0.000835893±0.000175953 | 0.004430723±0.000925847 | Human Diseases | Neurodegenerative Diseases | Amyotrophic lateral sclerosis (ALS) |
| ko05016 | 0.046530917±0.002159559 | 0.038503363±0.001808552 | 0.030394872±0.012772415 | 0.025643169±0.005395987 | 0.029582719±0.006248648 | 0.018997289±0.009154425 | Human Diseases | Neurodegenerative Diseases | Huntington's disease |
| ko05020 | 0.001833346±0.000289299 | 0.002348371±0.000552533 | 0.002523527±0.00132746 | 0.00318793±0.001863158 | 0.000767836±0.00050063 | 0.001977678±0.001157649 | Human Diseases | Neurodegenerative Diseases | Prion diseases |
| ko05100 | 0.185715816±0.005369862 | 0.231547816±0.037829569 | 0.244575093±0.020395416 | 0.340528968±0.155393409 | 0.147318305±0.044381581 | 0.175010781±0.007758089 | Human Diseases | Infectious Diseases | Bacterial invasion of epithelial cells |
| ko05110 | 0.001063842±0.000325257 | 0.004841989±0.002533927 | 0.001860609±0.000565676 | 0.004609564±0.00088876 | 0.00059258±0.000346145 | 0.007018883±0.005754145 | Human Diseases | Infectious Diseases | Vibrio cholerae infection |
| ko05111 | 0.217136353±0.004618246 | 0.179380832±0.000330719 | 0.191568824±0.018739222 | 0.178504664±0.02725606 | 0.166461236±0.01981843 | 0.132908793±0.050335109 | Human Diseases | Infectious Diseases | Vibrio cholerae pathogenic cycle |
| ko05120 | 0.940941431±0.077019442 | 0.903473932±0.05962566 | 1.145768092±0.094529831 | 1.116314059±0.160254462 | 0.947769039±0.085918704 | 0.98643831±0.135951778 | Human Diseases | Infectious Diseases | Epithelial cell signaling in Helicobacter pylori infection |
| ko05130 | 0.002390118±0.000273433 | 0.009684866±0.00386806 | 0.008751694±0.002050652 | 0.0080504±0.003213531 | 0.009039104±0.002307426 | 0.013433862±0.007261247 | Human Diseases | Infectious Diseases | Pathogenic Escherichia coli infection |
| ko05131 | 1.49843E-05±1.49843E-05 | 1.12051E-06±1.58464E-06 | 2.00E-04±0.000215575 | 1.16E-04±9.0009E-05 | 0.000157029±6.33327E-05 | 1.47E-04±0.000144353 | Human Diseases | Infectious Diseases | Shigellosis |
| ko05140 | 0±0 | 0±0 | 0±0 | 0±0 | 0±0 | 0±0 | Human Diseases | Infectious Diseases | Leishmaniasis |
| ko05142 | 2.25E-04±0.000142746 | 9.65726E-05±7.69886E-05 | 0.002370136±0.00145721 | 0.002264849±0.001673696 | 3.29E-05±2.84286E-05 | 0.000396312±0.00046917 | Human Diseases | Infectious Diseases | Chagas disease |
| ko05144 | 0.000889237±0.000601178 | 0.00086495±0.000238056 | 0.000699397±0.0005688 | 0.000993179±0.000527041 | 9.50E-05±7.3791E-05 | 1.18E-03±0.001136076 | Human Diseases | Infectious Diseases | Malaria |
| ko05146 | 0.091503218±0.003282517 | 0.082574983±0.019091973 | 0.230165988±0.050712092 | 0.176093719±0.084008785 | 0.228284298±0.019181112 | 0.134704644±0.036641742 | Human Diseases | Infectious Diseases | Amoebiasis |
| ko05200 | 0.3605443±0.044274656 | 0.335529137±0.030822453 | 0.350626013±0.050556291 | 0.337612064±0.053919676 | 0.282060261±0.021405012 | 0.290278682±0.05084618 | Human Diseases | Cancers | Pathways in cancer |
| ko05210 | 0.000307108±0.000307108 | 5.70001E-05±6.25887E-05 | 0.000174592±0.000243534 | 0.000226232±0.000291712 | 3.15E-04±0.000276786 | 0.000557049±0.000569399 | Human Diseases | Cancers | Colorectal cancer |
| ko05211 | 0.009557854±0.002526427 | 0.015933281±0.001937836 | 0.006032146±0.001506536 | 0.004105511±0.002982342 | 0.000311109±0.000255825 | 0.006347945±0.002061665 | Human Diseases | Cancers | Renal cell carcinoma |
| ko05212 | 6.59536E-05±6.59536E-05 | 0.00061237±0.000757849 | 0.000134906±0.000114002 | 0.00068993±0.000428277 | 0.001825572±0.000334716 | 0.000571194±0.000431948 | Human Diseases | Cancers | Pancreatic cancer |
| ko05213 | 0±0 | 5.42454E-05±5.87571E-05 | 2.17E-05±2.11391E-05 | 3.87E-05±2.31401E-05 | 3.26447E-05±2.3182E-05 | 4.32E-05±1.68659E-05 | Human Diseases | Cancers | Endometrial cancer |
| ko05214 | 0±0 | 0±0 | 0±0 | 0±0 | 0±0 | 4.30E-06±6.08572E-06 | Human Diseases | Cancers | Glioma |
| ko05215 | 0.34959165±0.041136703 | 0.317661813±0.03066166 | 0.344139415±0.052708798 | 0.332215651±0.056768507 | 0.278992915±0.021988355 | 0.28238123±0.052389966 | Human Diseases | Cancers | Prostate cancer |
| ko05216 | 1.32122E-05±1.32122E-05 | 9.12926E-06±1.29107E-05 | 2.65046E-05±3.74832E-05 | 3.84743E-05±5.18123E-05 | 5.08E-06±3.81507E-06 | 2.27538E-05±3.08138E-05 | Human Diseases | Cancers | Thyroid cancer |
| ko05217 | 6.00411E-05±6.00411E-05 | 2.47E-06±3.49391E-06 | 0.000019658±2.1025E-05 | 3.91906E-05±3.44255E-05 | 1.37694E-06±1.94728E-06 | 9.63E-06±1.36155E-05 | Human Diseases | Cancers | Basal cell carcinoma |
| ko05218 | 0±0 | 0±0 | 0±0 | 0±0 | 0±0 | 0±0 | Human Diseases | Cancers | Melanoma |
| ko05219 | 0.000153505±0.00014326 | 3.50E-04±0.000408462 | 3.27E-05±1.39796E-05 | 1.06E-04±4.38212E-05 | 1.80033E-06±2.54605E-06 | 6.88921E-05±4.87302E-05 | Human Diseases | Cancers | Bladder cancer |
| ko05220 | 6.82E-06±6.82144E-06 | 0±0 | 6.57916E-07±9.30433E-07 | 3.55669E-06±5.02993E-06 | 0±0 | 0±0 | Human Diseases | Cancers | Chronic myeloid leukemia |
| ko05221 | 0±0 | 0±0 | 0±0 | 0±0 | 1.38186E-05±1.16978E-05 | 5.85553E-07±8.28097E-07 | Human Diseases | Cancers | Acute myeloid leukemia |
| ko05222 | 0.000073715±3.03586E-05 | 8.00E-05±1.43638E-05 | 1.63E-05±1.96161E-05 | 1.77E-05±1.89373E-05 | 2.53E-05±1.90832E-05 | 3.72E-05±2.71536E-05 | Human Diseases | Cancers | Small cell lung cancer |
| ko05223 | 0±0 | 0±0 | 3.24E-06±4.10619E-06 | 3.61E-06±5.09902E-06 | 0±0 | 0±0 | Human Diseases | Cancers | Non-small cell lung cancer |
| ko05322 | 2.28628E-07±2.28628E-07 | 1.21E-04±0.00015799 | 0.00040546±0.000231255 | 0.000310516±6.32031E-05 | 0.000108933±7.42725E-05 | 0.000321863±0.000267405 | Human Diseases | Immune System Diseases | Systemic lupus erythematosus |
| ko05340 | 0.094420486±0.013631006 | 0.064316113±0.014407452 | 0.089977338±0.014378488 | 0.08873151±0.018710142 | 0.077957902±0.00548882 | 0.071514577±0.004125946 | Human Diseases | Immune System Diseases | Primary immunodeficiency |
| ko05410 | 7.84972E-05±3.54449E-05 | 5.88E-04±0.000704537 | 0.000359067±0.00024859 | 0.000428688±0.000209247 | 0.0001251±0.000150051 | 9.59E-05±5.09808E-05 | Human Diseases | Cardiovascular Diseases | Hypertrophic cardiomyopathy (HCM) |
| ko05412 | 3.21E-05±1.09731E-05 | 8.51E-05±7.69157E-06 | 0.000101537±2.2082E-05 | 9.21911E-05±7.48453E-05 | 9.24237E-05±0.000107231 | 4.08E-05±2.90726E-05 | Human Diseases | Cardiovascular Diseases | Arrhythmogenic right ventricular cardiomyopathy (ARVC) |
| ko05414 | 7.84972E-05±3.54449E-05 | 5.82E-04±0.000695851 | 0.000251794±0.000132249 | 0.000418783±0.000215357 | 1.13E-04±0.000117998 | 6.38E-05±2.77741E-05 | Human Diseases | Cardiovascular Diseases | Dilated cardiomyopathy (DCM) |
| ko05416 | 0.06697481±0.006774493 | 0.062098758±0.02843331 | 0.0695934±0.012375633 | 0.061336711±0.03274293 | 0.086463756±0.031517323 | 0.045922689±0.029619363 | Human Diseases | Cardiovascular Diseases | Viral myocarditis |

## Table S18. KEGG enrichment results for the rumen metagenome in two phases. “Up” represents those pathways are enriched in the second phase and “down” represents those pathways are enriched in the first phase.

| **Phase** | **KO** | **Information** | ***P* value** | **Adjusted *P* value** |
| --- | --- | --- | --- | --- |
| up | ko00010 | Glycolysis / Gluconeogenesis | 1.61E-08 | 3.50E-06 |
| up | ko00190 | Oxidative phosphorylation | 2.73E-08 | 5.93E-06 |
| up | ko00240 | Pyrimidine metabolism | 1.69E-05 | 3.66E-03 |
| up | ko00260 | Glycine, serine and threonine metabolism | 8.47E-09 | 1.84E-06 |
| up | ko00270 | Cysteine and methionine metabolism | 6.84E-06 | 1.48E-03 |
| up | ko00310 | Lysine degradation | 8.06E-10 | 1.75E-07 |
| up | ko00620 | Pyruvate metabolism | 1.25E-05 | 2.71E-03 |
| up | ko00632 | Benzoate degradation via CoA ligation | 3.07E-06 | 6.67E-04 |
| up | ko00650 | Butanoate metabolism | 1.37E-14 | 2.97E-12 |
| up | ko02020 | Two-component system | 1.21E-06 | 2.62E-04 |
| up | ko03010 | Ribosome | 2.03E-05 | 4.41E-03 |
| down | ko00250 | Alanine, aspartate and glutamate metabolism | 7.75E-11 | 1.68E-08 |
| down | ko00440 | Phosphonate and phosphinate metabolism | 1.14E-08 | 2.46E-06 |
| down | ko00910 | Nitrogen metabolism | 8.96E-07 | 1.95E-04 |
| down | ko00330 | Arginine and proline metabolism | 1.31E-06 | 2.85E-04 |
| down | ko00312 | beta-Lactam resistance | 1.05E-04 | 2.27E-02 |
| down | ko00363 | Bisphenol A degradation | 1.05E-04 | 2.27E-02 |
| down | ko00471 | D-Glutamine and D-glutamate metabolism | 1.05E-04 | 2.27E-02 |
| down | ko00565 | Ether lipid metabolism | 1.05E-04 | 2.27E-02 |
| down | ko00631 | 1,2-Dichloroethane degradation | 1.05E-04 | 2.27E-02 |
| down | ko00642 | Ethylbenzene degradation | 1.05E-04 | 2.27E-02 |
| down | ko04031 | GTP-binding proteins | 1.05E-04 | 2.27E-02 |
| down | ko04062 | Chemokine signaling pathway | 1.05E-04 | 2.27E-02 |
| down | ko04115 | p53 signaling pathway | 1.05E-04 | 2.27E-02 |
| down | ko04340 | Hedgehog signaling pathway | 1.05E-04 | 2.27E-02 |
| down | ko04520 | Adherens junction | 1.05E-04 | 2.27E-02 |
| down | ko04540 | Gap junction | 1.05E-04 | 2.27E-02 |
| down | ko04621 | NOD-like receptor signaling pathway | 1.05E-04 | 2.27E-02 |
| down | ko04622 | RIG-I-like receptor signaling pathway | 1.05E-04 | 2.27E-02 |
| down | ko04744 | Phototransduction | 1.05E-04 | 2.27E-02 |
| down | ko04962 | Vasopressin-regulated water reabsorption | 1.05E-04 | 2.27E-02 |
| down | ko00563 | Glycosylphosphatidylinositol(GPI)-anchor biosynthesis | 1.25E-04 | 2.72E-02 |
| down | ko00590 | Arachidonic acid metabolism | 1.25E-04 | 2.72E-02 |
| down | ko00622 | Toluene and xylene degradation | 1.25E-04 | 2.72E-02 |
| down | ko00625 | Tetrachloroethene degradation | 1.25E-04 | 2.72E-02 |
| down | ko00627 | 1,4-Dichlorobenzene degradation | 1.25E-04 | 2.72E-02 |
| down | ko03022 | Basal transcription factors | 1.25E-04 | 2.72E-02 |
| down | ko04010 | MAPK signaling pathway | 1.25E-04 | 2.72E-02 |
| down | ko04091 | Glycan bindng proteins | 1.25E-04 | 2.72E-02 |
| down | ko04260 | Cardiac muscle contraction | 1.25E-04 | 2.72E-02 |
| down | ko04270 | Vascular smooth muscle contraction | 1.25E-04 | 2.72E-02 |
| down | ko04360 | Axon guidance | 1.25E-04 | 2.72E-02 |
| down | ko04920 | Adipocytokine signaling pathway | 1.25E-04 | 2.72E-02 |
| down | ko00072 | Synthesis and degradation of ketone bodies | 1.46E-04 | 3.17E-02 |
| down | ko00120 | Primary bile acid biosynthesis | 1.46E-04 | 3.17E-02 |
| down | ko00311 | Penicillin and cephalosporin biosynthesis | 1.46E-04 | 3.17E-02 |
| down | ko00430 | Taurine and hypotaurine metabolism | 1.46E-04 | 3.17E-02 |
| down | ko00628 | Fluorene degradation | 1.46E-04 | 3.17E-02 |
| down | ko00641 | 3-Chloroacrylic acid degradation | 1.46E-04 | 3.17E-02 |
| down | ko00791 | Atrazine degradation | 1.46E-04 | 3.17E-02 |
| down | ko00940 | Phenylpropanoid biosynthesis | 1.46E-04 | 3.17E-02 |
| down | ko04150 | mTOR signaling pathway | 1.46E-04 | 3.17E-02 |
| down | ko04310 | Wnt signaling pathway | 1.46E-04 | 3.17E-02 |
| down | ko04722 | Neurotrophin signaling pathway | 1.46E-04 | 3.17E-02 |
| down | ko00603 | Glycosphingolipid biosynthesis - globo series | 1.67E-04 | 3.63E-02 |
| down | ko00626 | Naphthalene and anthracene degradation | 1.67E-04 | 3.63E-02 |
| down | ko00980 | Metabolism of xenobiotics by cytochrome P450 | 1.67E-04 | 3.63E-02 |
| down | ko00982 | Drug metabolism - cytochrome P450 | 1.67E-04 | 3.63E-02 |
| down | ko01040 | Biosynthesis of unsaturated fatty acids | 1.67E-04 | 3.63E-02 |
| down | ko03050 | Proteasome | 1.67E-04 | 3.63E-02 |
| down | ko04011 | MAPK signaling pathway - yeast | 1.67E-04 | 3.63E-02 |
| down | ko04070 | Phosphatidylinositol signaling system | 1.67E-04 | 3.63E-02 |
| down | ko04515 | Cell adhesion molecules (CAMs) | 1.67E-04 | 3.63E-02 |
| down | ko04612 | Antigen processing and presentation | 1.67E-04 | 3.63E-02 |
| down | ko05014 | Amyotrophic lateral sclerosis (ALS) | 1.67E-04 | 3.63E-02 |
| down | ko00281 | Geraniol degradation | 1.88E-04 | 4.08E-02 |
| down | ko00401 | Novobiocin biosynthesis | 1.88E-04 | 4.08E-02 |
| down | ko00643 | Styrene degradation | 1.88E-04 | 4.08E-02 |
| down | ko00780 | Biotin metabolism | 1.88E-04 | 4.08E-02 |
| down | ko00930 | Caprolactam degradation | 1.88E-04 | 4.08E-02 |
| down | ko00950 | Isoquinoline alkaloid biosynthesis | 1.88E-04 | 4.08E-02 |
| down | ko03320 | PPAR signaling pathway | 1.88E-04 | 4.08E-02 |
| down | ko04530 | Tight junction | 1.88E-04 | 4.08E-02 |
| down | ko04914 | Progesterone-mediated oocyte maturation | 1.88E-04 | 4.08E-02 |
| down | ko00510 | N-Glycan biosynthesis | 2.09E-04 | 4.54E-02 |
| down | ko00531 | Glycosaminoglycan degradation | 2.09E-04 | 4.54E-02 |
| down | ko00535 | Proteoglycans | 2.09E-04 | 4.54E-02 |
| down | ko00660 | C5-Branched dibasic acid metabolism | 2.09E-04 | 4.54E-02 |
| down | ko00830 | Retinol metabolism | 2.09E-04 | 4.54E-02 |
| down | ko00906 | Carotenoid biosynthesis | 2.09E-04 | 4.54E-02 |
| down | ko04970 | Salivary secretion | 2.09E-04 | 4.54E-02 |
| down | ko00460 | Cyanoamino acid metabolism | 2.30E-04 | 4.99E-02 |
| down | ko00511 | Other glycan degradation | 2.30E-04 | 4.99E-02 |
| down | ko00521 | Streptomycin biosynthesis | 2.30E-04 | 4.99E-02 |
| down | ko04516 | CAM ligands | 2.30E-04 | 4.99E-02 |
| down | ko04610 | Complement and coagulation cascades | 2.30E-04 | 4.99E-02 |
| down | ko04666 | Fc gamma R-mediated phagocytosis | 2.30E-04 | 4.99E-02 |
| down | ko04810 | Regulation of actin cytoskeleton | 2.30E-04 | 4.99E-02 |

## Table S19. Genera in each microbial module.

[See the excel file]

## Table S20. Spearman’s correlation coefficient and *p* value of the first eigenvector from the PCA of each host module with microbial alpha diversity index.

| **Module** | **Spearman's correlation coefficient** | ***P* value** |
| --- | --- | --- |
| R1 | -0.01225 | 0.962768 |
| R2 | -0.32843 | 0.198069 |
| R3 | 0.281863 | 0.27306 |
| R4 | -0.18627 | 0.474095 |
| R5 | -0.12255 | 0.639378 |
| R6 | -0.09069 | 0.729235 |
| R7 | -0.56373 | 0.018435 |
| R8 | -0.55637 | 0.020371 |
| R9 | -0.56863 | 0.017227 |
| R10 | -0.30882 | 0.227786 |
| R11 | -0.35049 | 0.167815 |
| R12 | -0.16667 | 0.522603 |
| R13 | 0.492647 | 0.044525 |
| R14 | -0.41176 | 0.10054 |
| R15 | 0.313725 | 0.220104 |

## Table S21 Ingredients and nutrients of the experimental diets. The main nutrients of alfalfa: crude protein 18.1%, lysine 1.76%, methionine + cystine 0.46%, calcium 1.5%, phosphorus 0.49%.

| **Ingredients** | **Content (%)** | **Nutrient level** | **Content** |
| --- | --- | --- | --- |
| Corn | 23.30 | Dry matter (DM), % | 89.09 |
| Soybean meal | 13.75 | Digestible energy(MJ/kg） | 8.50 |
| Rice straw | 20.40 | Crude protein, % | 10.00 |
| Straw | 39.60 | crude fat, % | 2.02 |
| Limestone | 0.55 | crude fiber, % | 17.28 |
| Ca(HCO_3_)_2_ | 0.90 | NFE, % | 49.20 |
| NaCl | 0.50 | Ash, % | 8.36 |
| Premix | 1.00 | Calcium, % | 0.70 |
| Total | 100 | Phosphorus, % | 0.40 |
